# Association of Intensive vs Standard Blood Pressure Control With Regional Changes in Cerebral Small Vessel Disease Biomarkers: Post Hoc Secondary Analysis of the SPRINT MIND Randomized Clinical Trial

**DOI:** 10.1001/jamanetworkopen.2023.1055

**Published:** 2023-03-01

**Authors:** Tanweer Rashid, Karl Li, Jon B. Toledo, Ilya Nasrallah, Nicholas M. Pajewski, Sudipto Dolui, John Detre, David A. Wolk, Hangfan Liu, Susan R. Heckbert, R. Nick Bryan, Jeff Williamson, Christos Davatzikos, Sudha Seshadri, Lenore J. Launer, Mohamad Habes

**Affiliations:** 1 Neuroimage Analytics Laboratory and the Biggs Institute Neuroimaging Core, Glenn Biggs Institute for Alzheimer’s and Neurodegenerative Diseases, University of Texas Health Science Center San Antonio, San Antonio; 2 Department of Neurology, University of Florida, Gainesville; 3 Department of Neurology, Houston Methodist Hospital, Houston, Texas; 4 Department of Radiology, Hospital of the University of Pennsylvania, Philadelphia; 5 Center for Biomedical Image Computing and Analytics, University of Pennsylvania, Philadelphia; 6 Department of Biostatistics and Data Science, Wake Forest School of Medicine, Winston-Salem, North Carolina; 7 Department of Neurology, University of Pennsylvania, Philadelphia; 8 Department of Epidemiology, University of Washington, Seattle; 9 Section of Gerontology and Geriatric Medicine, Department of Internal Medicine, Wake Forest School of Medicine, Winston-Salem, North Carolina; 10 Intramural Research Program, Laboratory of Epidemiology and Population Sciences, National Institute on Aging, National Institutes of Health, Bethesda, Maryland

## Abstract

**Importance:**

Little is known about the associations of strict blood pressure (BP) control with microstructural changes in small vessel disease markers.

**Objective:**

To investigate the regional associations of intensive vs standard BP control with small vessel disease biomarkers, such as white matter lesions (WMLs), fractional anisotropy (FA), mean diffusivity (MD), and cerebral blood flow (CBF).

**Design, Setting, and Participants:**

The Systolic Blood Pressure Intervention Trial (SPRINT) is a multicenter randomized clinical trial that compared intensive systolic BP (SBP) control (SBP target <120 mm Hg) vs standard control (SBP target <140 mm Hg) among participants aged 50 years or older with hypertension and without diabetes or a history of stroke. The study began randomization on November 8, 2010, and stopped July 1, 2016, with a follow-up duration of approximately 4 years. A total of 670 and 458 participants completed brain magnetic resonance imaging at baseline and follow-up, respectively, and comprise the cohort for this post hoc analysis. Statistical analyses for this post hoc analysis were performed between August 2020 and October 2022.

**Interventions:**

At baseline, 355 participants received intensive SBP treatment and 315 participants received standard SBP treatment.

**Main Outcomes and Measures:**

The main outcomes were regional changes in WMLs, FA, MD (in white matter regions of interest), and CBF (in gray matter regions of interest).

**Results:**

At baseline, 355 participants (mean [SD] age, 67.7 [8.0] years; 200 men [56.3%]) received intensive BP treatment and 315 participants (mean [SD] age, 67.0 [8.4] years; 199 men [63.2%]) received standard BP treatment. Intensive treatment was associated with smaller mean increases in WML volume compared with standard treatment (644.5 mm^3^ vs 1258.1 mm^3^). The smaller mean increases were observed specifically in the deep white matter regions of the left anterior corona radiata (intensive treatment, 30.3 mm^3^ [95% CI, 16.0-44.5 mm^3^]; standard treatment, 80.5 mm^3^ [95% CI, 53.8-107.2 mm^3^]), left tapetum (intensive treatment, 11.8 mm^3^ [95% CI, 4.4-19.2 mm^3^]; standard treatment, 27.2 mm^3^ [95% CI, 19.4-35.0 mm^3^]), left superior fronto-occipital fasciculus (intensive treatment, 3.2 mm^3^ [95% CI, 0.7-5.8 mm^3^]; standard treatment, 9.4 mm^3^ [95% CI, 5.5-13.4 mm^3^]), left posterior corona radiata (intensive treatment, 26.0 mm^3^ [95% CI, 12.9-39.1 mm^3^]; standard treatment, 52.3 mm^3^ [95% CI, 34.8-69.8 mm^3^]), left splenium of the corpus callosum (intensive treatment, 45.4 mm^3^ [95% CI, 25.1-65.7 mm^3^]; standard treatment, 83.0 mm^3^ [95% CI, 58.7-107.2 mm^3^]), left posterior thalamic radiation (intensive treatment, 53.0 mm^3^ [95% CI, 29.8-76.2 mm^3^]; standard treatment, 106.9 mm^3^ [95% CI, 73.4-140.3 mm^3^]), and right posterior thalamic radiation (intensive treatment, 49.5 mm^3^ [95% CI, 24.3-74.7 mm^3^]; standard treatment, 102.6 mm^3^ [95% CI, 71.0-134.2 mm^3^]).

**Conclusions and Relevance:**

This study suggests that intensive BP treatment, compared with standard treatment, was associated with a slower increase of WMLs, improved diffusion tensor imaging, and FA and CBF changes in several brain regions that represent vulnerable areas that may benefit from more strict BP control.

**Trial Registration:**

ClinicalTrials.gov Identifier: NCT01206062

## Key Points

**Question:**

How is intensive vs standard blood pressure treatment associated with small vessel disease biomarkers, such as white matter lesions, fractional anisotropy, mean diffusivity, and cerebral blood flow?

**Findings:**

In this post hoc analysis of a randomized clinical trial with 458 participants, intensive blood pressure treatment was associated with reduced progression of white matter lesions, notably in deep white matter tracts, and supported by corresponding changes in fractional anisotropy and mean diffusivity. Intensive blood pressure treatment was also associated with improved cerebral blood flow in the occipital lobe, parietal lobule, frontal gyrus, and cuneus.

**Meaning:**

Findings suggest that intensive blood pressure treatment was associated with a slower increase of white matter lesions and improved cerebral blood flow in areas that may be vulnerable to hypertension.

## Teaser

This post hoc secondary analysis of the SPRINT MIND randomized clinical trial investigates the regional associations of intensive vs standard blood pressure control with small vessel disease biomarkers, such as white matter lesions, fractional anisotropy, mean diffusivity, and cerebral blood flow.

## Introduction

Hypertension is a major risk factor for cardiovascular disease and stroke,^1^ with a 45.6% prevalence among US adults and an annual cost of $52 billion.^2^ Clinical trials studying the efficacy of antihypertensive drugs have demonstrated a decrease in the incidence of cardiovascular disease, stroke, myocardial infarction, and heart failure associated with these agents.^3^ Small vessel disease and hypertension-related changes can be seen primarily in T2-weighted or fluid-attenuated inversion recovery sequences manifesting as white matter lesions (WMLs)^4,5,6^ or in diffusion tensor imaging (DTI) sequences as disruption or damage of fiber tracts.^7,8,9,10^ These changes can also be associated with Alzheimer disease, non–Alzheimer disease cognitive impairment, and advanced brain aging.^4,11,12^ Through midlife, the WML burden is greater in the frontal regions, while most parietotemporal WML burdens develop primarily after the sixth decade of life.^11^ Neuroimaging and histopathologic studies suggest that frontal lesions have a more vascular origin, while parietotemporal WMLs are more closely associated with neurodegenerative disease–related changes.^10,13,14,15,16,17^

Fractional anisotropy (FA) and mean diffusivity (MD) are quantitative metrics calculated from diffusion magnetic resonance imaging (MRI). Fractional anisotropy describes the directional coherence of water diffusion in tissue and is generally interpreted as a quantitative biomarker for white matter (WM) integrity, while MD describes the overall diffusion of water molecules and typically has a low value in WM.^18^ Cerebral blood flow (CBF), calculated^19,20^ from arterial spin labeling MRI, is a relative measure of the volume of blood flowing through tissue over time.

Previous randomized, prospective trials with few participants and a brief trial duration have assessed the effect of blood pressure (BP) control on cerebral perfusion, with mixed findings.^21,22,23^ Large-scale clinical trials with multimodal imaging are therefore essential to report robust findings on the precise improvement of brain regions with intensive hypertension treatment, which could explain brain reserve and cognition maintenance.

We hypothesized that antihypertensive treatment would be associated with protection of frontal brain regions. Toward that, we compared regional WML changes throughout the brain between a group receiving intensive BP control and a group receiving standard BP control. We further characterized the nature of the WM microstructural changes using regional DTI analysis and investigated as a secondary hypothesis that intensive hypertension management would be associated with regional improvement in CBF. We used the Systolic Blood Pressure Intervention Trial (SPRINT) Memory and Cognition in Decreased Hypertension (MIND) clinical trial, which is a large-scale multisite landmark study that demonstrated improved cognition and brain reserve in the group that received intensive systolic BP (SBP) control. We expand on previous reports^24,25,26,27,28^ demonstrating the regional changes in WM integrity and CBF that may be associated with hypertension control.

## Methods

### Trial Design

The SPRINT trial was designed to examine the effects of intensive SBP control vs standard SBP control on cardiovascular disease as the primary outcome and kidney function, dementia or decline in cognitive function, and small-vessel cerebral ischemic disease as secondary outcomes, as previously described.^24,29^ The trial and substudies were approved by the institutional review board of each participating site (University of Alabama at Birmingham, Boston University, Vanderbilt University, Wake Forest University, University of Miami, University of Pennsylvania, and Case Western Reserve University), and each participant provided written informed consent. Enrollment began on November 8, 2010, and ended July 1, 2016. The trial protocol is provided in Supplement 1. Additional details regarding the SPRINT trial design can be found in Supplement 1. This study was conducted in accordance with the Consolidated Standards of Reporting Trials (CONSORT) reporting guideline.

### Study Participants

A total of 9361 participants were randomized to receive either a standard treatment strategy with a target SBP of less than 140 mm Hg (n = 4683) or an intensive treatment strategy with a target SBP of less than 120 mm Hg (n = 4678). Additional details on participants are provided in the trial protocol in Supplement 1.

Race was self-reported by participants as Black, Hispanic, White, and other (Asian, American Indian or Native Alaskan, Native Hawaiian or Pacific Islander, or other races). Black race includes Hispanic Black and Black as part of multiracial identification. Hispanic race and ethnicity encompasses a self-report of being of Spanish, Hispanic, or Latino origin, independent of any other race and ethnicity designation.

### Analytical Sample

**Figure 1.**
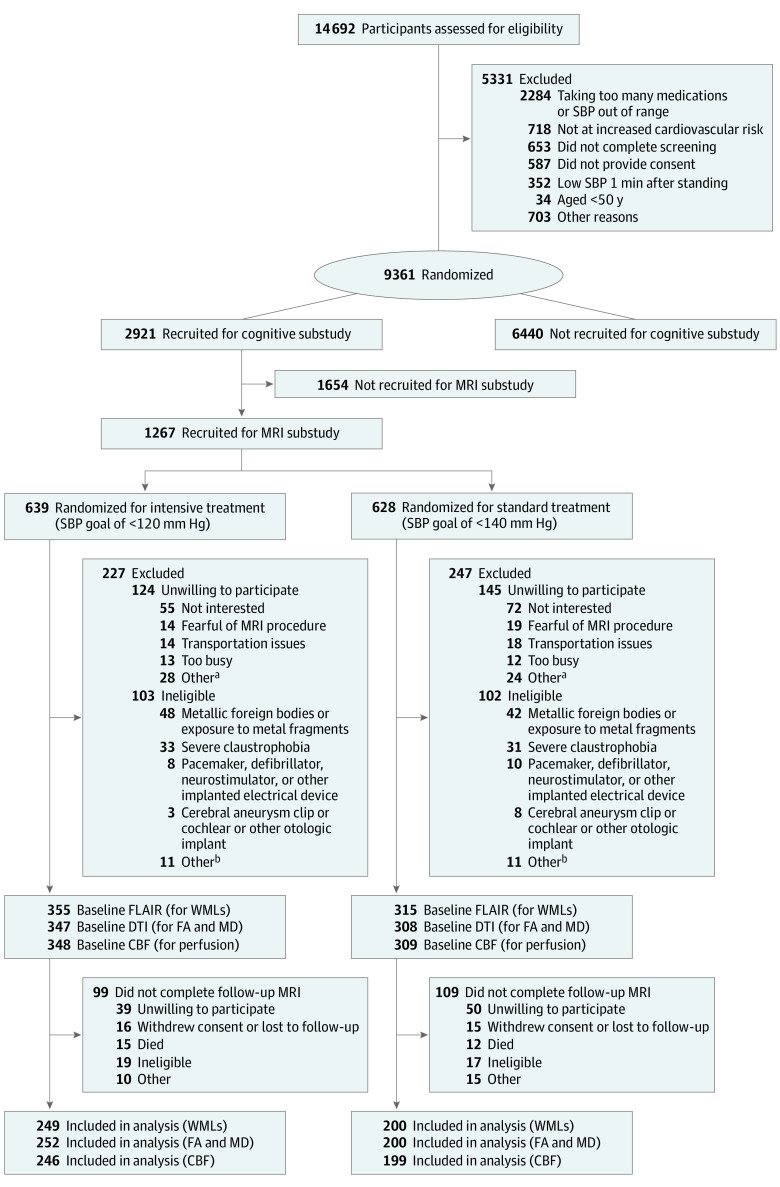
Flow Diagram Showing the Randomization Eligibility, Inclusion, and Exclusion of the Participants for the Magnetic Resonance Imaging (MRI) Substudy CBF indicates cerebral blood flow; DTI, diffusion tensor imaging; FA, fractional anisotropy; FLAIR, fluid-attenuated inversion recovery; MD, mean diffusivity; SBP, systolic blood pressure; and WMLs, white matter lesions. ^a^Other reasons for unwillingness to participate include participant could not lie flat for extended periods (n = 7), participant reported metal in body before formal screening (n = 8), participant concerns about stent or other cardiac device (n = 8), concerns about body size (n = 2), other reasons (n = 7), and unknown reason (n = 22). ^b^Other reasons for ineligibility include participant concerns about stent or other cardiac device (n = 8), use of pain pump (n = 2), prolonged hospitalization (n = 2), participant declined consent (n = 2), participant too large for MRI scanner (n = 1), and other reasons (n = 7).

Of the 9361 randomized participants, 2921 were selected for a post hoc substudy of cognitive outcomes^30^ and were administered a comprehensive cognitive battery at baseline and follow-up. Of these, 1267 participants were invited to participate in the post hoc SPRINT MIND substudy to undergo brain MRI scanning at baseline and 48-month follow-up at 1 of 7 designated MRI sites (Figure 1). Inclusion and exclusion criteria for participants in the MRI substudy are discussed in the trial protocol in Supplement 1.

### Magnetic Resonance Imaging

The SPRINT MIND study acquired brain MRI scans of participants at baseline and 48-month follow-up after randomization. Imaging parameters are detailed in eTable 1 in Supplement 2. All image analysts were blinded to treatment group. We worked with the same WML segmentations used in previous SPRINT studies.^26,27,28^ These WML segmentations were derived with a deep learning method^31^ and applied in large cohort studies^11,32,33,34^ with similar settings. We calculated the total WMLs, mean FA, and MD for all the WM regions of interest (ROIs) of the Type III WM parcellation map.^35^ The WM delineations of the Type III WM parcellation map atlas were described as highly likely to be WM with minimum contamination from gray matter (GM) and cerebrospinal fluid.^35^ In the case of FA and MD, we calculated the mean in only WM ROIs because water diffusion is more anisotropic in these regions than in GM and cerebrospinal fluid.^36,37^ The mean cerebrospinal fluid was calculated for GM ROIs only because the signal in these regions tends to be more reliable and stronger^38,39^ than in WM ROIs.^40^ Detailed descriptions on WML, FA, MD, and CBF calculations are provided in eMethods 1 in Supplement 2. Thirteen participants with baseline MRI scans had structural brain lesions and 3 participants at follow-up had large strokes; these participants were excluded from the analysis.

### Statistical Analysis

Statistical analyses were performed between August 2020 and October 2022. We used linear mixed-effects models to estimate changes in MRI measures (FA, MD, WMLs, and CBF) for the treatment groups, with participant and MRI facility treated as random intercepts. Treatment group, intracranial volume, age, sex, and time since randomization (in days) were treated as fixed effects. For WMLs, an inverse hyperbolic sine transform—asinh: *f*(*x*) = log [*x* + (*x*^2^ + 1)^0.5^]—was used to accommodate zero values.^41^

**Figure 2.**
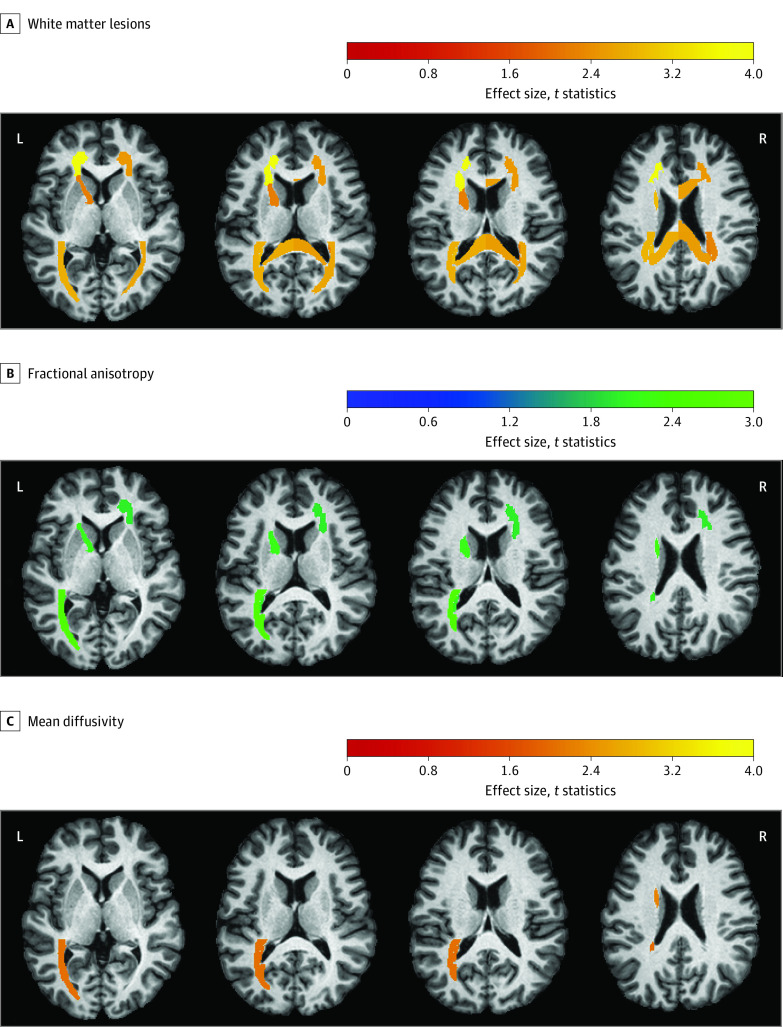
Patterns of Changes in the Type III White Matter Parcellation Map–Derived Deep White Matter Regions of Interest When Comparing Intensive Treatment With Standard Treatment A, Changes in white matter lesions. B, Changes in diffusion tensor imaging (DTI) fractional anisotropy. C, Changes in DTI mean diffusivity. The red-yellow color scale indicates effect size; that is, smaller increase over time for intensive treatment compared with standard treatment. The blue-green color scale indicates effect size; that is, larger increase for intensive treatment compared with standard treatment. Changes are significant at *P* < .05, uncorrected. L indicates left; R, right.

**Figure 3.**
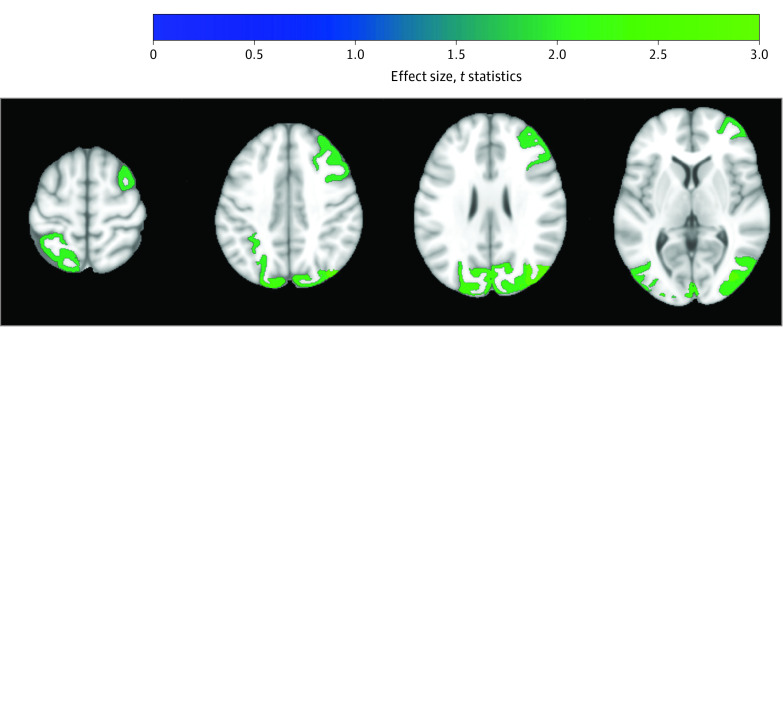
Patterns of Changes in the Multi-Atlas Region Segmentation Utilizing Ensembles–Derived Gray Matter Regions of Interest When Comparing Cerebral Blood Flow Between Intensive Treatment and Standard Treatment The blue-green color scale indicates effect size; that is, larger increase for intensive treatment compared with standard treatment. Changes are significant at *P* < .05, uncorrected.

All statistical analyses were performed using R, version 3.6 (R Group for Statistical Computing).^42^ All hypothesis tests were 2-sided, and *P* < .05 was considered statistically significant. *P* values, uncorrected and after adjustments for multiple comparisons (with false discovery rate, Benjamini and Hochberg method^43^]), are reported in eTables 4 to 10 and eTable 12 in Supplement 2. Figure 2, Figure 3, and the eFigure in Supplement 2 show all ROIs with significant *P* values (uncorrected). For deep WM (DWM) ROIs, the number of tests was 42; for superficially located WM (SWM) ROIs, the number of tests was 39; and for GM ROIs of the Multi-Atlas Region Segmentation Utilizing Ensembles template, the number of tests was 120.

## Results

### Study Participants

At baseline, 355 participants (mean [SD] age, 67.7 [8.0] years; 200 men [56.3%] and 155 women [43.7%]) received intensive BP treatment and 315 participants (mean [SD] age, 67.0 [8.4] years; 199 men [63.2%] and 116 women [36.8%]) received standard BP treatment (Table). Information on participants by treatment group is provided in the Table. Additional baseline characteristics between participants in the MRI substudy and remaining trial participants are shown in eTable 2 in Supplement 2. The characteristics of the MRI substudy participants who had both baseline and follow-up scans are provided in eTable 3 in Supplement 2. Group differences are described in eResults 1 in Supplement 2.

**Table.** Characteristics of Participants at Baseline and Follow-up

Variable	Completed baseline scan		Completed follow-up scan	
	Intensive treatment (n = 355)	Standard treatment (n = 315)	Intensive treatment (n = 255)	Standard treatment (n = 203)
Age, mean (SD), y	67.7 (8.0)	67.0 (8.4)	67.9 (7.8)	66.6 (7.9)
Sex, No. (%)				
Male	200 (56.3)	199 (63.2)	154 (60.4)	133 (65.5)
Female	155 (43.7)	116 (36.8)	101 (39.6)	70 (34.5)
Race and ethnicity, No. (%)				
Black	114 (32.1)	104 (33.0)	72 (28.2)	66 (32.5)
Hispanic^a^	14 (3.9)	22 (7.0)	9 (3.5)	13 (6.4)
White	222 (62.5)	184 (58.4)	172 (67.5)	120 (59.1)
Other^b^	5 (1.4)	5 (1.6)	2 (0.8)	4 (2.0)
BP, mean (SD), mm Hg				
Systolic	138.2 (17.6)	137.8 (15.5)	136.0 (16.9)	138.4 (16.1)
Diastolic	77.3 (10.9)	78.5 (12.0)	76.6 (10.7)	79.4 (12.2)
Orthostatic hypotension, No. (%)	26 (7.3)	17 (5.4)	20 (7.8)	15 (7.4)
eGFR, mL/min/1.73 m^2^				
Mean (SD)^c^	72.1 (19.9)	72.3 (21.1)	71.8 (19.3)	73.7 (20.9)
<60, No. (%)	95 (26.8)	91 (28.9)	67 (26.3)	56 (27.6)
Intracranial volume, mean (SD), mm^3^	1373.9 (149.3)	1394.1 (145.0)	1387.3 (141.0)	1398.1 (146.3)
WML volume, mean (SD), mm^3^	3068.2 (5475.3)	2639.9 (4454.4)	3712.6 (6585.1)	3898.0 (6299.7)
DWM DTI-FA, mean (SD)^d^	0.4130 (0.036)	0.4156 (0.028)	0.4104 (0.036)	0.4094 (0.033)
SWM DTI-FA, mean (SD)^d^	0.3387 (0.033)	0.3408 (0.028)	0.3376 (0.036)	0.3368 (0.032)
DWM DTI-MD, mean (SD)^d^	0.8480 (0.310)	0.8318 (0.051)	0.8524 (0.061)	0.8502 (0.057)
SWM DTI-MD, mean (SD)^d^	0.8016 (0.190)	0.7947 (0.045)	0.8054 (0.047)	0.8020 (0.045)
GM CBF, mean (SD), mL/100 mg/min	35.9 (11.9)	35.3 (10.4)	40.5 (15.2)	38.9 (14.1)

Abbreviations: BP, blood pressure; CBF, cerebral blood flow; DTI, diffusion tensor imaging; DWM, deep white matter; eGFR, estimated glomerular filtration rate; FA, fractional anisotropy; GM, gray matter; MD, mean diffusivity; SWM, superficially located white matter; WML, white matter lesion.

^a^ Encompasses a self-report of being of Spanish, Hispanic, or Latino origin, independent of any other race and ethnicity designation.

^b^ Includes Asian, American Indian or Alaskan Native, Native Hawaiian or Pacific Islander, or other races.

^c^ Based on Modification of Diet in Renal Disease Study equation.

^d^ Scalar values.

### White Matter Lesions

Overall, intensive treatment was associated with smaller mean increases in WML volume compared with standard treatment (644.5 mm^3^ vs 1258.1 mm^3^; Table). Specifically, the smaller increases were observed in the DWM regions, such as the left anterior corona radiata (intensive treatment, 30.3 mm^3^ [95% CI, 16.0-44.5 mm^3^]; standard treatment, 80.5 mm^3^ [95% CI, 53.8-107.2 mm^3^]; *P* = .003), left tapetum (intensive treatment, 11.8 mm^3^ [95% CI, 4.4-19.2 mm^3^]; standard treatment, 27.2 mm^3^ [95% CI, 19.4-35.0 mm^3^]; *P* = .046), left superior fronto-occipital fasciculus (intensive treatment, 3.2 mm^3^ [95% CI, 0.7-5.8 mm^3^]; standard treatment, 9.4 mm^3^ [95% CI, 5.5-13.4 mm^3^]; *P* = .046), left posterior corona radiata (intensive treatment, 26.0 mm^3^ [95% CI, 12.9-39.1 mm^3^]; standard treatment, 52.3 mm^3^ [95% CI, 34.8-69.8 mm^3^]; *P* = .046), left splenium of the corpus callosum (intensive treatment, 45.4 mm^3^ [95% CI, 25.1-65.7 mm^3^]; standard treatment, 83.0 mm^3^ [95% CI, 58.7-107.2 mm^3^]; *P* = .046), left posterior thalamic radiation (intensive treatment, 53.0 mm^3^ [95% CI, 29.8-76.2 mm^3^]; standard treatment, 106.9 mm^3^ [95% CI, 73.4-140.3 mm^3^]; *P* = .046), and right posterior thalamic radiation (intensive treatment, 49.5 mm^3^ [95% CI, 24.3-74.7 mm^3^]; standard treatment, 102.6 mm^3^ [95% CI, 71.0-134.2 mm^3^]; *P* = .047) (Figure 2A). Smaller increases in WML volume were associated with intensive treatment in the right body of the corpus callosum, right splenium, right anterior corona radiata, right posterior corona radiata, left anterior limb of the internal capsule, and right tapetum (Figure 2A). For all WM ROIs, the effect sizes and changes per group are reported in eTable 4 and eTable 5 in Supplement 2. Changes in SWM ROIs for WMLs are described in eResults 2 in Supplement 2.

### DTI: Fractional Anisotropy and Mean Diffusivity

Overall, we noted that the FA was associated with a slower decrease over time with intensive treatment (mean change, −0.0026 for DWM ROIs and −0.0011 for SWM ROIs) compared with standard treatment (mean change, −0.0062 for DWM ROIs and −0.0040 for SWM ROIs), as shown in the Table. Deep WM regions, such as the left posterior thalamic radiation (intensive treatment, –0.0003 [95% CI, –0.0052 to 0.0047]; standard treatment, –0.0049 [–0.0105 to 0.0005]; uncorrected *P* = .01), left superior fronto-occipital fasciculus (intensive treatment, 0.0108 [95% CI, 0.0043-0.0173]; standard treatment 0.0002 [95% CI, –0.0055 to 0.0059]; uncorrected *P* = .02), left anterior limb of the internal capsule (intensive treatment, 0.0061 [95% CI, 0.0013-0.0109]; standard treatment, –0.0005 [95% CI, –0.0053 to 0.0043]; uncorrected *P* = .03), left tapetum (intensive treatment, 0.0025 [95% CI, –0.0045 to 0.0096]; standard treatment, –0.0041 [95% CI, –0.0126 to 0.0045]; uncorrected *P* = .04), left sagittal stratum (intensive treatment, –0.0046 [95% CI, –0.0089 to –0.0004]; standard treatment, –0.0098 [95% CI, –0.0146 to –0.005]; uncorrected *P* = .047), and right anterior corona radiata (intensive treatment, –0.0013 [95% CI, –0.0044 to 0.0017]; standard treatment, –0.0048 [95% CI, –0.0079 to –0.0017]; uncorrected *P* = .047), experienced slower decreases in FA associated with intensive treatment (Figure 2B).

Intensive treatment was associated with an overall smaller mean increase in MD compared with standard treatment (0.0044 vs 0.0184 for DWM ROIs and 0.0038 vs 0.0073 for SWM ROIs [Table]). Specifically, smaller increases were observed in the left superior fronto-occipital fasciculus (intensive treatment, 0.0158 [95% CI, 0.0042-0.0275]; standard treatment, 0.0317 [95% CI, 0.0219-0.0414]; uncorrected *P* = .03), left posterior thalamic radiation (intensive treatment, 0.0154 [95% CI, 0.0086-0.0221]; standard treatment, 0.0226 [95% CI, 0.0151-0.0301]; uncorrected *P* = .04), and left tapetum (intensive treatment, 0.0335 [95% CI, 0.0179-0.0491]; standard treatment, 0.0533 [95% CI, 0.0354-0.0711]; uncorrected *P* = .047) (Figure 2C). eTables 6 to 9 in Supplement 2 report the effect size, mean change per ROI, mean MD at baseline, and mean MD at follow-up per treatment group for all the WM ROIs. Changes in SWM ROIs for FA and MD are described in eResults 2 in Supplement 2.

### Cerebral Blood Flow

Overall, there was a larger increase in mean CBF associated with intensive treatment compared with standard treatment (4.6 mL/100 mg/min vs 3.7 mL/100 mg/min; Table). Specifically, larger increases in CBF with intensive treatment were noted in in the right middle occipital gyrus (intensive treatment, 8.8 mL/100 mg/min [95% CI, 5.5-12.2 mL/100 mg/min]; standard treatment, 1.1 mL/100 mg/min [95% CI, –3.7 to 6.0 mL/100 mg/min]; uncorrected *P* = .004), left cuneus (intensive treatment, 6.2 mL/100 mg/min [95% CI, 3.8-8.6 mL/100 mg/min]; standard treatment, 3.1 mL/100 mg/min [95% CI, 0.4-5.7 mL/100 mg/min]; uncorrected *P* = .02), left superior occipital gyrus (intensive treatment, 6.5 mL/100 mg/min [95% CI, 3.7-9.4 mL/100 mg/min]; standard treatment, 0.7 mL/100 mg/min [95% CI, –4.0 to 5.4 mL/100 mg/min]; uncorrected *P* = .02), right inferior occipital gyrus (intensive treatment, 7.1 mL/100 mg/min [95% CI, 4.1-10.1 mL/100 mg/min]; standard treatment, 2.4 mL/100 mg/min [95% CI, –0.9 to 5.8 mL/100 mg/min]; uncorrected *P* = .03), right cuneus (intensive treatment, 6.0 mL/100 mg/min [95% CI, 3.7-8.4 mL/100 mg/min]; standard treatment, 2.9 mL/100 mg/min [95% CI, 0.0-5.7 mL/100 mg/min]; uncorrected *P* = .03), left inferior occipital gyrus (intensive treatment, 8.0 mL/100 mg/min [95% CI, 4.9-11.2 mL/100 mg/min]; standard treatment, 2.6 mL/100 mg/min [95% CI, –1.5 to 6.8 mL/100 mg/min]; uncorrected *P* = .03), right occipital pole (intensive treatment, 9.5 mL/100 mg/min [95% CI, 5.4-13.6 mL/100 mg/min]; standard treatment, 2.4 mL/100 mg/min [95% CI, –2.9 to 7.7 mL/100 mg/min]; uncorrected *P* = .03), right superior occipital gyrus (intensive treatment, 5.5 mL/100 mg/min [95% CI, 2.6-8.4 mL/100 mg/min]; standard treatment, 0.1 mL/100 mg/min [95% CI, –4.4 to 4.6 mL/100 mg/min]; uncorrected *P* = .04), right middle frontal gyrus (intensive treatment, 1.3 mL/100 mg/min [95% CI, –1.0 to 3.5 mL/100 mg/min]; standard treatment, –1.6 mL/100 mg/min [95% CI, –3.9 to 0.8 mL/100 mg/min]; uncorrected *P* = .04), left superior parietal lobule (intensive treatment, 1.1 mL/100 mg/min [95% CI, –0.9 to 3.0 mL/100 mg/min]; standard treatment, –2.2 mL/100 mg/min [95% CI, –5.2 to 0.8 mL/100 mg/min]; uncorrected *P* = .045), left precentral gyrus (intensive treatment, 1.8 mL/100 mg/min [95% CI, –0.3 to 4.0 mL/100 mg/min]; standard treatment, –0.9 mL/100 mg/min [95% CI, –3.4 to 1.7 mL/100 mg/min]; uncorrected *P* = .046), and right supramarginal gyrus (intensive treatment, 5.9 mL/100 mg/min [95% CI, 3.1-8.6 mL/100 mg/min]; standard treatment, 2.9 mL/100 mg/min [95% CI, –0.2 to 5.9 mL/100 mg/min]; uncorrected *P* = .049) (Figure 3). eTable 10 in Supplement 2 reports the effect size, mean change per group, mean CBF at baseline, and mean CBF at follow-up for all GM ROIs.

### Sensitivity Analysis

We performed multiple imputation (eMethods 2 in Supplement 2) to account for bias due to missing follow-up data (eTable 11 in Supplement 2). The results showed an association between intensive treatment and reduced progression of WMLs in the left anterior corona radiata, left tapetum, left superior fronto-occipital fasciculus, left posterior corona radiata, left splenium of the corpus callosum, both sides of the posterior thalamic radiation, right body of the corpus callosum, right splenium, right anterior corona radiata, right posterior corona radiata, left anterior limb of the internal capsule, and right tapetum (eTable 12 in Supplement 2).

## Discussion

Intensive SBP treatment was associated with reduced progression of WMLs in the anterior and posterior corona radiata, tapetum, superior fronto-occipital fasciculus, and the corpus callosum in this post hoc substudy of the SPRINT MIND clinical trial. Further analyses indicated a slower decrease in FA, a slower increase in MD, and an improvement in CBF associated with intensive treatment. Overall, our results were consistent with prior findings in other studies. Reduction of WML progression due to BP treatment has been reported previously in the INFINITY (Intensive Versus Standard Ambulatory Blood Pressure Lowering to Prevent Functional Decline In the Elderly) trial,^44^ PRESERVE (How Intensively Should We Treat Blood Pressure in Established Cerebral Small Vessel Disease) trial,^45^ PROGRESS (Perindopril Protection Against Recurrent Stroke Study),^46^ ACCORD-MIND (Action to Control Cardiovascular Risk in Diabetes Memory in Diabetes) study,^47^ and others.^48,49^ These studies treated WML as a scored lesion load across the whole brain,^45,48,49^ total volume,^44,47^ or both.^46^ To our knowledge, this is the first study with a large-scale longitudinal cohort examining the regional associations of BP treatment with WMLs, DTI measures, and CBF using data from a randomized double-blind clinical trial targeting hypertension treatment.

### WML Volume Changes With Strict BP Control

This study suggests that intensive BP treatment was associated with a slowing of WML increase in the DWM of the corona radiata and thalamic radiations, along with some of the commissural fibers in the corpus callosum. Our analysis points to the beneficial associations of intensive BP treatment (specifically, injury patterns related to dementia). A previous cross-sectional study^50^ reported decreased WML volume in the corpus callosum and anterior or posterior corona radiata among patients with vascular dementia and patients with mild cognitive impairment of vascular origins. Our study suggests that intensive BP treatment is associated with reduced WM damage and the prevention of WMLs in these tracts, which in turn could preserve myelin structures along these tracts and ultimately slow the progression of injury patterns associated with dementia.

Superficially located WM lesion progression differences were found between the 2 groups (eFigure, A, and eTable 2 in Supplement 2). The SWM lesions were neighboring the DWM regions and, similar to the DWM findings, had a pattern of reduced WML progression over time associated with intensive treatment compared with standard treatment (eFigure, A, in Supplement 2). Superficially located WM has less myelination and tends to be more vulnerable to pathologic damage.^51,52^

### DTI Changes With Strict BP Control

Fractional anisotropy is indicative of the direction of the diffusion of water molecules, while MD describes the rotationally invariant magnitude of the diffusion. Higher FA values are typically observed in heavily myelinated WM tracts,^53^ and lower values have been associated with pathologic findings. The Multi-Ethnic Study of Atherosclerosis, using a comparable DTI processing pipeline, indicated that global FA decreases by approximately 0.26 per 5 years in the population in the eighth decade of life.^33^ However, reports on regional DTI measures in the population are scarce, and reference data are therefore less available, to our knowledge.

Changes in FA and, to a lesser extent, MD, generally aligned with WML findings, with strict BP control associated with smaller decreases in FA and smaller increases in MD over time with intensive treatment. The PRESERVE study^45^ did not find any associations between BP treatment and DTI measures. As reported in a previous substudy,^27^ we found no significant differences in mean FA for any parts of the corpus callosum or cingulum. This finding may be due to the greater interindividual variability of FA in the corpus callosum,^54^ which makes it more difficult to properly assess the structure accurately from a registration perspective despite good reproducibility of results longitudinally. However, it cannot be ruled out that there may be an underlying difference in the WM microstructure (due to differing pathologic damage in the commissural fibers compared with nearby projection fibers) that was associated with the difference in FA, which is worth future investigation. The same study^54^ also found similar higher reproducibility with lower reliability in MD to an even greater extent compared with FA, which may explain the lack of significant differences found in MD between the 2 treatment groups.

### CBF Changes With Strict BP Control

When gross measures such as global GM CBF were assessed,^28^ no significant differences were found between the 2 treatment groups. Our regional analysis, on the other hand, could have been more sensitive, as individual regions have inconsistent directions associated with intensive BP control (increase vs decrease), and therefore had an attenuated overall outcome. Nonetheless, significant unadjusted *P* values were associated with intensive treatment in all instances. Our study showed that intensive BP control was associated with increased blood perfusion, especially in the occipital lobe. Previous cross-sectional studies^55^ showed decreased occipital perfusion for participants with hypertension. SPRINT had a lower BP goal (<120 mm Hg) than previous observational longitudinal studies of BP control.^48,56,57^ This finding suggests that even with more aggressive BP goals, cerebrovascular autoregulatory mechanisms will compensate for the decreased systemic BP, preventing hypoperfusion and leading to improvements in regional CBF. These changes likely take place at a capillary level.^58^ In our study, CBF improvement was mainly localized to posterior cerebral circulation. Although the cause of this topographical preference is unclear, one hypothesis is that posterior and anterior cerebral circulation may have different regulatory mechanisms for blood flow that make the posterior circulation more vulnerable to vascular dysfunction, which is improved with strict BP control back to a nonhypertensive state.^59,60^ Studies investigating this hypothesis have produced mixed findings on whether there is diminished autoregulatory capacity in the posterior circulation^61,62,63^ or not.^64,65^ Previous studies have demonstrated relatively confined regions of decreased cerebral blood flow as a result of hypertension. Among patients with treated hypertension, there are still areas of hypoperfusion in the left parietal and right frontorolandic regions compared with controls^66^ (as opposed to global cerebral hypoperfusion in patients with untreated hypertension).

Another study showed that participants with hypertension showed reduced temporal and occipital perfusion compared with those without hypertension, regardless of antihypertensive treatment status.^55^ Although it is difficult to hypothesize the exact biological cause of the specific regional differences due to varying study designs, we believe that this finding nevertheless demonstrates that regional perfusion differences may emerge with hypertension or hypertension control that may not be reflected in global perfusion comparisons after being averaged out. In the previous analysis of the secondary outcomes, there was a small increase in GM CBF associated with intensive treatment compared with standard treatment that did not reach statistical significance, likely owing in part to these regional differences.

A limitation is that these studies have been focused on compensatory mechanisms to more abrupt changes as opposed to long-term differences over the course of years. Nevertheless, the findings in this substudy of potential posterior-anterior circulation differences may merit further investigation.

### Limitations

There are some limitations in this study. First is the 4-year duration between the start of intervention and follow-up. Second is the fact that fewer participants completed follow-up MRI scans, partially due to early termination of the trial. Third is that the reported DTI and CBF measures did not show significance after multiple comparisons (possibly due to a higher noise-to-signal ratio in DTI and CBF compared with fluid-attenuated inversion recovery), and their interpretations should be considered exploratory. Increasing the sample size in future clinical trials would help in reaching statistical significance after correction for multiple comparisons for regional analyses in those modalities.

## Conclusions

In this post hoc secondary analysis of a randomized clinical trial of SBP control, intensive treatment, compared with standard treatment, of hypertension was associated with decreased progression of WMLs, specifically in the left anterior corona radiata, left tapetum, left superior fronto-occipital fasciculus, posterior thalamic radiations, left posterior corona radiata, and left splenium of the corpus callosum. Corresponding changes in mean FA (in overlapping regions, mainly the left posterior thalamic radiation, left superior fronto-occipital fasciculus, left anterior limb of internal capsule, left tapetum, and right anterior corona radiata) and MD (mainly in the left superior fronto-occipital fasciculus, left posterior thalamic radiation, and left tapetum) had a marginal association among other regions, suggesting the vulnerability of those regions to hypertension. Intensive SBP control was also associated with improved posterior cerebral perfusion. Our findings support the hypothesis that intensive BP treatment has beneficial associations with small vessel disease biomarkers. Specific areas show greater benefit, representing sensitive regions to track in future trials evaluating small vessel disease.

## Notes

Supplement 1.
Trial Protocol

Supplement 2. **eTable 1.** SPRINT MRI Scanner Parameters
eMethods 1.

eResults 1.
**eTable 2.** Baseline Characteristics of Participants in MRI Substudy vs Remaining Trial Participants **eTable 3.** Characteristics of Participants in MRI Substudy Who Had Both Baseline and Follow-up Scans **eResults 2.** Superficially Located WM (SWM) **eFigure.** Patterns of Changes in the Type III White Matter Parcellation Map (WMPM) Derived Deep White Matter Regions-of-Interest (ROIs) When Comparing Intensive Treatment Group With Standard Treatment Group **eTable 4.** Changes in White Matter Lesions (WML) Derived in Deep White Matter (DWM) Regions-of-Interest (ROIs) Between Intensive Treatment Group and Standard Treatment Group **eTable 5.** Changes in White Matter Lesions (WML) Derived in Superficially-Located White Matter (SWM) Regions-of-Interest (ROIs) Between Intensive Treatment Group and Standard Treatment Group **eTable 6.** Changes in Diffusion Tensor Imaging (DTI) Fractional Anisotropy (FA) Derived in Deep White Matter (DWM) Regions-of-Interest (ROIs) Between Intensive Treatment Group and Standard Treatment Group **eTable 7.** Changes in Diffusion Tensor Imaging (DTI) Fractional Anisotropy (FA) Derived in Superficially-Located White Matter (SWM) Regions-of-Interest (ROIs) Between Intensive Treatment Group and Standard Treatment Group **eTable 8.** Changes in Diffusion Tensor Imaging (DTI) Mean Diffusivity (MD) Derived in Deep White Matter (DWM) Regions-of-Interest (ROIs) Between Intensive Treatment Group and Standard Treatment Group **eTable 9.** Changes in Diffusion Tensor Imaging (DTI) Mean Diffusivity (MD) Derived in Superficially-Located White Matter (SWM) Regions-of-Interest (ROIs) Between Intensive Treatment Group and Standard Treatment Group **eTable 10.** Changes in Cerebral Blood Flow (CBF) Derived in Gray Matter (GM) Regions-of-Interest (ROIs) Between Intensive Treatment Group and Standard Treatment Group **eMethods 2.** Multiple Imputation **eTable 11.** Condition Model for Multiple Imputation **eTable 12.** Changes in White Matter Lesions (WML) Derived in Deep White Matter (DWM) Regions-of-Interest (ROIs) Between Intensive Treatment Group and Standard Treatment Group After Multiple Imputation
eReferences.

Supplement 3.
Data Sharing Statement
