# Supplementary material for: Association of Intensive vs Standard Blood Pressure Control With Regional Changes in Cerebral Small Vessel Disease Biomarkers: Post Hoc Secondary Analysis of the SPRINT MIND Randomized Clinical Trial
Source: JAMA Netw Open. 2023 Mar 1;6(3):e231055. doi: 10.1001/jamanetworkopen.2023.1055 (PMC9978954; doi:10.1001/jamanetworkopen.2023.1055)
Supplement: Supplement 2. — eTable 1. SPRINT MRI Scanner Parameters eMethods 1. eResults 1. eTable 2. Baseline Characteristics of Participants in MRI Substudy vs Remaining Trial Participants eTable 3. Characteristics of Participants in MRI Substudy Who Had Both Baseline and Follow-up Scans eResults 2. Superficially Located WM (SWM) eFigure. Patterns of Changes in the Type III White Matter Parcellation Map (WMPM) Derived Deep White Matter Regions-of-Interest (ROIs) When Comparing Intensive Treatment Group With Standard Treatment Group eTable 4. Changes in White Matter Lesions (WML) Derived in Deep White Matter (DWM) Regions-of-Interest (ROIs) Between Intensive Treatment Group and Standard Treatment Group eTable 5. Changes in White Matter Lesions (WML) Derived in Superficially-Located White Matter (SWM) Regions-of-Interest (ROIs) Between Intensive Treatment Group and Standard Treatment Group eTable 6. Changes in Diffusion Tensor Imaging (DTI) Fractional Anisotropy (FA) Derived in Deep White Matter (DWM) Regions-of-Interest (ROIs) Between Intensive Treatment Group and Standard Treatment Group eTable 7. Changes in Diffusion Tensor Imaging (DTI) Fractional Anisotropy (FA) Derived in Superficially-Located White Matter (SWM) Regions-of-Interest (ROIs) Between Intensive Treatment Group and Standard Treatment Group eTable 8. Changes in Diffusion Tensor Imaging (DTI) Mean Diffusivity (MD) Derived in Deep White Matter (DWM) Regions-of-Interest (ROIs) Between Intensive Treatment Group and Standard Treatment Group eTable 9. Changes in Diffusion Tensor Imaging (DTI) Mean Diffusivity (MD) Derived in Superficially-Located White Matter (SWM) Regions-of-Interest (ROIs) Between Intensive Treatment Group and Standard Treatment Group eTable 10. Changes in Cerebral Blood Flow (CBF) Derived in Gray Matter (GM) Regions-of-Interest (ROIs) Between Intensive Treatment Group and Standard Treatment Group eMethods 2. Multiple Imputation eTable 11. Condition Model for Multiple Imputation eTable 12. Changes in White Matter L [file jamanetwopen-e231055-s002.pdf]

Supplementary Online Content

Rashid T, Li K, Toledo JB, et al. Association of intensive vs standard blood pressure control with regional changes in cerebral small vessel disease biomarkers: post hoc secondary analysis of the SPRINT MIND randomized clinical trial. *JAMA Netw Open.* 2023;6(3):e231055. doi:10.1001/jamanetworkopen.2023.1055

eTable 1. SPRINT MRI Scanner Parameters
eMethods 1.
eResults 1.
eTable 2. Baseline Characteristics of Participants in MRI Substudy vs Remaining Trial Participants
eTable 3. Characteristics of Participants in MRI Substudy Who Had Both Baseline and Follow-up Scans
eResults 2. Superficially Located WM (SWM)
eFigure. Patterns of Changes in the Type III White Matter Parcellation Map (WMPM) Derived Deep White Matter Regions-of-Interest (ROIs) When Comparing Intensive Treatment Group With Standard Treatment Group
eTable 4. Changes in White Matter Lesions (WML) Derived in Deep White Matter (DWM) Regions-of-Interest (ROIs) Between Intensive Treatment Group and Standard Treatment Group
eTable 5. Changes in White Matter Lesions (WML) Derived in Superficially-Located White Matter (SWM) Regions-of-Interest (ROIs) Between Intensive Treatment Group and Standard Treatment Group
eTable 6. Changes in Diffusion Tensor Imaging (DTI) Fractional Anisotropy (FA) Derived in Deep White Matter (DWM) Regions-of-Interest (ROIs) Between Intensive Treatment Group and Standard Treatment Group
eTable 7. Changes in Diffusion Tensor Imaging (DTI) Fractional Anisotropy (FA) Derived in Superficially-Located White Matter (SWM) Regions-of-Interest (ROIs) Between Intensive Treatment Group and Standard Treatment Group
eTable 8. Changes in Diffusion Tensor Imaging (DTI) Mean Diffusivity (MD) Derived in Deep White Matter (DWM) Regions-of-Interest (ROIs) Between Intensive Treatment Group and Standard Treatment Group
eTable 9. Changes in Diffusion Tensor Imaging (DTI) Mean Diffusivity (MD) Derived in Superficially-Located White Matter (SWM) Regions-of-Interest (ROIs) Between Intensive Treatment Group and Standard Treatment Group
eTable 10. Changes in Cerebral Blood Flow (CBF) Derived in Gray Matter (GM) Regions-of-Interest (ROIs) Between Intensive Treatment Group and Standard Treatment Group
eMethods 2. Multiple Imputation
eTable 11. Condition Model for Multiple Imputation
eTable 12. Changes in White Matter Lesions (WML) Derived in Deep White Matter (DWM) Regions-of-Interest (ROIs) Between Intensive Treatment Group and Standard Treatment Group After Multiple Imputation
eReferences.

This supplementary material has been provided by the authors to give readers additional information about their work.

© 2023 Rashid T et al. *JAMA Network Open.*

eTable 1. SPRINT MRI Scanner Parameters

	TR (ms)	TE (ms)	FOV (mm)	Slice Thickness (mm)	No of Slices	Matrix	Scan Duration
**T1w**	1900	2.89	250	1	176	256 x 256	4 min, 26 sec
**T2w**	3200	409	250	1	176	258 x 256	4 min, 8 sec
**FLAIR**	6000 (TI=2200 ms)	160	250	1	160	258 x 221	8 min, 20 sec
**DTI**	7300	84	245	2.2	64	128 x 128	4 min, 37 sec
**CBF**	4000	11	220	5	20	64 x 64	5 min, 32 sec

© 2023 Rashid T et al. *JAMA Network Open.*

eMethods 1.

Trial Design

While the study originally intended to have an average follow-up of 5 years, the study was terminated in 2015 due to evidence of significantly improved primary outcome (reduction in composite cardiovascular disease outcomes).

The targeted sample size was set based on the Antihypertensive and Lipid-Lower Treatment to Prevent Heart Attack Trial (ALLHAT), with a target sample size of 9,250 participants expected to provide 90% power to detect a 20% effect in the primary outcome. With 640 participants (320 participants in each treatment group), after accounting for a 3% per year loss to follow-up, the estimated power to detect group difference in WML volume of 0.65 cm^3 is 80% and 90% for WML volume of 0.76 cm^3 over a period of 4 years (Supplementary Materials 1). Participants were required to be 50 years or older with SBP between 130- and 180 mm Hg at the screening visit and at increased cardiovascular risk defined as having clinical or subclinical cardiovascular disease; chronic kidney disease (defined as an estimated glomerular filtration rate of less than 60 ml/min/1.73 m^2; a Framingham Risk Score for 10-year cardiovascular disease risk of 15% or more; or age of 75 years or older. Exclusion criteria included diabetes mellitus, a history of stroke, dementia diagnosis, and presence of implanted electrical devices such as a pacemaker, neurostimulator or defibrillator. More details on SPRINT protocol[1, 2] including inclusion and exclusion criteria can be found in Supplementary Materials 1.

Study Participants

The medications used in both groups were a combination of a thiazide-type diuretic (chlorthaliadone preferred), an ACE inhibitor or angiotensin receptor blocker (never both), a calcium channel blocker (amlodipine preferred), and/or a beta-blocker titrated as necessary to dosages effective for achieving SBP goal. Visits occurred every three months to reassess blood pressure to ensure participants continued to remain on-target, with titration of medication done as necessary if SBP is too high. For the intensive therapy group, if the participant is above the goal SBP of 120 mm Hg, they would have their medications adjusted and seen on a monthly basis until SBP is below 120 mm Hg. As there are clear targets for blood pressure, neither providers nor participants were blinded to the treatment arm. However, all adjudicators involved in classification of primary and secondary outcomes in the main study as well as all substudies were blinded to the treatment assignment.

The SPRINT Memory and cognition IN Decreased hypertension (SPRINT-MIND) sub-study aimed to determine whether an intensive SBP treatment strategy would have an effect on risk of dementia. Using a subgroup of approximately 2,800 participants with 640 participants would provide an 80% to detect a 15% reduction in incidence of dementia and 80 and 90% power in detecting differences in small vessel ischemic disease and total brain volume. The hypothesized primary outcome with intensive SBP control is a reduction in the incidence of all-cause dementia compared to the standard strategy, with hypothesized secondary outcomes of less decline of cognition in specific domains, and reduced pathologic brain changes as derived from MRI.

© 2023 Rashid T et al. *JAMA Network Open.*

Analytical Sample

Of the 639 participants in the intensive treatment group, 227 participants were excluded (124 were unwilling to participate and 103 were ineligible due to the presence of metallic foreign bodies or exposure to metal fragments; severe claustrophobia; presence of pacemaker, defibrillator, neurostimulator, or other implanted electrical device; the presence of cerebral aneurysm clip, cochlear or other otologic implants). Of the remaining 412 participants in the intensive treatment group, 355 participants completed baseline MRI scans, with 106 participants missing follow-up MRI scans due to unwillingness to participate, withdrawal of consent, death or ineligibility, or giving. Two hundred fifty-five participants with follow-up scans. Of the 628 participants in the standard treatment group, 247 participants were excluded (145 participants unwilling to participate and 102 ineligible). Of the remaining 381 participants, 315 completed baseline MRI scans, with 115 participants excluded from follow-up scans due to ineligibility, withdrawal of consent, death, or lack of follow-up MRI scans, giving a total of 203 participants with follow-up MRI.

MRI

The SPRINT MIND study acquired brain MRI scans of participants at baseline and 48-month follow-up after randomization. Scans were conducted at seven sites, with different scanner models: 3T Phillips Achieva 3.2 at the University of Alabama at Birmingham, Boston University, and Vanderbilt University; 3T Siemens Skyra VD11B at Wake Forest University, 3T Siemens Tim Trio VB17 at the University of Miami and University of Pennsylvania and 3T Siemens Verio VB17 at Case Western University. Scans included T1-weighted, T2-weighted, fluid-attenuated inversion recovery (FLAIR), pseudo-continuous arterial spin labeling (PCASL), and diffusion tensor imaging (DTI). Imaging parameters are detailed in eTable 1.

Scanner performance was monitored with quarterly quality checks using the Alzheimer Disease Neuroimaging Initiative (ADNI) and Function Biomedical Informatics Research Network (FBIRN) phantom scans, with all scanners showing stability of phantom measurements throughout the trial.

Duration of Follow-up

On August 20, 2015, the process to end the intervention trial early was initiated, based on a recommendation by the data and safety monitoring board to inform the investigators and participants of the cardiovascular outcome results. As a result, the majority of the follow-up MRI scans used in this study (n=458) occurred during the closeout period (from August 20, 2015, to July 1, 2016) when the participants were transitioning to having their hypertensive treatment managed by their primary care clinician, although antihypertensive drugs were still provided by the study.

Cerebral Blood Flow

CBF maps were derived using a custom data cleaning and processing pipeline[3] from pseudo-continuous arterial spin labeling (PCASL) MRI, with recommendations of Alsop et al.[4]. The processing pipeline consisted of motion correction, CBF quantification, and denoising based on structural correlation with Robust Bayesian criteria[5, 6]. The quality of the CBF maps was evaluated by an automated method[7], which

© 2023 Rashid T et al. *JAMA Network Open.*

generates an index between 0 and 1. Gray matter ROIs were derived from T1-weighted MRI using the Multi-atlas region Segmentation utilizing Ensembles (MUSE) method[8]. The T1w images were registered to CBF images, and lastly, the mean CBF for the GM ROIs defined in MUSE atlas[8] were calculated.

White Matter Lesions

WMLs were calculated from participants' fluid-attenuated inversion recovery (FLAIR) images. The FLAIR and T1-weighted images were first corrected for inhomogeneity[9] and then the corrected FLAIR images were co-registered to their respective T1-weighted images. WMLs were segmented from corrected FLAIR images using a deep learning method[10] based on a variant of the popular 2D U-Net architecture[11]. We calculated the WML volume of deep white matter (DWM) and superficially located white matter (SWM) ROIs using the Type III white matter parcellation map (WMPM-III) defined in Oishi et al[12]. First, the participants' WML segmentations (in FLAIR space) were rigidly transformed into T1 space. Then the participants' T1-weighted images were non-rigidly registered to the Type III WMPM template using the Advanced Normalization Tool[13] (ANTs). Finally, the deformation field was applied to the transformed WML segmentation maps. The WML segmentation maps were quality inspected by a neuroradiologist blinded to the treatment group.

DTI – Fractional Anisotropy and Mean Diffusivity

We derived the fractional anisotropy (FA) and mean diffusivity (MD) for both baseline and follow-up participants using a DTI processing pipeline[14]. Briefly, the 4D diffusion MRI underwent correction for eddy current distortion and head movements[15, 16], followed by brain extraction[17] and Gaussian smoothing. The eigenvalues and subsequent FA and MD metrics were calculated from the corrected diffusion MRI using functions and utilities from a publicly available toolbox[18].

The mean FA and MD metrics were calculated for each WM ROI defined in the Type III WMPM template. First, the Type III WMPM T1-weighted image was non-rigidly registered to participants' T1-weighted images using ANTs[13], and the deformation field was applied to the Type III WMPM template. Then, the participants' T1-weighted image was rigidly registered to DTI space, and the corresponding affine transform was applied to the already transformed Type III WMPM template, thus bringing the Type III WMPM template into the DTI space.

© 2023 Rashid T et al. *JAMA Network Open.*

eResults 1.

Study Participants

The demographic characteristics of the 670 participants who had completed a baseline MRI scan did not differ between treatment groups. For the standard treatment group at baseline, 315 participants had a mean age of 66.96 years, 36.8% were women and 58.4% were non-Hispanic White. In the intensive treatment group, 355 participants had completed baseline scans with a mean age of 67.63 (SD, 7.96), and 43.7% were women and 62.5% were non-Hispanic White. Two hundred fifty-five participants in the intensive treatment group with completed follow-up scans had a mean age of 67.65 (SD, 7.74), 39.6% were women, and 67.5% were non-Hispanic White. Two hundred and three participants in the standard treatment group with completed follow-up scans had a mean age of 66.56 years (SD, 7.90), 34.5% were women and 59.1% were non-Hispanic White. Compared to participants who are not enrolled in the MRI substudy, participants in the MRI substudy were more likely to have lower SPB at baseline and less likely to have a history of cardiovascular disease (eTable 2). When comparing participants who had both baseline and follow-up scans, the participants in the intensive treatment group were more likely to be slightly older, and had lower diastolic BP (eTable 3).

Sustained between-group difference in SBP was reported previously[19]. The intensive treatment group had a mean SBP of 120.7 mm Hg and the standard treatment group had a mean SBP of 134.9 mm Hg (difference of 14.2 mm Hg, 95% CI, 13.1 to 15.3 mm Hg). During the transitional closeout period when most of the follow-up MRI scans occurred, the mean SBP for the intensive treatment group increased to 122.1 mm Hg and the standard treatment group had an increase of 136.1 mm Hg (between group difference of 14.0 mm Hg, 95% CI, 9.3 to 18.8 mm Hg).

© 2023 Rashid T et al. *JAMA Network Open.*

eTable 2. Baseline Characteristics of Participants in MRI Substudy vs Remaining Trial Participants

Variable	In MRI Substudy N=670	Not in MRI Substudy N=8688[a]	P-value
**Randomized to Intensive treatment, No. (%)**	355 (53.0)	4322 (49.7)	0.12
**Age, mean (SD), years**	67.3 (8.2)	68.0 (9.5)	0.08
**Age 75 year or older, No. (%)**	150 (22.4)	2486 (28.6)	0.001
**Sex, No. (%)**			0.007
**Male**			
**Female**	271 (40.4)	3061 (35.2)	
**Race/Ethnicity, No. (%)**			< 0.001
**White**	406 (60.6)	4990 (57.4)	
**Black**	218 (32.5)	2584 (29.7)	
**Hispanic[b]**	36 (5.4)	948 (10.9)	
**Other[c]**	10 (1.5)	166 (1.9)	
**Education, No. (%)**			0.39
**Less than high school**	53 (7.9)	823 (9.5)	
**High school graduate**	103 (15.4)	1433 (16.5)	
**Post high school training**	239 (35.7)	3072 (35.4)	
**College graduate or greater**	275 (41.0)	3360 (38.7)	
**Smoking status, No. (%)**			0.94
**Never smoker**	301 (44.9)	3846 (44.3)	
**Former smoker**	282 (42.1)	3690 (42.5)	
**Current smoker**	87 (13.0)	1152 (13.3)	
**Polypharmacy, No. (%)**			0.32
**< 5 medications**	289 (43.1)	3783 (43.5)	
**5 to < 10 medications**	274 (40.9)	3696 (42.5)	
**10 or more medications**	107 (16.0)	1209 (13.9)	
**Body Mass Index, mean (SD)m kg/m^2**	29.8 (5.4)	29.9 (5.8)	0.70
**History of CVD, No. (%)**	93 (13.9)	1784 (20.5)	< 0.001
**Systolic BP, mean (SD), mm Hg**	138.0 (16.6)	139.8 (15.5)	0.004
**Diastolic BP, mean (SD), mm Hg**	77.9 (11.4)	78.1 (12.0)	0.54
**Orthostatic hypotension, No. (%)[d]**	43 (6.4)	642 (7.4)	0.39
**eGFR, mean (SD), ml/min/1.73m^2 [e]**	72.3 (20.6)	71.7 (20.6)	0.50
**eGFR<60 ml/min/1.72m^2, No. (%)[e]**	184 (27.5)	2461 (28.5)	0.63

[a] Excludes 3 participants without a measured white matter lesion volume at baseline, but who completed baseline MRI scan with a measurement of total brain volume that passed quality control

[b] Hispanic race/ethnicity encompasses a self-report of being Spanish, Hispanic or Latino origin, independent of any other race/ethnicity designation.

[c] Other race/ethnicity includes categories of Asian, American Indian/Native Alaskan, Native Hawaiian/Pacific Islander, or other.

[d] Defined as a standing systolic BP minus seated systolic BP ≤ -20 mm Hg or a standing diastolic BP minus seated diastolic BP ≤ -10 mm Hg.

[e] Based on the 4-variable Modification of Diet in Renal Disease equation.

© 2023 Rashid T et al. *JAMA Network Open.*

eTable 3. Characteristics of Participants in MRI Substudy Who Had Both Baseline and Follow-up Scans

Variables	Intensive Treatment (n=255)	Standard Treatment (n=203)	P-value	SMD
**Age, mean (SD), years**	67.65 (7.74)	66.43 (7.86)	0.096	0.157
**Sex, No. (%)**			0.303	0.106
**Female**	101 (39.6)	70 (34.5)		
**Male**	154 (60.4)	133 (65.5)		
**Race/Ethnicity, No. (%)**			0.166	0.211
**Black**	72 (28.2)	66 (32.5)		
**Hispanic**[a]	9 (3.5)	13 (6.4)		
**Other**[b]	2 (0.8)	4 (2.0)		
**White**	172 (67.5)	120 (59.1)		
**History of CVD, No. (%)**	32 (12.5)	21 (10.3)	0.558	0.069
**Systolic BP, mean (SD)**	136.04 (16.88)	138.42 (16.06)	0.127	0.144
**Diastolic BP, mean (SD)**	76.59 (10.72)	79.38 (12.17)	0.01	0.243
**Orthostatic Hypotension, No. (%)**[c]	20 (7.8)	15 (7.4)	0.996	0.017
**eGFR, mean (SD), mL/min/1.73m^2**[d]	71.79 (19.19)	73.39 (20.76)	0.395	0.08
**eGFR < 60 mL/min/1.73m2, No. (%)**	67 (26.3)	56 (27.6)	0.835	0.03
**Body Mass Index, mean (SD), kg/m^2**	29.52 (5.13)	29.56 (5.13)	0.939	0.007
**HDL cholesterol, mean (SD), mg/dL**	53.31 (14.39)	54.19 (16.10)	0.539	0.057
**Education, No. (%)**			0.193	0.205
**College degree**	114 (44.7)	81 (39.9)		
**HS diploma**	41 (16.1)	25 (12.3)		
**Less than HS**	15 (5.9)	20 (9.9)		
**Post HS**	85 (33.3)	77 (37.9)		
**Smoking Status, No. (%)**			0.293	0.148
**Current**	37 (14.5)	22 (10.8)		
**Former**	109 (42.7)	81 (39.9)		
**Never**	109 (42.7)	100 (49.3)		

[a] Hispanic race/ethnicity encompasses a self-report of being Spanish, Hispanic or Latino origin, independent of any other race/ethnicity designation.

[b] Other race/ethnicity includes categories of Asian, American Indian/Native Alaskan, Native Hawaiian/Pacific Islander, or other.

[c] Defined as a standing systolic BP minus seated systolic BP ≤ -20 mm Hg or a standing diastolic BP minus seated diastolic BP ≤ -10 mm Hg.

[d] Based on the 4-variable Modification of Diet in Renal Disease equation.

© 2023 Rashid T et al. *JAMA Network Open.*

eResults 2. Superficially Located WM (SWM)

White Matter Lesions

For the intensive treatment group, possible smaller increase ($p<0.05$, uncorrected) of WML volume (compared to the standard treatment group) over time was noted in superficially located WM (SWM) regions such as the left middle occipital WM, the left precentral WM, the right supramarginal WM, the right angular WM and the right superior occipital WM (eFigure A). The effect size, mean change per ROI and mean at baseline and follow-up per treatment group for all the SWM ROIs is reported in eTable 5.

DTI – Fractional Anisotropy and Mean Diffusivity

Possible slower decreases ($p<0.05$, uncorrected) in the mean FA for the intensive treatment group, compared to the standard treatment group were observed in the middle temporal WM regions (eFigure B). The effect size, mean change per ROI and mean at baseline and follow-up per treatment group for all the SWM ROIs are reported in eTable 7.

Interestingly, the right postcentral WM region showed a possible larger increase in mean MD for the intensive treatment group compared to the standard treatment group (eFigure C). The effect size, mean change per ROI and mean at baseline and follow-up per treatment group for all the SWM ROIs are reported in eTable 9.

© 2023 Rashid T et al. *JAMA Network Open.*

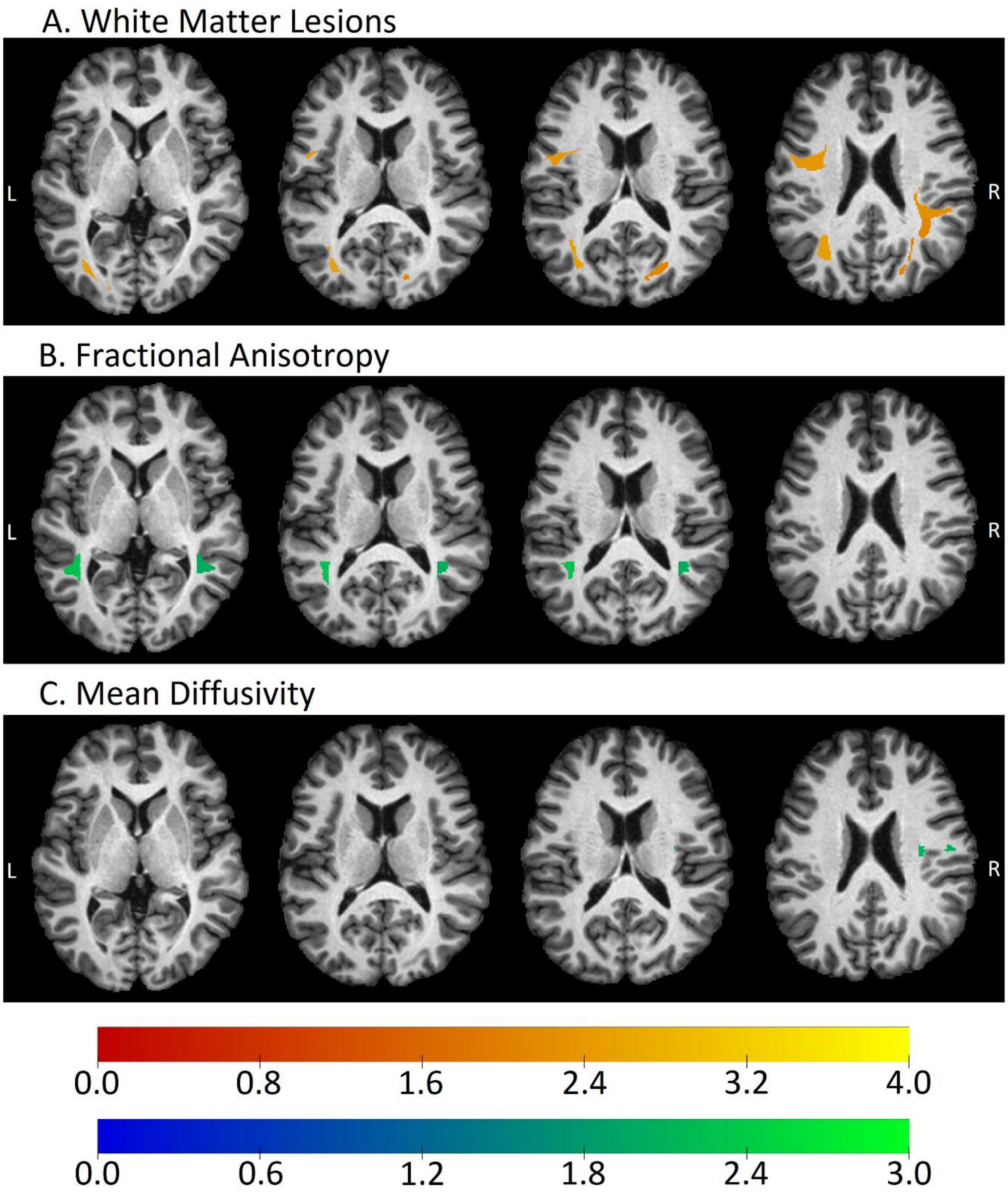

eFigure. Patterns of Changes ($p<0.05$, uncorrected) in the Type III White Matter Parcellation Map (WMPM) Derived Deep White Matter Regions-of-Interest (ROIs) When Comparing Intensive Treatment Group With Standard Treatment Group. (A) changes in white matter lesions (WML), (B) changes in diffusion tensor imaging (DTI) fractional anisotropy (FA) and (C) changes in DTI mean diffusivity (MD). The red-yellow color scale indicates effect size, that is, smaller increase over time for the intensive treatment group compared to the standard treatment group. The blue-green color scale indicates effect size, that is, larger increase for the intensive treatment group compared to the standard treatment group.

© 2023 Rashid T et al. *JAMA Network Open.*

eTable 4. Changes in White Matter Lesions (WML) Derived in Deep White Matter (DWM) Regions-of-Interest (ROIs) Between Intensive Treatment Group and Standard Treatment Group

Label – ROI Name	Effect Size	P-Value	P-Value (FDR)	Mean Change (Intensive Treatment Group) (95% CI)	Mean Change (Standard Treatment Group) (95% CI)	Mean at Baseline (Intensive Treatment Group) (95% CI)	Mean at Follow-up (Intensive Treatment Group) (95% CI)	Mean at Baseline (Standard Treatment Group) (95% CI)	Mean at Follow-up (Standard Treatment Group) (95% CI)
36 - Anterior corona radiata left	3.98	0	0.003	30.25 (15.99 to 44.52)	80.51 (53.82 to 107.21)	134.23 (109.06 to 159.41)	161.51 (126.76 to 196.26)	119.68 (95.27 to 144.09)	189.11 (141.88 to 236.34)
56 - Tapatum left	2.91	0.004	0.046	11.8 (4.39 to 19.22)	27.18 (19.38 to 34.99)	72.07 (55.49 to 88.65)	77.54 (59.96 to 95.12)	72.31 (53.57 to 91.05)	93.2 (70.76 to 115.64)
43 - Superior fronto-occipital fasciculus left	2.9	0.004	0.046	3.24 (0.65 to 5.82)	9.44 (5.46 to 13.43)	11.15 (8.28 to 14.02)	14.21 (10.25 to 18.16)	10.94 (7.88 to 14)	19.59 (13.59 to 25.6)
38 - Posterior corona radiata left	2.82	0.005	0.046	26 (12.94 to 39.06)	52.27 (34.75 to 69.8)	115.86 (83.59 to 148.12)	131.16 (94.18 to 168.13)	93.34 (64.77 to 121.9)	138.95 (96.81 to 181.09)
35 - Posterior thalamic radiation left	2.76	0.006	0.046	53.02 (29.81 to 76.24)	106.86 (73.41 to 140.31)	305.91 (242.87 to 368.95)	356.55 (277.74 to 435.36)	305.95 (240.42 to 371.49)	383.75 (302.98 to 464.51)
53 - Splenium of corpus callosum left	2.72	0.007	0.046	45.39 (25.07 to 65.7)	82.95 (58.72 to 107.18)	183.88 (138.14 to 229.62)	217.84 (162.64 to 273.05)	156.51 (113.69 to 199.34)	218.41 (162.92 to 273.91)
95 - Posterior thalamic radiation right	2.66	0.008	0.047	49.52 (24.3 to 74.74)	102.62 (71.02 to 134.21)	267.03 (215.59 to 318.48)	307.65 (241.89 to 373.42)	256.32 (207.51 to 305.12)	349.53 (278.59 to 420.46)
112 - Body of corpus callosum right	2.57	0.01	0.052	19.41 (7.68 to 31.14)	41.33 (23.2 to 59.45)	80.49 (60.18 to 100.8)	95.6 (69.27 to 121.94)	63.85 (46.11 to 81.6)	107.72 (72.6 to 142.84)
113 - Splenium of corpus callosum right	2.53	0.01	0.052	44.19 (23.8 to 64.59)	81.34 (58.18 to 104.49)	174.39 (128.92 to 219.87)	200.35 (146.41 to 254.3)	135.31 (97.3 to 173.33)	203.98 (149.18 to 258.78)
96 - Anterior corona radiata right	2.5	0.01	0.052	29.63 (17.4 to 41.87)	57.79 (24.8 to 90.77)	58.59 (39.16 to 78.01)	88.18 (60.94 to 115.41)	51 (34.68 to 67.31)	106.15 (63.68 to 148.62)
98 - Posterior corona radiata right	2.29	0.02	0.082	39.75 (22.89 to 56.62)	69.14 (46.2 to 92.07)	136.98 (97.18 to 176.77)	159.62 (110.88 to 208.36)	111.44 (77.21 to 145.67)	176.26 (125.79 to 226.74)
33 - Anterior limb of internal capsule left	2.2	0.03	0.095	5.33 (2.7 to 7.97)	13.17 (5.43 to 20.92)	17.53 (9.28 to 25.79)	19.29 (10.83 to 27.75)	18.03 (9.46 to 26.6)	25.02 (14.12 to 35.92)
115 - Tapatum right	2.14	0.03	0.101	9.15 (4.09 to 14.21)	17.85 (11.55 to 24.14)	45.65 (35.18 to 56.11)	49.06 (37 to 61.11)	45.47 (34.63 to 56.31)	63.76 (48.48 to 79.04)
39 - Cingulum (cingulate gyrus) left	1.9	0.06	0.161	0.19 (-0.13 to 0.51)	1.21 (-0.15 to 2.56)	0.72 (0.36 to 1.08)	1.17 (0.29 to 2.04)	1.31 (0.54 to 2.09)	2.52 (0.64 to 4.41)
37 - Superior corona radiata left	1.88	0.06	0.161	61.77 (32.42 to 91.13)	99.15 (53.15 to 145.16)	162.93 (110.3 to 215.57)	235.4 (160.69 to 310.11)	123.29 (77.08 to 169.49)	239.05 (148.12 to 329.99)
99 - Cingulum (cingulate gyrus) right	-1.83	0.07	0.164	0.49 (0.1 to 0.87)	-0.09 (-0.5 to 0.33)	1.66 (0.23 to 3.09)	1.87 (0.42 to 3.33)	0.68 (0.26 to 1.09)	0.62 (0.23 to 1.01)
111 - Genu of corpus callosum right	1.82	0.07	0.164	13.08 (5.47 to 20.69)	21.41 (11.54 to 31.27)	48.04 (39.65 to 56.42)	59.28 (45.83 to 72.73)	46.3 (36.77 to 55.82)	68.63 (51.54 to 85.72)
52 - Body of corpus callosum left	1.65	0.10	0.213	23.1 (10.79 to 35.42)	35.18 (18.56 to 51.8)	62.68 (43.01 to 82.35)	77.19 (51.79 to 102.58)	49.36 (32.43 to 66.3)	84.94 (53.38 to 116.49)
46 - External capsule left	1.64	0.10	0.213	2.42 (-0.18 to 5.01)	5.4 (2.26 to 8.54)	9.89 (5.49 to 14.28)	14.55 (7.22 to 21.89)	10.82 (6.04 to 15.6)	13.07 (7.96 to 18.18)

© 2023 Rashid T et al. *JAMA Network Open.*

97 - Superior corona radiata right	1.44	0.15	0.303	66.06 (31.9 to 100.22)	97.49 (48.65 to 146.33)	188.54 (126.4 to 250.67)	256.32 (164.14 to 348.5)	124.56 (75.78 to 173.33)	223.7 (139.34 to 308.07)
42 - Superior longitudinal fasciculus left	1.4	0.16	0.308	13.74 (7.9 to 19.58)	19.75 (8.88 to 30.62)	35.84 (22.83 to 48.86)	48.88 (33.04 to 64.71)	34.26 (19.03 to 49.49)	50.27 (30.76 to 69.77)
94 - Posterior limb of internal capsule right	1.34	0.18	0.328	0.85 (-0.62 to 2.32)	2.05 (0.9 to 3.19)	5.14 (2.02 to 8.27)	6.29 (2.51 to 10.06)	2.22 (0.4 to 4.04)	2.49 (1.37 to 3.6)
100 - Cingulum (hippocampus) right	1.3	0.20	0.34	0 (0 to 0)	0.02 (-0.02 to 0.05)	0.1 (-0.02 to 0.22)	0 (0 to 0)	0 (0 to 0)	0.01 (-0.01 to 0.04)
41 - Fornix(cres) Stria terminalisleft	1.14	0.25	0.423	-0.11 (-0.29 to 0.07)	0.03 (-0.13 to 0.19)	0.23 (0.08 to 0.38)	0.25 (0.08 to 0.42)	0.1 (-0.08 to 0.28)	0.17 (-0.01 to 0.35)
106 - External capsule right	1.04	0.30	0.473	2.47 (-0.87 to 5.8)	3.9 (1.41 to 6.39)	11.86 (6.36 to 17.36)	14.12 (8.35 to 19.88)	9.45 (5.03 to 13.87)	9.2 (5.92 to 12.49)
45 - Sagittal stratum left	-1.02	0.31	0.473	3.07 (-2.18 to 8.32)	0.51 (-1.3 to 2.33)	7.91 (2.33 to 13.49)	12.9 (3.96 to 21.84)	5.87 (2.91 to 8.84)	7.76 (2.38 to 13.15)
102 - Superior longitudinal fasciculus right	0.84	0.40	0.569	13.65 (5.63 to 21.66)	17.04 (9.16 to 24.92)	33.06 (20.08 to 46.04)	45.03 (28.81 to 61.25)	27.55 (16.28 to 38.82)	51.63 (34.68 to 68.58)
40 - Cingulum (hippocampus) left	0.82	0.41	0.569	-0.03 (-0.11 to 0.05)	0.01 (-0.03 to 0.05)	0.21 (0 to 0.42)	0.08 (-0.02 to 0.18)	0.02 (-0.02 to 0.06)	0.03 (0 to 0.07)
93 - Anterior limb of internal capsule right	0.79	0.43	0.569	4.42 (1.85 to 6.99)	5.8 (1.06 to 10.54)	10.46 (4.61 to 16.32)	12.7 (6.99 to 18.42)	6.55 (2.24 to 10.85)	13.14 (5.18 to 21.09)
114 - Retrolenticular part of internal capsule right	-0.78	0.44	0.569	2.39 (0.89 to 3.88)	1.31 (-0.25 to 2.88)	6.32 (1.43 to 11.2)	8.14 (1.23 to 15.05)	3.74 (1.77 to 5.7)	4.6 (2.08 to 7.12)
44 - Inferior fronto-occipital fasciculus left	-0.77	0.44	0.569	1.07 (0.37 to 1.76)	0.56 (-0.24 to 1.35)	2.75 (1.43 to 4.06)	3.2 (1.81 to 4.58)	2.07 (1.04 to 3.09)	2.25 (1.13 to 3.38)
50 - Fornix (column and body) left	0.71	0.48	0.591	-0.05 (-0.16 to 0.07)	-0.01 (-0.02 to 0.01)	0.04 (-0.04 to 0.12)	0.02 (-0.01 to 0.04)	0.01 (-0.01 to 0.02)	0 (0 to 0.01)
101 - Fornix(cres) Stria terminalis right	-0.7	0.49	0.591	0.32 (-0.09 to 0.74)	0.12 (-0.12 to 0.36)	0.14 (0.04 to 0.23)	0.46 (0.04 to 0.89)	0.12 (0.05 to 0.19)	0.29 (0.05 to 0.54)
103 - Superior fronto-occipital fasciculus right	0.61	0.54	0.639	1.87 (0.6 to 3.15)	2.04 (0.97 to 3.11)	2.71 (1.35 to 4.07)	5.1 (2.75 to 7.44)	1.99 (0.96 to 3.02)	5.21 (2.36 to 8.06)
105 - Sagittal stratum right	-0.57	0.57	0.65	2.44 (-1.32 to 6.21)	1.12 (-0.26 to 2.5)	7.56 (1.01 to 14.11)	10.25 (0.16 to 20.35)	5.27 (2.57 to 7.96)	6.73 (2.98 to 10.47)
51 - Genu of corpus callosum left	0.43	0.67	0.741	7.62 (3.82 to 11.41)	7.67 (2.45 to 12.9)	12.94 (9.14 to 16.74)	16.39 (11.46 to 21.33)	12.16 (7.81 to 16.52)	21.34 (12.89 to 29.79)
54 - Retrolenticular part of internal capsule left	-0.35	0.73	0.787	1.02 (-0.85 to 2.89)	0.44 (-0.09 to 0.98)	3.36 (0.79 to 5.94)	5.51 (0.68 to 10.34)	2.33 (0.57 to 4.1)	2.63 (0.54 to 4.73)
34 - Posterior limb of internal capsule left	-0.25	0.80	0.844	3.56 (1.22 to 5.89)	2.63 (0.33 to 4.92)	4.05 (2.11 to 5.99)	6.92 (3.67 to 10.18)	3.61 (1.85 to 5.38)	6.1 (2.62 to 9.59)
110 - Fornix (column and body) right	0.11	0.91	0.935	-0.11 (-0.38 to 0.16)	-0.09 (-0.33 to 0.14)	0.22 (0.02 to 0.43)	0.18 (0.04 to 0.31)	0.25 (0.06 to 0.44)	0.12 (-0.04 to 0.29)
104 - Inferior fronto-occipital fasciculus right	-0.07	0.94	0.942	0.35 (-0.65 to 1.35)	0.02 (-0.95 to 1)	2.91 (1.27 to 4.55)	3.24 (1.42 to 5.05)	2.98 (1.65 to 4.3)	3.18 (1.66 to 4.71)

Abbreviations: FDR, false discovery rate; CI, confidence interval; WM, white matter; GM, gray matter; DWM, deep white matter; SWM, superficially-located white matter; DTI, diffusion tensor imaging; FA, fractional anisotropy; MD, mean diffusivity, ROI, region-of-interest

© 2023 Rashid T et al. *JAMA Network Open.*

eTable 5. Changes in White Matter Lesions (WML) Derived in Superficially-Located White Matter (SWM) Regions-of-Interest (ROIs) Between Intensive Treatment Group and Standard Treatment Group

Label – ROI Name	Effect Size	P-Value	P-Value (FDR)	Mean Change (Intensive Treatment Group) (95% CI)	Mean Change (Standard Treatment Group) (95% CI)	Mean at Baseline (Intensive Treatment Group) (95% CI)	Mean at Follow-up (Intensive Treatment Group) (95% CI)	Mean at Baseline (Standard Treatment Group) (95% CI)	Mean at Follow-up (Standard Treatment Group) (95% CI)
16 - MIDDLE OCCIPITAL WM left	2.49	0.01	0.292	9.03 (3.85 to 14.21)	17.96 (10.58 to 25.34)	36.58 (25.37 to 47.78)	45.84 (31.91 to 59.77)	31.63 (22.97 to 40.29)	42.29 (29.31 to 55.28)
6 - PRECENTRAL WM left	2.37	0.02	0.292	5.07 (1.34 to 8.8)	11.66 (5.6 to 17.72)	24.13 (14.92 to 33.35)	28.77 (20.47 to 37.08)	26.44 (19.3 to 33.58)	42.13 (28.77 to 55.5)
84 - SUPRAMARGINAL WM right	2.28	0.02	0.292	3.9 (1.34 to 6.46)	10.08 (4.59 to 15.56)	12.46 (7.48 to 17.45)	14.45 (9.17 to 19.73)	10.74 (6.64 to 14.83)	21.17 (12.39 to 29.95)
70 - ANGULAR WM right	2.14	0.03	0.293	3.23 (1.31 to 5.16)	6.95 (3.49 to 10.42)	12.18 (7.76 to 16.6)	14.82 (9.14 to 20.5)	10.44 (5.87 to 15.01)	19.8 (10.86 to 28.74)
75 - SUPERIOR OCCIPITAL WM right	2.08	0.04	0.293	1.01 (-0.91 to 2.93)	4.16 (1.89 to 6.43)	9.49 (6.05 to 12.94)	10.38 (6.41 to 14.35)	7.78 (5 to 10.56)	11.64 (7.61 to 15.67)
18 - SUPERIOR TEMPORAL WM left	-1.96	0.05	0.313	1.21 (0.18 to 2.25)	-0.14 (-0.62 to 0.34)	4.25 (1.68 to 6.82)	5.45 (1.9 to 9)	3.06 (1.21 to 4.91)	2.9 (0.93 to 4.87)
66 - MIDDLE FRONTAL WM right	1.9	0.06	0.313	2.13 (0.63 to 3.63)	4.1 (1.48 to 6.72)	8.12 (3.91 to 12.33)	10.71 (6.48 to 14.95)	6.64 (3.82 to 9.46)	10.77 (5.89 to 15.66)
4 - MIDDLE FRONTAL WM left	1.84	0.07	0.313	1.43 (0.53 to 2.33)	2.8 (1.18 to 4.43)	7.14 (1.65 to 12.63)	6.36 (3.21 to 9.52)	5.82 (2.83 to 8.82)	7.37 (4.04 to 10.71)
5 - INFERIOR FRONTAL WM left	1.74	0.08	0.313	10.51 (4.45 to 16.57)	18.55 (8.81 to 28.29)	31.64 (21.7 to 41.57)	46.28 (29.22 to 63.35)	31.04 (21.75 to 40.34)	50.37 (33.27 to 67.47)
77 - MIDDLE OCCIPITAL WM right	1.7	0.09	0.313	4.74 (2.04 to 7.43)	8.09 (4.7 to 11.48)	16.73 (11.32 to 22.14)	20.84 (13.8 to 27.88)	12.93 (8.91 to 16.95)	20.82 (14.19 to 27.46)
23 - SUPRAMARGINAL WM left	1.66	0.10	0.313	1.11 (-0.25 to 2.47)	2.55 (0.91 to 4.19)	4.01 (2.47 to 5.56)	5.92 (3.13 to 8.72)	4.49 (2.32 to 6.65)	6.34 (3.54 to 9.14)
3 - SUPERIOR FRONTAL WM left	1.65	0.10	0.313	2.5 (-0.52 to 5.52)	5.83 (1.95 to 9.71)	18.19 (9.76 to 26.62)	24.27 (15.3 to 33.25)	13.39 (9.33 to 17.45)	18.84 (12.68 to 24.99)
67 - INFERIOR FRONTAL WM right	1.6	0.11	0.325	12.04 (3.91 to 20.17)	19.16 (10.41 to 27.91)	31.02 (20.01 to 42.03)	47.13 (26.52 to 67.73)	28.29 (20.1 to 36.49)	41.75 (28.47 to 55.03)
8 - ANGULAR WM left	1.56	0.12	0.328	5.81 (1.48 to 10.14)	10.7 (5.25 to 16.15)	18.86 (11.44 to 26.28)	26.38 (13.36 to 39.4)	18.97 (12.03 to 25.92)	31.79 (19.54 to 44.05)
73 - LINGUAL WM right	1.36	0.18	0.424	0.23 (-0.12 to 0.57)	0.5 (0.2 to 0.8)	0.74 (0.36 to 1.11)	1.08 (0.57 to 1.58)	0.65 (0.28 to 1.01)	0.97 (0.47 to 1.46)
80 - INFERIOR TEMPORAL WM right	-1.35	0.18	0.424	0.16 (-0.03 to 0.34)	-0.07 (-0.28 to 0.15)	0.31 (-0.14 to 0.77)	0.41 (-0.23 to 1.04)	0.22 (0.02 to 0.42)	0.24 (-0.03 to 0.52)
14 - SUPERIOR OCCIPITAL WM left	1.27	0.21	0.458	2.82 (0.78 to 4.85)	4.24 (2.43 to 6.04)	12.39 (8.99 to 15.79)	15 (10.31 to 19.69)	9.15 (6.45 to 11.86)	12.48 (8.88 to 16.09)
9 - PRE-CUNEUS WM left	1.16	0.25	0.51	1.04 (0.29 to 1.79)	1.5 (0.65 to 2.36)	4.33 (2.91 to 5.76)	4.94 (3.49 to 6.4)	3.31 (2.03 to 4.6)	4.06 (2.37 to 5.75)

© 2023 Rashid T et al. *JAMA Network Open.*

79 - SUPERIOR TEMPORAL WM right	1.14	0.26	0.51	1.71 (0.02 to 3.39)	3 (0.75 to 5.25)	8.98 (5.25 to 12.7)	10.3 (5.72 to 14.88)	6.23 (3.32 to 9.14)	9.46 (4.49 to 14.42)
76 - INFERIOR OCCIPITAL WM right	1.08	0.28	0.537	0.02 (-0.07 to 0.11)	0.15 (-0.09 to 0.39)	0.07 (0 to 0.13)	0.08 (-0.01 to 0.16)	0.04 (-0.02 to 0.1)	0.13 (-0.09 to 0.36)
20 - MIDDLE TEMPORAL WM left	0.86	0.39	0.703	4.39 (1.55 to 7.23)	6.22 (2.06 to 10.39)	15.58 (9.62 to 21.53)	18.98 (11.52 to 26.43)	15.59 (9.77 to 21.41)	24.3 (15.08 to 33.52)
83 - MIDDLE FRONTO-ORBITAL WM right	-0.77	0.439	0.747	0.01 (-0.01 to 0.03)	0 (0 to 0)	0.02 (-0.02 to 0.06)	0.01 (-0.01 to 0.02)	0.07 (-0.07 to 0.22)	0 (0 to 0)
69 - POSTCENTRAL WM right	-0.75	0.452	0.747	4.95 (-0.36 to 10.26)	2.1 (0.89 to 3.31)	4.49 (2.22 to 6.77)	8.67 (2.6 to 14.73)	3.51 (0.74 to 6.29)	5.51 (2.12 to 8.9)
64 - SUPERIOR PARIETAL WM right	0.55	0.58	0.865	13.64 (2.09 to 25.19)	15.28 (7.7 to 22.86)	41.77 (25.78 to 57.77)	50.9 (29.36 to 72.45)	24.22 (12.8 to 35.65)	47.49 (20.67 to 74.32)
68 - PRECENTRAL WM right	0.51	0.613	0.865	17.81 (7.53 to 28.08)	19.68 (10.31 to 29.05)	45.31 (28.66 to 61.96)	64.68 (40.58 to 88.79)	33.64 (23.3 to 43.97)	53.83 (37.17 to 70.48)
11 - LINGUAL WM left	0.48	0.634	0.865	0.25 (-0.09 to 0.59)	0.37 (0.01 to 0.73)	2.35 (2.01 to 2.69)	2.53 (2.08 to 2.98)	2.38 (2.01 to 2.75)	2.72 (2.2 to 3.25)
85 - RECTUS WM right	-0.47	0.635	0.865	0.02 (-0.04 to 0.08)	0 (0 to 0)	0.05 (0 to 0.1)	0.06 (-0.04 to 0.16)	0 (0 to 0)	0 (0 to 0)
7 - POSTCENTRAL WM left	0.47	0.638	0.865	1.64 (-0.01 to 3.28)	1.56 (0.34 to 2.79)	5.97 (2.75 to 9.19)	7.03 (2.72 to 11.34)	3.26 (0.27 to 6.25)	4.07 (1.33 to 6.81)
81 - MIDDLE TEMPORAL WM right	0.43	0.666	0.873	5.24 (1.97 to 8.51)	5.73 (1.7 to 9.76)	13.96 (8.93 to 19)	19.43 (11.97 to 26.88)	12.88 (8.04 to 17.73)	19.45 (12.05 to 26.84)
74 - FUSIFORM WM right	0.4	0.691	0.875	0.01 (-0.01 to 0.03)	0.02 (-0.02 to 0.07)	0 (0 to 0)	0.01 (-0.01 to 0.03)	0 (0 to 0)	0.02 (-0.02 to 0.06)
10 - CUNEUS WM left	0.35	0.724	0.888	0.99 (-0.58 to 2.56)	1.45 (-0.14 to 3.04)	5.25 (3.52 to 6.99)	5.45 (3.32 to 7.59)	5.07 (3.62 to 6.51)	6.3 (4.18 to 8.41)
15 - INFERIOR OCCIPITAL WM left	-0.32	0.75	0.89	0 (0 to 0.01)	-0.01 (-0.06 to 0.05)	0.28 (-0.17 to 0.74)	0 (0 to 0.01)	0.01 (-0.01 to 0.04)	0.01 (-0.01 to 0.04)
1 - SUPERIOR PARIETAL WM left	-0.23	0.815	0.938	11.21 (2.34 to 20.09)	8.74 (4.7 to 12.78)	31.33 (15.17 to 47.49)	41.11 (19.84 to 62.38)	21.21 (9.93 to 32.49)	31.29 (12.57 to 50.02)
21 - LATERAL FRONTO-ORBITAL WM left	-0.19	0.852	0.953	0.25 (-0.14 to 0.64)	0.17 (-0.2 to 0.53)	0.75 (0.31 to 1.18)	0.99 (0.45 to 1.53)	1.06 (0.34 to 1.78)	1.2 (0.27 to 2.14)
65 - SUPERIOR FRONTAL WM right	0.08	0.938	0.993	9.63 (2.01 to 17.25)	9.16 (4.83 to 13.49)	28.87 (15.48 to 42.26)	37.6 (24.19 to 51)	15.9 (10.64 to 21.17)	25.35 (17.54 to 33.16)
19 - INFERIOR TEMPORAL WM left	0.06	0.956	0.993	0.98 (-0.8 to 2.76)	0.54 (-0.28 to 1.35)	1.14 (-0.66 to 2.94)	2.28 (-1.37 to 5.93)	0.43 (0.1 to 0.76)	0.92 (-0.05 to 1.89)
72 - CUNEUS WM right	-0.04	0.967	0.993	0.02 (-0.02 to 0.07)	0.03 (-0.01 to 0.07)	0.04 (0.02 to 0.07)	0.08 (0.04 to 0.12)	0.04 (0.01 to 0.07)	0.05 (0.02 to 0.09)
82 - LATERAL FRONTO-ORBITAL WM right	0.01	0.994	0.994	0.44 (0.09 to 0.8)	0.32 (-0.31 to 0.95)	0.44 (0.22 to 0.67)	0.97 (0.46 to 1.48)	1.58 (0.73 to 2.43)	1.54 (0.65 to 2.42)

Abbreviations: FDR, false discovery rate; CI, confidence interval; WM, white matter; GM, gray matter; DWM, deep white matter; SWM, superficially-located white matter; DTI, diffusion tensor imaging; FA, fractional anisotropy; MD, mean diffusivity, ROI, region-of-interest

© 2023 Rashid T et al. *JAMA Network Open.*

eTable 6. Changes in Diffusion Tensor Imaging (DTI) Fractional Anisotropy (FA) Derived in Deep White Matter (DWM) Regions-of-Interest (ROIs) Between Intensive Treatment Group and Standard Treatment Group

Label – ROI Name	Effect Size	P-Value	P-Value (FDR)	Mean Change (Intensive Treatment Group) (95% CI)	Mean Change (Standard Treatment Group) (95% CI)	Mean at Baseline (Intensive Treatment Group) (95% CI)	Mean at Follow-up (Intensive Treatment Group) (95% CI)	Mean at Baseline (Standard Treatment Group) (95% CI)	Mean at Follow-up (Standard Treatment Group) (95% CI)
35 - Posterior thalamic radiation left	-2.59	0.01	0.331	-0.0003 (-0.0052 to 0.0047)	-0.005 (-0.0105 to 0.0005)	0.4056 (0.4002 to 0.4109)	0.4094 (0.4024 to 0.4165)	0.4076 (0.4029 to 0.4123)	0.404 (0.3969 to 0.4112)
43 - Superior fronto-occipital fasciculus left	-2.28	0.023	0.331	0.0108 (0.0043 to 0.0173)	0.0002 (-0.0055 to 0.006)	0.3243 (0.3188 to 0.3297)	0.3373 (0.3294 to 0.3451)	0.3274 (0.3216 to 0.3331)	0.3271 (0.32 to 0.3342)
33 - Anterior limb of internal capsule left	-2.16	0.031	0.331	0.0061 (0.0013 to 0.0109)	-0.0005 (-0.0053 to 0.0043)	0.4526 (0.4469 to 0.4582)	0.4632 (0.4569 to 0.4695)	0.4558 (0.451 to 0.4607)	0.4592 (0.4529 to 0.4654)
56 - Tapatum left	-2.08	0.038	0.331	0.0025 (-0.0045 to 0.0096)	-0.0041 (-0.0126 to 0.0045)	0.4023 (0.3956 to 0.409)	0.4087 (0.4002 to 0.4172)	0.4024 (0.396 to 0.4088)	0.398 (0.3886 to 0.4074)
45 - Sagittal stratum left	-1.99	0.047	0.331	-0.0046 (-0.0089 to -0.0004)	-0.0098 (-0.0146 to -0.005)	0.4083 (0.4031 to 0.4135)	0.408 (0.4017 to 0.4142)	0.4157 (0.4116 to 0.4199)	0.4081 (0.4021 to 0.4141)
96 - Anterior corona radiata right	-1.99	0.047	0.331	-0.0013 (-0.0044 to 0.0017)	-0.0048 (-0.0079 to -0.0017)	0.3178 (0.3133 to 0.3223)	0.3201 (0.3148 to 0.3254)	0.3217 (0.3175 to 0.3258)	0.3187 (0.3134 to 0.3241)
36 - Anterior corona radiata left	-1.79	0.074	0.445	-0.0018 (-0.0046 to 0.0011)	-0.0053 (-0.0084 to -0.0023)	0.3147 (0.3104 to 0.3189)	0.3175 (0.3123 to 0.3227)	0.3182 (0.3142 to 0.3223)	0.3148 (0.3094 to 0.3203)
105 - Sagittal stratum right	-1.62	0.107	0.561	-0.0034 (-0.0083 to 0.0014)	-0.0096 (-0.0151 to -0.0041)	0.4073 (0.4021 to 0.4126)	0.4074 (0.4011 to 0.4136)	0.4104 (0.4064 to 0.4144)	0.4038 (0.3975 to 0.41)
95 - Posterior thalamic radiation right	-1.46	0.145	0.625	-0.0042 (-0.0094 to 0.0009)	-0.0072 (-0.0128 to -0.0016)	0.4177 (0.4121 to 0.4233)	0.4183 (0.4112 to 0.4253)	0.4153 (0.4105 to 0.4201)	0.4116 (0.4047 to 0.4185)
38 - Posterior corona radiata left	-1.44	0.15	0.625	0.0027 (-0.0007 to 0.0062)	-0.0007 (-0.0041 to 0.0026)	0.3689 (0.3641 to 0.3737)	0.375 (0.3693 to 0.3808)	0.3741 (0.3694 to 0.3788)	0.3725 (0.3658 to 0.3792)
51 - Genu of corpus callosum left	-1.38	0.169	0.625	-0.0091 (-0.015 to -0.0032)	-0.0145 (-0.0202 to -0.0087)	0.4317 (0.4241 to 0.4393)	0.4305 (0.4209 to 0.44)	0.4428 (0.436 to 0.4496)	0.4314 (0.4218 to 0.4409)
34 - Posterior limb of internal capsule left	-1.3	0.196	0.625	0.007 (0.0027 to 0.0114)	0.0032 (-0.0014 to 0.0077)	0.5535 (0.5476 to 0.5595)	0.5662 (0.5604 to 0.572)	0.5559 (0.551 to 0.5607)	0.5594 (0.5528 to 0.5661)

© 2023 Rashid T et al. *JAMA Network Open.*

115 - Tapatum right	-1.25	0.211	0.625	-0.0024 (-0.0084 to 0.0037)	-0.0067 (-0.0134 to 0)	0.4369 (0.4292 to 0.4446)	0.4375 (0.4286 to 0.4463)	0.4292 (0.422 to 0.4364)	0.4199 (0.41 to 0.4298)
100 - Cingulum (hippocampus) right	-1.23	0.219	0.625	0.0023 (-0.0048 to 0.0095)	-0.0026 (-0.0103 to 0.0052)	0.3121 (0.3063 to 0.3178)	0.3164 (0.309 to 0.3238)	0.3099 (0.3047 to 0.315)	0.3067 (0.2986 to 0.3148)
41 - Fornix(cres) Stria terminalisleft	-1.19	0.236	0.625	-0.0127 (-0.0164 to -0.0089)	-0.0162 (-0.0215 to -0.011)	0.4281 (0.4235 to 0.4328)	0.4179 (0.4132 to 0.4227)	0.4319 (0.4274 to 0.4365)	0.4185 (0.4132 to 0.4238)
42 - Superior longitudinal fasciculus left	-1.16	0.246	0.625	0.0022 (-0.0016 to 0.0061)	-0.0009 (-0.0051 to 0.0033)	0.3878 (0.383 to 0.3926)	0.3938 (0.3886 to 0.3991)	0.3909 (0.3869 to 0.395)	0.3907 (0.3849 to 0.3965)
103 - Superior fronto-occipital fasciculus right	-1.14	0.255	0.625	0.0112 (0.0038 to 0.0187)	0.0056 (-0.0007 to 0.0119)	0.3419 (0.3356 to 0.3482)	0.3564 (0.3486 to 0.3643)	0.3447 (0.3385 to 0.3509)	0.3487 (0.3402 to 0.3572)
50 - Fornix (column and body) left	-1.07	0.286	0.625	0.0004 (-0.0003 to 0.0011)	0 (0 to 0)	0.0001 (-0.0001 to 0.0002)	0.0005 (-0.0002 to 0.0012)	0 (0 to 0)	0 (0 to 0)
54 - Retrolenticular part of internal capsule left	-1.07	0.287	0.625	-0.003 (-0.0084 to 0.0023)	-0.0085 (-0.0143 to -0.0027)	0.4424 (0.4376 to 0.4471)	0.4431 (0.4374 to 0.4488)	0.4422 (0.438 to 0.4465)	0.4364 (0.43 to 0.4428)
111 - Genu of corpus callosum right	-1.04	0.298	0.625	-0.0098 (-0.015 to -0.0047)	-0.0131 (-0.0179 to -0.0083)	0.4149 (0.4078 to 0.422)	0.4109 (0.4028 to 0.4189)	0.4202 (0.4135 to 0.4269)	0.4102 (0.4014 to 0.4191)
47 - Uncinate fasciculus left	0.9	0.369	0.717	-0.0032 (-0.0095 to 0.003)	-0.0008 (-0.0081 to 0.0065)	0.2975 (0.2905 to 0.3045)	0.2987 (0.2907 to 0.3066)	0.3061 (0.2992 to 0.313)	0.3104 (0.3017 to 0.3191)
40 - Cingulum (hippocampus) left	-0.89	0.375	0.717	-0.0035 (-0.0104 to 0.0034)	-0.0069 (-0.0142 to 0.0005)	0.3299 (0.3244 to 0.3354)	0.3301 (0.3231 to 0.3371)	0.3329 (0.3282 to 0.3375)	0.3262 (0.3187 to 0.3337)
107 - Uncinate fasciculus right	0.74	0.461	0.771	-0.0062 (-0.0139 to 0.0015)	-0.0024 (-0.013 to 0.0082)	0.4008 (0.3931 to 0.4086)	0.4004 (0.391 to 0.4098)	0.4071 (0.3993 to 0.4149)	0.4093 (0.3981 to 0.4206)
97 - Superior corona radiata right	0.69	0.488	0.771	-0.0021 (-0.0047 to 0.0005)	-0.0018 (-0.0045 to 0.001)	0.382 (0.3771 to 0.3868)	0.3812 (0.3767 to 0.3858)	0.3815 (0.3777 to 0.3853)	0.3793 (0.3742 to 0.3843)
46 - External capsule left	-0.67	0.501	0.771	-0.0017 (-0.0074 to 0.0041)	-0.0071 (-0.0129 to -0.0014)	0.3385 (0.3334 to 0.3435)	0.3405 (0.3338 to 0.3471)	0.34 (0.3353 to 0.3448)	0.3355 (0.3289 to 0.3421)
112 - Body of corpus callosum right	0.66	0.508	0.771	-0.0163 (-0.0193 to -0.0132)	-0.0147 (-0.0181 to -0.0113)	0.4372 (0.4299 to 0.4445)	0.4254 (0.4178 to 0.4331)	0.4434 (0.437 to 0.4498)	0.4276 (0.4193 to 0.436)
104 - Inferior fronto-occipital fasciculus right	-0.64	0.519	0.771	0.0007 (-0.0036 to 0.0051)	-0.0014 (-0.006 to 0.0032)	0.3672 (0.3618 to 0.3727)	0.3717 (0.3653 to 0.3782)	0.3719 (0.3676 to 0.3762)	0.3707 (0.3641 to 0.3773)

© 2023 Rashid T et al. *JAMA Network Open.*

37 - Superior corona radiata left	0.64	0.524	0.771	-0.0029 (-0.0054 to -0.0004)	-0.0027 (-0.0052 to -0.0001)	0.3907 (0.386 to 0.3954)	0.3904 (0.3854 to 0.3953)	0.3905 (0.3865 to 0.3944)	0.3857 (0.3805 to 0.391)
52 - Body of corpus callosum left	0.62	0.532	0.771	-0.0137 (-0.0171 to -0.0104)	-0.0124 (-0.0161 to -0.0088)	0.4415 (0.4346 to 0.4484)	0.4329 (0.4253 to 0.4406)	0.4479 (0.4415 to 0.4543)	0.4357 (0.4274 to 0.444)
110 - Fornix (column and body) right	0.59	0.555	0.777	-0.0219 (-0.0312 to -0.0127)	-0.0195 (-0.0294 to -0.0097)	0.3473 (0.3387 to 0.3558)	0.329 (0.3193 to 0.3386)	0.3527 (0.3439 to 0.3614)	0.3406 (0.3307 to 0.3505)
99 - Cingulum (cingulate gyrus) right	0.39	0.695	0.898	0.0013 (-0.0041 to 0.0067)	0.0007 (-0.0049 to 0.0064)	0.3365 (0.3296 to 0.3434)	0.3394 (0.3316 to 0.3472)	0.3357 (0.3293 to 0.3422)	0.3391 (0.3309 to 0.3473)
106 - External capsule right	-0.35	0.726	0.898	-0.0009 (-0.0053 to 0.0034)	-0.0018 (-0.0064 to 0.0028)	0.2896 (0.2854 to 0.2938)	0.2913 (0.2856 to 0.297)	0.291 (0.287 to 0.2949)	0.2898 (0.2837 to 0.296)
94 - Posterior limb of internal capsule right	0.32	0.749	0.898	-0.0017 (-0.0066 to 0.0032)	-0.0023 (-0.0075 to 0.003)	0.5655 (0.5597 to 0.5713)	0.5711 (0.5652 to 0.577)	0.5657 (0.561 to 0.5703)	0.5646 (0.557 to 0.5721)
44 - Inferior fronto-occipital fasciculus left	0.29	0.776	0.898	-0.0006 (-0.0055 to 0.0042)	-0.0019 (-0.0069 to 0.0031)	0.3952 (0.3895 to 0.4008)	0.3975 (0.3904 to 0.4046)	0.4023 (0.3976 to 0.407)	0.399 (0.392 to 0.4059)
53 - Splenium of corpus callosum left	0.27	0.788	0.898	-0.0072 (-0.0131 to -0.0013)	-0.007 (-0.0138 to -0.0001)	0.5011 (0.4938 to 0.5084)	0.4999 (0.4908 to 0.509)	0.5028 (0.4965 to 0.509)	0.4958 (0.4865 to 0.5051)
98 - Posterior corona radiata right	-0.23	0.815	0.898	-0.0029 (-0.0062 to 0.0003)	-0.0047 (-0.0084 to -0.001)	0.3682 (0.3622 to 0.3742)	0.3669 (0.3609 to 0.373)	0.368 (0.3631 to 0.3728)	0.3639 (0.3571 to 0.3706)
114 - Retrolenticular part of internal capsule right	0.22	0.824	0.898	-0.0055 (-0.0113 to 0.0003)	-0.0075 (-0.0144 to -0.0006)	0.4306 (0.4257 to 0.4355)	0.4279 (0.4207 to 0.4352)	0.4297 (0.425 to 0.4345)	0.4222 (0.4143 to 0.43)
93 - Anterior limb of internal capsule right	0.2	0.84	0.898	0.0026 (-0.0025 to 0.0078)	0.0025 (-0.003 to 0.008)	0.4825 (0.4766 to 0.4883)	0.4918 (0.4851 to 0.4985)	0.4836 (0.4784 to 0.4888)	0.4857 (0.4778 to 0.4935)
101 - Fornix(cres) Stria terminalis right	0.18	0.854	0.898	-0.0096 (-0.0141 to -0.0051)	-0.0101 (-0.0158 to -0.0044)	0.4017 (0.3964 to 0.4071)	0.3911 (0.3864 to 0.3958)	0.4043 (0.3994 to 0.4093)	0.398 (0.3923 to 0.4038)
102 - Superior longitudinal fasciculus right	-0.18	0.855	0.898	0.0034 (-0.0003 to 0.0071)	0.0028 (-0.0011 to 0.0067)	0.3808 (0.3763 to 0.3854)	0.3886 (0.3833 to 0.3939)	0.3842 (0.3802 to 0.3882)	0.3879 (0.3823 to 0.3936)
39 - Cingulum (cingulate gyrus) left	0.08	0.936	0.959	-0.0039 (-0.0087 to 0.001)	-0.0036 (-0.0089 to 0.0018)	0.3213 (0.3142 to 0.3283)	0.3205 (0.3122 to 0.3289)	0.3314 (0.3249 to 0.3379)	0.3304 (0.3216 to 0.3392)
113 - Splenium of corpus callosum right	-0.04	0.967	0.967	-0.005 (-0.0111 to 0.0011)	-0.0056 (-0.0126 to 0.0014)	0.5148 (0.5079 to 0.5217)	0.5156 (0.507 to 0.5243)	0.5159 (0.5097 to 0.5222)	0.511 (0.5016 to 0.5204)

© 2023 Rashid T et al. *JAMA Network Open.*

Abbreviations: FDR, false discovery rate; CI, confidence interval; WM, white matter; GM, gray matter; DWM, deep white matter; SWM, superficially-located white matter; DTI, diffusion tensor imaging; FA, fractional anisotropy; MD, mean diffusivity, ROI, region-of-interest

© 2023 Rashid T et al. *JAMA Network Open.*

eTable 7. Changes in Diffusion Tensor Imaging (DTI) Fractional Anisotropy (FA) Derived in Superficially-Located White Matter (SWM) Regions-of-Interest (ROIs) Between Intensive Treatment Group and Standard Treatment Group

Label – ROI Name	Effect Size	P-Value	P-Value (FDR)	Mean Change (Intensive Treatment Group) (95% CI)	Mean Change (Standard Treatment Group) (95% CI)	Mean at Baseline (Intensive Treatment Group) (95% CI)	Mean at Follow-up (Intensive Treatment Group) (95% CI)	Mean at Baseline (Standard Treatment Group) (95% CI)	Mean at Follow-up (Standard Treatment Group) (95% CI)
20 - MIDDLE TEMPORAL WM left	-2.28	0.023	0.679	-0.0002 (-0.0046 to 0.0041)	-0.0059 (-0.0105 to -0.0013)	0.3566 (0.3515 to 0.3617)	0.3607 (0.3546 to 0.3668)	0.359 (0.3542 to 0.3637)	0.3568 (0.3503 to 0.3632)
81 - MIDDLE TEMPORAL WM right	-2.02	0.044	0.679	-0.0007 (-0.005 to 0.0036)	-0.0058 (-0.0103 to -0.0014)	0.3281 (0.3239 to 0.3322)	0.3301 (0.3246 to 0.3357)	0.3291 (0.3251 to 0.333)	0.3257 (0.32 to 0.3314)
7 - POSTCENTRAL WM left	-1.62	0.105	0.679	0.0063 (0.001 to 0.0116)	0.0007 (-0.0048 to 0.0062)	0.358 (0.3536 to 0.3624)	0.3675 (0.3605 to 0.3745)	0.3623 (0.3585 to 0.3662)	0.3625 (0.3559 to 0.3691)
82 - LATERAL FRONTO-ORBITAL WM right	-1.58	0.115	0.679	0.0012 (-0.0044 to 0.0068)	-0.0045 (-0.0102 to 0.0012)	0.2462 (0.2409 to 0.2516)	0.2527 (0.2452 to 0.2601)	0.2503 (0.2456 to 0.2551)	0.248 (0.2402 to 0.2557)
73 - LINGUAL WM right	1.57	0.118	0.679	-0.0048 (-0.0236 to 0.014)	0.018 (-0.0042 to 0.0401)	0.0548 (0.04 to 0.0696)	0.0485 (0.0324 to 0.0646)	0.0503 (0.0348 to 0.0658)	0.0671 (0.0458 to 0.0884)
1 - SUPERIOR PARIETAL WM left	-1.46	0.144	0.679	0.0009 (-0.003 to 0.0048)	-0.0035 (-0.0078 to 0.0008)	0.3774 (0.3718 to 0.383)	0.38 (0.3734 to 0.3867)	0.3771 (0.3724 to 0.3817)	0.3736 (0.367 to 0.3802)
77 - MIDDLE OCCIPITAL WM right	-1.4	0.161	0.679	-0.002 (-0.0067 to 0.0028)	-0.0047 (-0.0096 to 0.0003)	0.3168 (0.312 to 0.3217)	0.3193 (0.3123 to 0.3263)	0.3188 (0.314 to 0.3235)	0.3161 (0.3094 to 0.3227)
6 - PRECENTRAL WM left	-1.35	0.178	0.679	0.003 (-0.0008 to 0.0068)	-0.0007 (-0.0047 to 0.0032)	0.3752 (0.3707 to 0.3797)	0.3806 (0.3748 to 0.3864)	0.3761 (0.3723 to 0.38)	0.3757 (0.3697 to 0.3817)
76 - INFERIOR OCCIPITAL WM right	-1.19	0.235	0.679	-0.0041 (-0.0091 to 0.0009)	-0.0084 (-0.0134 to -0.0034)	0.319 (0.3132 to 0.3247)	0.3188 (0.3116 to 0.3259)	0.3223 (0.3168 to 0.3279)	0.3173 (0.31 to 0.3246)
83 - MIDDLE FRONTO-ORBITAL WM right	1.12	0.262	0.679	-0.0045 (-0.0107 to 0.0017)	-0.0028 (-0.0091 to 0.0035)	0.2541 (0.2479 to 0.2603)	0.2539 (0.2458 to 0.262)	0.2526 (0.247 to 0.2582)	0.2528 (0.2444 to 0.2613)
80 - INFERIOR TEMPORAL WM right	-1.12	0.262	0.679	-0.004 (-0.0093 to 0.0013)	-0.0088 (-0.0149 to -0.0028)	0.3266 (0.3211 to 0.3321)	0.3255 (0.3188 to 0.3323)	0.3325 (0.3271 to 0.3378)	0.3255 (0.319 to 0.3321)
64 - SUPERIOR PARIETAL WM right	-1.11	0.269	0.679	-0.0039 (-0.0079 to 0.0001)	-0.0073 (-0.0115 to -0.003)	0.3894 (0.3836 to 0.3952)	0.3864 (0.3798 to 0.393)	0.3898 (0.3847 to 0.3949)	0.3824 (0.3753 to 0.3894)

© 2023 Rashid T et al. *JAMA Network Open.*

66 - MIDDLE FRONTAL WM right	-1.1	0.27	0.679	-0.0007 (-0.0044 to 0.003)	-0.0047 (-0.0092 to -0.0001)	0.3062 (0.3008 to 0.3117)	0.3072 (0.3016 to 0.3128)	0.311 (0.3064 to 0.3157)	0.3076 (0.3015 to 0.3137)
5 - INFERIOR FRONTAL WM left	-1.08	0.281	0.679	-0.0063 (-0.0091 to -0.0036)	-0.0087 (-0.0119 to -0.0055)	0.3287 (0.3245 to 0.333)	0.3271 (0.3225 to 0.3316)	0.3328 (0.3291 to 0.3365)	0.3262 (0.3213 to 0.3311)
19 - INFERIOR TEMPORAL WM left	-1.07	0.286	0.679	-0.008 (-0.0119 to -0.004)	-0.0128 (-0.0183 to -0.0074)	0.3448 (0.3394 to 0.3502)	0.3387 (0.3331 to 0.3442)	0.3547 (0.3496 to 0.3597)	0.3431 (0.3369 to 0.3493)
8 - ANGULAR WM left	-0.99	0.323	0.679	-0.0021 (-0.0058 to 0.0017)	-0.004 (-0.008 to 0)	0.3086 (0.3039 to 0.3133)	0.3095 (0.3036 to 0.3155)	0.3081 (0.3036 to 0.3126)	0.3041 (0.298 to 0.3101)
75 - SUPERIOR OCCIPITAL WM right	-0.98	0.328	0.679	-0.0001 (-0.0052 to 0.005)	-0.004 (-0.0095 to 0.0016)	0.3618 (0.355 to 0.3687)	0.3642 (0.3555 to 0.3729)	0.3626 (0.3561 to 0.3691)	0.3628 (0.3547 to 0.3709)
68 - PRECENTRAL WM right	-0.95	0.342	0.679	0.0018 (-0.0016 to 0.0052)	-0.0005 (-0.004 to 0.003)	0.363 (0.3587 to 0.3672)	0.3661 (0.3611 to 0.3712)	0.363 (0.3595 to 0.3665)	0.3636 (0.3585 to 0.3687)
79 - SUPERIOR TEMPORAL WM right	-0.93	0.351	0.679	-0.0027 (-0.0074 to 0.0021)	-0.0054 (-0.0106 to -0.0002)	0.3044 (0.3004 to 0.3084)	0.304 (0.2987 to 0.3092)	0.3049 (0.3013 to 0.3085)	0.3009 (0.2956 to 0.3062)
84 - SUPRAMARGINAL WM right	-0.91	0.362	0.679	-0.0001 (-0.0038 to 0.0035)	-0.002 (-0.0058 to 0.0019)	0.3014 (0.2964 to 0.3064)	0.3032 (0.2979 to 0.3085)	0.3049 (0.3008 to 0.3089)	0.3008 (0.2949 to 0.3067)
18 - SUPERIOR TEMPORAL WM left	-0.87	0.387	0.679	-0.001 (-0.0056 to 0.0037)	-0.0044 (-0.0095 to 0.0006)	0.3166 (0.3119 to 0.3212)	0.3186 (0.3128 to 0.3244)	0.3191 (0.3148 to 0.3233)	0.3171 (0.3111 to 0.3231)
21 - LATERAL FRONTO-ORBITAL WM left	-0.83	0.408	0.679	-0.0006 (-0.0059 to 0.0047)	-0.0055 (-0.0116 to 0.0006)	0.2695 (0.2637 to 0.2754)	0.2729 (0.2655 to 0.2803)	0.2758 (0.2704 to 0.2813)	0.2707 (0.2626 to 0.2788)
3 - SUPERIOR FRONTAL WM left	-0.8	0.421	0.679	0.002 (-0.0012 to 0.0052)	-0.0005 (-0.0041 to 0.0032)	0.342 (0.3382 to 0.3459)	0.3462 (0.3416 to 0.3508)	0.3442 (0.3408 to 0.3477)	0.344 (0.3391 to 0.3489)
10 - CUNEUS WM left	-0.8	0.423	0.679	0.0023 (-0.0038 to 0.0084)	-0.0023 (-0.0089 to 0.0043)	0.3452 (0.3371 to 0.3532)	0.3519 (0.3421 to 0.3617)	0.351 (0.3432 to 0.3589)	0.3497 (0.3395 to 0.3599)
65 - SUPERIOR FRONTAL WM right	-0.78	0.435	0.679	0.0062 (0.0024 to 0.01)	0.0031 (-0.0011 to 0.0073)	0.3378 (0.3337 to 0.3418)	0.3467 (0.342 to 0.3514)	0.3401 (0.3365 to 0.3436)	0.344 (0.3391 to 0.3489)
16 - MIDDLE OCCIPITAL WM left	-0.65	0.517	0.749	-0.0007 (-0.005 to 0.0036)	-0.002 (-0.0068 to 0.0028)	0.3055 (0.3009 to 0.3102)	0.3081 (0.3013 to 0.315)	0.3073 (0.3027 to 0.3119)	0.3059 (0.2991 to 0.3126)
15 - INFERIOR OCCIPITAL WM left	-0.65	0.519	0.749	0.0034 (-0.002 to 0.0088)	0.0001 (-0.0057 to 0.0059)	0.2922 (0.2855 to 0.299)	0.3001 (0.2914 to 0.3088)	0.2942 (0.2877 to 0.3006)	0.2967 (0.2878 to 0.3056)

© 2023 Rashid T et al. *JAMA Network Open.*

14 - SUPERIOR OCCIPITAL WM left	-0.62	0.537	0.749	0.0053 (-0.0006 to 0.0112)	0.0026 (-0.0037 to 0.0089)	0.3495 (0.3427 to 0.3563)	0.3561 (0.3469 to 0.3653)	0.3571 (0.35 to 0.3641)	0.3608 (0.351 to 0.3706)
22 - MIDDLE FRONTO-ORBITAL WM left	0.57	0.569	0.765	-0.0054 (-0.0106 to -0.0002)	-0.0046 (-0.0105 to 0.0012)	0.3044 (0.2986 to 0.3102)	0.3043 (0.2974 to 0.3113)	0.3088 (0.3028 to 0.3149)	0.3117 (0.3041 to 0.3192)
70 - ANGULAR WM right	0.39	0.693	0.846	-0.0018 (-0.0057 to 0.0021)	-0.0008 (-0.005 to 0.0033)	0.3089 (0.3026 to 0.3151)	0.31 (0.3026 to 0.3174)	0.3085 (0.3025 to 0.3146)	0.3091 (0.3011 to 0.317)
9 - PRE-CUNEUS WM left	-0.39	0.693	0.846	-0.0017 (-0.0079 to 0.0045)	-0.0028 (-0.0094 to 0.0039)	0.3035 (0.2957 to 0.3113)	0.3033 (0.294 to 0.3126)	0.3053 (0.2981 to 0.3124)	0.3004 (0.2914 to 0.3094)
24 - RECTUS WM left	0.37	0.714	0.846	0.0004 (-0.0073 to 0.0082)	0.0018 (-0.0071 to 0.0108)	0.24 (0.2336 to 0.2464)	0.244 (0.2371 to 0.2508)	0.2441 (0.2382 to 0.2501)	0.2502 (0.2424 to 0.258)
67 - INFERIOR FRONTAL WM right	-0.36	0.716	0.846	-0.0051 (-0.0088 to -0.0014)	-0.0059 (-0.0091 to -0.0027)	0.3045 (0.3003 to 0.3086)	0.3044 (0.2997 to 0.3091)	0.308 (0.3042 to 0.3117)	0.3058 (0.3012 to 0.3104)
69 - POSTCENTRAL WM right	-0.31	0.755	0.865	0.0057 (0.0002 to 0.0113)	0.0027 (-0.0028 to 0.0083)	0.3432 (0.3383 to 0.3481)	0.351 (0.3439 to 0.358)	0.3424 (0.3382 to 0.3466)	0.3446 (0.338 to 0.3512)
85 - RECTUS WM right	-0.22	0.826	0.896	0.0005 (-0.0047 to 0.0058)	-0.0029 (-0.0089 to 0.0031)	0.2768 (0.2716 to 0.2819)	0.2816 (0.2754 to 0.2879)	0.2836 (0.2786 to 0.2886)	0.2821 (0.275 to 0.2892)
74 - FUSIFORM WM right	-0.22	0.827	0.896	-0.0017 (-0.016 to 0.0126)	-0.0038 (-0.0177 to 0.0101)	0.0206 (0.0118 to 0.0293)	0.0281 (0.0146 to 0.0415)	0.0155 (0.0078 to 0.0232)	0.0174 (0.0067 to 0.028)
11 - LINGUAL WM left	0.18	0.858	0.904	-0.003 (-0.0076 to 0.0017)	-0.0028 (-0.0084 to 0.0028)	0.0033 (-0.0005 to 0.0072)	0.0017 (-0.0017 to 0.0051)	0.0033 (-0.0013 to 0.008)	0 (0 to 0)
4 - MIDDLE FRONTAL WM left	-0.1	0.922	0.946	0.0023 (-0.0019 to 0.0065)	0.0005 (-0.0041 to 0.0051)	0.3169 (0.3104 to 0.3233)	0.3219 (0.314 to 0.3298)	0.3211 (0.315 to 0.3273)	0.3222 (0.3143 to 0.33)
23 - SUPRAMARGINAL WM left	0.06	0.95	0.95	-0.0029 (-0.0072 to 0.0013)	-0.0038 (-0.0082 to 0.0006)	0.3245 (0.3198 to 0.3291)	0.3244 (0.3185 to 0.3302)	0.3248 (0.3204 to 0.3292)	0.3188 (0.3127 to 0.3249)

Abbreviations: FDR, false discovery rate; CI, confidence interval; WM, white matter; GM, gray matter; DWM, deep white matter; SWM, superficially-located white matter; DTI, diffusion tensor imaging; FA, fractional anisotropy; MD, mean diffusivity, ROI, region-of-interest

© 2023 Rashid T et al. *JAMA Network Open.*

eTable 8. Changes in Diffusion Tensor Imaging (DTI) Mean Diffusivity (MD) Derived in Deep White Matter (DWM) Regions-of-Interest (ROIs) Between Intensive Treatment Group and Standard Treatment Group

Label – ROI Name	Effect Size	P-Value	P-Value (FDR)	Mean Change (Intensive Treatment Group) (95% CI)	Mean Change (Standard Treatment Group) (95% CI)	Mean at Baseline (Intensive Treatment Group) (95% CI)	Mean at Follow-up (Intensive Treatment Group) (95% CI)	Mean at Baseline (Standard Treatment Group) (95% CI)	Mean at Follow-up (Standard Treatment Group) (95% CI)
43 - Superior fronto-occipital fasciculus left	2.25	0.025	0.571	0.0158 (0.0042 to 0.0275)	0.0317 (0.0219 to 0.0414)	0.8295 (0.7807 to 0.8783)	0.8191 (0.8049 to 0.8332)	0.796 (0.786 to 0.806)	0.8261 (0.8124 to 0.8398)
35 - Posterior thalamic radiation left	2.04	0.042	0.571	0.0154 (0.0086 to 0.0221)	0.0226 (0.0151 to 0.0301)	0.8928 (0.8788 to 0.9068)	0.9029 (0.892 to 0.9137)	0.8875 (0.8791 to 0.8959)	0.9121 (0.9 to 0.9241)
56 - Tapatum left	2	0.047	0.571	0.0335 (0.0179 to 0.0491)	0.0533 (0.0354 to 0.0711)	1.1808 (0.978 to 1.3836)	1.1123 (1.087 to 1.1377)	1.0704 (1.0519 to 1.0889)	1.1224 (1.096 to 1.1489)
115 - Tapatum right	1.88	0.06	0.571	0.0302 (0.0183 to 0.0421)	0.0473 (0.0329 to 0.0617)	1.0209 (1.0014 to 1.0404)	1.053 (1.0308 to 1.0752)	1.0259 (1.0072 to 1.0446)	1.0749 (1.0489 to 1.1009)
39 - Cingulum (cingulate gyrus) left	-1.73	0.084	0.571	0.0159 (0.0091 to 0.0227)	0.0086 (0.0013 to 0.0158)	0.8442 (0.7966 to 0.8918)	0.8362 (0.8276 to 0.8449)	0.8203 (0.813 to 0.8277)	0.8229 (0.8144 to 0.8315)
38 - Posterior corona radiata left	1.69	0.092	0.571	0.0224 (0.0174 to 0.0273)	0.0272 (0.0218 to 0.0326)	0.9206 (0.7176 to 1.1235)	0.8379 (0.8268 to 0.849)	0.8105 (0.8021 to 0.8189)	0.8365 (0.8241 to 0.8489)
96 - Anterior corona radiata right	1.67	0.095	0.571	0.0104 (0.0063 to 0.0145)	0.0132 (0.009 to 0.0174)	0.823 (0.7998 to 0.8461)	0.8201 (0.8129 to 0.8274)	0.8108 (0.8045 to 0.8171)	0.8199 (0.8121 to 0.8277)
107 - Uncinate fasciculus right	-1.32	0.186	0.858	0.0223 (0.0137 to 0.031)	0.0143 (0.0034 to 0.0252)	0.7697 (0.7616 to 0.7778)	0.7961 (0.7867 to 0.8055)	0.7754 (0.7688 to 0.7821)	0.788 (0.7765 to 0.7994)
110 - Fornix (column and body) right	-1.28	0.2	0.858	0.0368 (0.0089 to 0.0647)	0.0234 (-0.0066 to 0.0533)	1.7597 (1.7264 to 1.7929)	1.8028 (1.7692 to 1.8363)	1.7747 (1.7432 to 1.8062)	1.7731 (1.7376 to 1.8085)
99 - Cingulum (cingulate gyrus) right	-1.27	0.204	0.858	0.0104 (0.0035 to 0.0173)	0.0064 (-0.0012 to 0.0141)	0.8135 (0.7989 to 0.8281)	0.8176 (0.8096 to 0.8255)	0.8123 (0.8042 to 0.8204)	0.8101 (0.8013 to 0.8188)
101 - Fornix(cres) Stria terminalis right	-1.07	0.285	0.909	0.0298 (0.0218 to 0.0378)	0.026 (0.0174 to 0.0346)	0.8054 (0.7967 to 0.8141)	0.84 (0.8297 to 0.8503)	0.8099 (0.8026 to 0.8172)	0.8333 (0.823 to 0.8436)
51 - Genu of corpus callosum left	-1.07	0.286	0.909	0.0138 (0.0058 to 0.0219)	0.0105 (0.0039 to 0.0172)	0.9072 (0.872 to 0.9423)	0.904 (0.8943 to 0.9137)	0.8955 (0.8876 to 0.9035)	0.8998 (0.8901 to 0.9095)

© 2023 Rashid T et al. *JAMA Network Open.*

33 - Anterior limb of internal capsule left	1.03	0.305	0.909	0.0095 (0.0026 to 0.0165)	0.0167 (0.0091 to 0.0243)	0.7705 (0.761 to 0.78)	0.779 (0.7703 to 0.7877)	0.7736 (0.7655 to 0.7818)	0.7845 (0.7749 to 0.7941)
50 - Fornix (column and body) left	-0.95	0.341	0.909	0.0116 (-0.0113 to 0.0346)	0 (0 to 0)	0.0085 (-0.0082 to 0.0252)	0.0231 (-0.009 to 0.0551)	0 (0 to 0)	0 (0 to 0)
102 - Superior longitudinal fasciculus right	-0.92	0.356	0.909	0.0105 (0.0062 to 0.0147)	0.0091 (0.0043 to 0.0138)	0.7583 (0.751 to 0.7657)	0.7697 (0.7636 to 0.7759)	0.7628 (0.7576 to 0.768)	0.7677 (0.7612 to 0.7743)
100 - Cingulum (hippocampus) right	0.88	0.381	0.909	0.0106 (-0.0006 to 0.0217)	0.0184 (0.0059 to 0.0309)	0.8712 (0.8602 to 0.8822)	0.887 (0.8764 to 0.8975)	0.8706 (0.8627 to 0.8785)	0.8851 (0.8736 to 0.8967)
52 - Body of corpus callosum left	-0.83	0.409	0.909	0.0338 (0.0278 to 0.0397)	0.032 (0.025 to 0.039)	0.9167 (0.9025 to 0.9309)	0.9439 (0.9344 to 0.9535)	0.9138 (0.9059 to 0.9216)	0.9439 (0.9328 to 0.9549)
54 - Retrolenticular part of internal capsule left	0.81	0.421	0.909	0.012 (0.0055 to 0.0186)	0.0182 (0.0106 to 0.0258)	0.804 (0.7951 to 0.8128)	0.8172 (0.8092 to 0.8252)	0.8068 (0.7999 to 0.8137)	0.8213 (0.812 to 0.8307)
36 - Anterior corona radiata left	0.8	0.421	0.909	0.0129 (0.0086 to 0.0172)	0.015 (0.0107 to 0.0194)	0.8304 (0.8118 to 0.8489)	0.8334 (0.8259 to 0.8409)	0.8212 (0.815 to 0.8274)	0.834 (0.8258 to 0.8422)
40 - Cingulum (hippocampus) left	0.76	0.445	0.909	0.0056 (-0.0048 to 0.016)	0.0118 (-0.0005 to 0.0241)	0.8961 (0.8813 to 0.911)	0.9019 (0.8882 to 0.9157)	0.8935 (0.8846 to 0.9023)	0.9014 (0.8905 to 0.9123)
104 - Inferior fronto-occipital fasciculus right	-0.75	0.454	0.909	0.0186 (0.0128 to 0.0244)	0.0164 (0.0108 to 0.0221)	0.8084 (0.8001 to 0.8166)	0.8283 (0.8212 to 0.8353)	0.8117 (0.8058 to 0.8176)	0.8263 (0.8181 to 0.8345)
41 - Fornix(cres) Stria terminalisleft	0.65	0.516	0.931	0.0232 (0.0161 to 0.0302)	0.028 (0.0202 to 0.0359)	0.7757 (0.7669 to 0.7845)	0.8002 (0.7917 to 0.8086)	0.7783 (0.7709 to 0.7857)	0.8048 (0.7951 to 0.8145)
46 - External capsule left	-0.6	0.551	0.931	0.0144 (0.0077 to 0.0211)	0.0156 (0.0083 to 0.0229)	0.7851 (0.7757 to 0.7944)	0.8003 (0.7912 to 0.8094)	0.7882 (0.7804 to 0.7959)	0.7988 (0.7892 to 0.8083)
42 - Superior longitudinal fasciculus left	-0.59	0.555	0.931	0.0096 (0.0054 to 0.0138)	0.009 (0.0042 to 0.0137)	0.7739 (0.7271 to 0.8206)	0.7588 (0.7524 to 0.7651)	0.7499 (0.7443 to 0.7555)	0.7569 (0.7499 to 0.764)
103 - Superior fronto-occipital fasciculus right	0.54	0.592	0.931	0.0153 (0.0051 to 0.0256)	0.0187 (0.0098 to 0.0276)	0.7648 (0.7534 to 0.7763)	0.7768 (0.7638 to 0.7898)	0.7677 (0.7575 to 0.778)	0.7844 (0.769 to 0.7998)
106 - External capsule right	-0.53	0.593	0.931	0.0196 (0.0133 to 0.0259)	0.0179 (0.0112 to 0.0246)	0.8025 (0.7948 to 0.8103)	0.82 (0.8114 to 0.8286)	0.8043 (0.7971 to 0.8114)	0.8163 (0.8065 to 0.8261)
44 - Inferior fronto-occipital fasciculus left	-0.53	0.599	0.931	0.0126 (0.0065 to 0.0187)	0.0124 (0.0063 to 0.0185)	0.8027 (0.7945 to 0.811)	0.8179 (0.8103 to 0.8255)	0.8058 (0.7998 to 0.8118)	0.8168 (0.8087 to 0.8249)

© 2023 Rashid T et al. *JAMA Network Open.*

95 - Posterior thalamic radiation right	0.5	0.621	0.931	0.0122 (0.0053 to 0.0191)	0.0159 (0.0081 to 0.0237)	0.9527 (0.812 to 1.0934)	0.8927 (0.8827 to 0.9026)	0.8868 (0.8783 to 0.8953)	0.9002 (0.8891 to 0.9114)
98 - Posterior corona radiata right	0.46	0.648	0.938	0.0266 (0.0218 to 0.0314)	0.0281 (0.0222 to 0.034)	0.8274 (0.817 to 0.8378)	0.8543 (0.8429 to 0.8657)	0.8321 (0.8231 to 0.841)	0.8563 (0.8431 to 0.8695)
111 - Genu of corpus callosum right	-0.37	0.71	0.947	0.0152 (0.0074 to 0.0231)	0.0143 (0.007 to 0.0216)	0.9498 (0.9392 to 0.9603)	0.9653 (0.9549 to 0.9758)	0.9495 (0.9401 to 0.9589)	0.9572 (0.9461 to 0.9683)
97 - Superior corona radiata right	0.33	0.744	0.947	0.0162 (0.0123 to 0.0201)	0.0164 (0.0117 to 0.0211)	0.7439 (0.7368 to 0.7511)	0.7596 (0.7523 to 0.767)	0.7444 (0.7387 to 0.75)	0.757 (0.7489 to 0.7651)
94 - Posterior limb of internal capsule right	0.26	0.794	0.947	0.0095 (0.0041 to 0.0149)	0.0121 (0.0052 to 0.019)	0.7528 (0.6839 to 0.8217)	0.7249 (0.7181 to 0.7317)	0.7196 (0.7149 to 0.7242)	0.7264 (0.7183 to 0.7346)
45 - Sagittal stratum left	0.25	0.799	0.947	0.0104 (0.0036 to 0.0172)	0.0136 (0.0057 to 0.0215)	0.8771 (0.8671 to 0.8872)	0.89 (0.8809 to 0.8992)	0.8772 (0.8697 to 0.8846)	0.8902 (0.8808 to 0.8996)
113 - Splenium of corpus callosum right	-0.24	0.807	0.947	0.0228 (0.0125 to 0.033)	0.0247 (0.013 to 0.0365)	1.0214 (0.9033 to 1.1395)	0.9856 (0.9701 to 1.0011)	0.9572 (0.947 to 0.9673)	0.9818 (0.9664 to 0.9972)
47 - Uncinate fasciculus left	-0.24	0.81	0.947	0.0146 (0.0036 to 0.0256)	0.0177 (0.0054 to 0.03)	0.8159 (0.8061 to 0.8257)	0.8371 (0.8253 to 0.8488)	0.8188 (0.8102 to 0.8274)	0.8338 (0.8203 to 0.8473)
114 - Retrolenticular part of internal capsule right	-0.19	0.853	0.947	0.0201 (0.0128 to 0.0275)	0.0225 (0.0133 to 0.0316)	0.8483 (0.7795 to 0.9172)	0.8352 (0.8257 to 0.8447)	0.8163 (0.8097 to 0.8228)	0.8349 (0.824 to 0.8458)
53 - Splenium of corpus callosum left	-0.18	0.859	0.947	0.0253 (0.0151 to 0.0355)	0.0277 (0.0156 to 0.0399)	1.1396 (0.8991 to 1.38)	1.0423 (1.0226 to 1.062)	1.0136 (1.0009 to 1.0262)	1.0398 (1.0224 to 1.0572)
105 - Sagittal stratum right	-0.17	0.868	0.947	0.0125 (0.0053 to 0.0197)	0.0148 (0.0066 to 0.023)	0.8527 (0.8432 to 0.8622)	0.8694 (0.861 to 0.8778)	0.8575 (0.8503 to 0.8646)	0.8683 (0.8598 to 0.8769)
34 - Posterior limb of internal capsule left	0.13	0.895	0.947	0.0083 (0.0032 to 0.0134)	0.0103 (0.0049 to 0.0157)	0.7221 (0.7145 to 0.7297)	0.7312 (0.7253 to 0.7371)	0.7224 (0.717 to 0.7278)	0.7291 (0.7225 to 0.7357)
37 - Superior corona radiata left	-0.12	0.902	0.947	0.0168 (0.0129 to 0.0207)	0.016 (0.0117 to 0.0203)	0.7562 (0.7246 to 0.7877)	0.7566 (0.7494 to 0.7639)	0.7379 (0.7323 to 0.7436)	0.7541 (0.7461 to 0.762)
93 - Anterior limb of internal capsule right	-0.07	0.941	0.964	0.0023 (-0.0043 to 0.0088)	0.0045 (-0.0029 to 0.0119)	0.7748 (0.7548 to 0.7948)	0.7642 (0.755 to 0.7733)	0.7698 (0.7619 to 0.7777)	0.7702 (0.7589 to 0.7816)
112 - Body of corpus callosum right	0	1	1	0.0399 (0.0331 to 0.0468)	0.0407 (0.0324 to 0.049)	0.9746 (0.9645 to 0.9848)	1.0142 (1.0032 to 1.0253)	0.974 (0.965 to 0.983)	1.0114 (0.9984 to 1.0244)

© 2023 Rashid T et al. *JAMA Network Open.*

Abbreviations: FDR, false discovery rate; CI, confidence interval; WM, white matter; GM, gray matter; DWM, deep white matter; SWM, superficially-located white matter; DTI, diffusion tensor imaging; FA, fractional anisotropy; MD, mean diffusivity, ROI, region-of-interest

© 2023 Rashid T et al. *JAMA Network Open*.

eTable 9. Changes in Diffusion Tensor Imaging (DTI) Mean Diffusivity (MD) Derived in Superficially-Located White Matter (SWM) Regions-of-Interest (ROIs) Between Intensive Treatment Group and Standard Treatment Group

Label – ROI Name	Effect Size	P-Value	P-Value (FDR)	Mean Change (Intensive Treatment Group) (95% CI)	Mean Change (Standard Treatment Group) (95% CI)	Mean at Baseline (Intensive Treatment Group) (95% CI)	Mean at Follow-up (Intensive Treatment Group) (95% CI)	Mean at Baseline (Standard Treatment Group) (95% CI)	Mean at Follow-up (Standard Treatment Group) (95% CI)
69 - POSTCENTRAL WM right	-2.1	0.036	0.717	0.0067 (-0.0027 to 0.0161)	-0.0009 (-0.011 to 0.0091)	0.8463 (0.8361 to 0.8565)	0.8539 (0.844 to 0.8638)	0.8552 (0.8461 to 0.8642)	0.851 (0.8405 to 0.8616)
83 - MIDDLE FRONTO-ORBITAL WM right	-1.75	0.081	0.717	0.0187 (0.0107 to 0.0267)	0.0127 (0.0057 to 0.0197)	0.7865 (0.7746 to 0.7984)	0.808 (0.7989 to 0.8171)	0.7898 (0.7828 to 0.7969)	0.8002 (0.7915 to 0.8088)
4 - MIDDLE FRONTAL WM left	-1.65	0.099	0.717	0.0104 (0.0049 to 0.0159)	0.0048 (-0.0013 to 0.0109)	0.7748 (0.7657 to 0.7838)	0.7853 (0.7752 to 0.7954)	0.7784 (0.7709 to 0.786)	0.7796 (0.7706 to 0.7886)
68 - PRECENTRAL WM right	-1.64	0.101	0.717	0.0102 (0.0059 to 0.0145)	0.0053 (0.0002 to 0.0104)	0.7582 (0.7509 to 0.7654)	0.7695 (0.763 to 0.776)	0.7629 (0.7575 to 0.7683)	0.7646 (0.758 to 0.7713)
7 - POSTCENTRAL WM left	-1.56	0.118	0.717	0.0061 (-0.0016 to 0.0137)	0.0012 (-0.0076 to 0.01)	0.8529 (0.7627 to 0.9431)	0.8135 (0.8051 to 0.8219)	0.8108 (0.8032 to 0.8185)	0.811 (0.802 to 0.8201)
73 - LINGUAL WM right	1.52	0.128	0.717	-0.0035 (-0.0495 to 0.0425)	0.0537 (-0.0056 to 0.113)	0.1408 (0.1035 to 0.1782)	0.132 (0.0896 to 0.1744)	0.1307 (0.0911 to 0.1703)	0.1718 (0.1194 to 0.2241)
64 - SUPERIOR PARIETAL WM right	-1.52	0.129	0.717	0.0163 (0.0116 to 0.0209)	0.0123 (0.0071 to 0.0176)	0.7951 (0.7871 to 0.8031)	0.8136 (0.8058 to 0.8214)	0.8008 (0.7943 to 0.8073)	0.8117 (0.8022 to 0.8213)
6 - PRECENTRAL WM left	-1.31	0.19	0.925	0.0076 (0.0034 to 0.0118)	0.0045 (-0.0008 to 0.0099)	0.7565 (0.7453 to 0.7678)	0.7587 (0.7524 to 0.7651)	0.7536 (0.7483 to 0.759)	0.7574 (0.7505 to 0.7643)
65 - SUPERIOR FRONTAL WM right	-1.19	0.235	0.951	0.0084 (0.0042 to 0.0125)	0.0056 (0.0004 to 0.0109)	0.7878 (0.7811 to 0.7946)	0.7959 (0.7902 to 0.8016)	0.7908 (0.7848 to 0.7968)	0.7923 (0.7854 to 0.7991)
3 - SUPERIOR FRONTAL WM left	-1.06	0.29	0.951	0.0096 (0.0059 to 0.0133)	0.0065 (0.0019 to 0.0111)	0.7786 (0.7721 to 0.7851)	0.7892 (0.7838 to 0.7947)	0.7828 (0.7775 to 0.788)	0.787 (0.7806 to 0.7934)
1 - SUPERIOR PARIETAL WM left	-1.03	0.301	0.951	0.015 (0.0106 to 0.0193)	0.0123 (0.0072 to 0.0174)	0.79 (0.7821 to 0.7978)	0.8062 (0.7991 to 0.8132)	0.7953 (0.7892 to 0.8013)	0.8061 (0.798 to 0.8141)
84 - SUPRAMARGINAL WM right	-1.02	0.307	0.951	0.0162 (0.0108 to 0.0216)	0.0148 (0.0085 to 0.021)	0.7999 (0.7914 to 0.8085)	0.8162 (0.8078 to 0.8245)	0.8009 (0.7951 to 0.8068)	0.8157 (0.8071 to 0.8243)

© 2023 Rashid T et al. *JAMA Network Open.*

66 - MIDDLE FRONTAL WM right	-1	0.317	0.951	0.0117 (0.0069 to 0.0166)	0.0088 (0.0031 to 0.0144)	0.7915 (0.7748 to 0.8082)	0.7947 (0.7875 to 0.8019)	0.7861 (0.779 to 0.7933)	0.7904 (0.7821 to 0.7986)
70 - ANGULAR WM right	-0.79	0.432	0.965	0.0211 (0.0165 to 0.0258)	0.0182 (0.013 to 0.0234)	0.7821 (0.7721 to 0.7921)	0.8042 (0.7935 to 0.8149)	0.7857 (0.7771 to 0.7943)	0.8021 (0.7901 to 0.8142)
21 - LATERAL FRONTO-ORBITAL WM left	-0.78	0.434	0.965	0.013 (0.0068 to 0.0192)	0.0117 (0.0057 to 0.0177)	0.7851 (0.7762 to 0.794)	0.8012 (0.7938 to 0.8087)	0.79 (0.7837 to 0.7964)	0.7986 (0.7904 to 0.8068)
10 - CUNEUS WM left	0.69	0.492	0.965	0.0101 (0.002 to 0.0181)	0.014 (0.0049 to 0.023)	0.8609 (0.7962 to 0.9255)	0.8376 (0.8258 to 0.8493)	0.8259 (0.817 to 0.8348)	0.8431 (0.8287 to 0.8575)
11 - LINGUAL WM left	0.58	0.559	0.965	-0.0081 (-0.019 to 0.0028)	-0.0046 (-0.0136 to 0.0045)	0.0089 (-0.0013 to 0.0192)	0.0044 (-0.0043 to 0.0131)	0.0061 (-0.0024 to 0.0146)	0 (0 to 0)
77 - MIDDLE OCCIPITAL WM right	-0.57	0.568	0.965	0.0104 (0.0059 to 0.0149)	0.0095 (0.0047 to 0.0142)	0.7818 (0.7624 to 0.8012)	0.7826 (0.7755 to 0.7897)	0.7776 (0.7723 to 0.7829)	0.7859 (0.7787 to 0.7931)
22 - MIDDLE FRONTO-ORBITAL WM left	-0.51	0.608	0.965	0.0074 (0.0006 to 0.0142)	0.0057 (-0.0012 to 0.0127)	0.7946 (0.7858 to 0.8034)	0.8081 (0.8013 to 0.815)	0.8014 (0.7951 to 0.8077)	0.8066 (0.799 to 0.8142)
85 - RECTUS WM right	0.48	0.629	0.965	0.0017 (-0.005 to 0.0084)	0.0053 (-0.0014 to 0.0119)	0.8149 (0.8063 to 0.8235)	0.8198 (0.8129 to 0.8267)	0.8214 (0.8145 to 0.8282)	0.822 (0.8144 to 0.8297)
19 - INFERIOR TEMPORAL WM left	0.45	0.65	0.965	0.0097 (0.0006 to 0.0188)	0.015 (0.0074 to 0.0226)	0.8162 (0.8059 to 0.8264)	0.83 (0.8229 to 0.8371)	0.8137 (0.8069 to 0.8205)	0.8295 (0.8215 to 0.8375)
16 - MIDDLE OCCIPITAL WM left	-0.36	0.721	0.965	0.0152 (0.0113 to 0.019)	0.0139 (0.0091 to 0.0187)	0.8057 (0.7418 to 0.8695)	0.7878 (0.7802 to 0.7955)	0.7771 (0.7711 to 0.7832)	0.7905 (0.7819 to 0.799)
20 - MIDDLE TEMPORAL WM left	0.35	0.726	0.965	0.007 (0.0011 to 0.0129)	0.01 (0.0044 to 0.0156)	0.7825 (0.7744 to 0.7907)	0.791 (0.785 to 0.797)	0.7853 (0.78 to 0.7906)	0.7932 (0.7864 to 0.8)
24 - RECTUS WM left	-0.35	0.726	0.965	0.0095 (-0.0075 to 0.0264)	0.0042 (-0.0116 to 0.0199)	0.8316 (0.8189 to 0.8444)	0.842 (0.8299 to 0.854)	0.8435 (0.8327 to 0.8544)	0.8424 (0.8322 to 0.8527)
9 - PRE-CUNEUS WM left	0.33	0.74	0.965	0.0089 (0.0013 to 0.0165)	0.0125 (0.0043 to 0.0208)	0.8001 (0.7906 to 0.8095)	0.8116 (0.8022 to 0.821)	0.7987 (0.7906 to 0.8067)	0.8062 (0.7971 to 0.8153)
14 - SUPERIOR OCCIPITAL WM left	0.3	0.761	0.965	0.0107 (0.0057 to 0.0157)	0.0105 (0.0051 to 0.0159)	0.7899 (0.7756 to 0.8043)	0.7935 (0.7854 to 0.8016)	0.7823 (0.7763 to 0.7884)	0.7936 (0.7853 to 0.8019)
76 - INFERIOR OCCIPITAL WM right	0.3	0.767	0.965	0.0107 (0.0059 to 0.0156)	0.0127 (0.0074 to 0.018)	0.7368 (0.7293 to 0.7443)	0.7503 (0.7447 to 0.7559)	0.7417 (0.7368 to 0.7467)	0.754 (0.7474 to 0.7606)

© 2023 Rashid T et al. *JAMA Network Open.*

23 - SUPRAMARGINAL WM left	-0.27	0.787	0.965	0.0125 (0.008 to 0.017)	0.0123 (0.0074 to 0.0172)	0.9556 (0.5795 to 1.3317)	0.7755 (0.7685 to 0.7824)	0.7671 (0.7611 to 0.773)	0.7791 (0.7717 to 0.7865)
5 - INFERIOR FRONTAL WM left	-0.25	0.802	0.965	0.0102 (0.006 to 0.0143)	0.0102 (0.0059 to 0.0145)	0.797 (0.7807 to 0.8133)	0.7991 (0.7912 to 0.807)	0.791 (0.785 to 0.797)	0.7979 (0.7904 to 0.8055)
82 - LATERAL FRONTO-ORBITAL WM right	0.22	0.828	0.965	0.0166 (0.0093 to 0.024)	0.017 (0.0103 to 0.0236)	0.7945 (0.7837 to 0.8053)	0.8115 (0.8029 to 0.82)	0.7976 (0.7911 to 0.8041)	0.812 (0.8033 to 0.8207)
74 - FUSIFORM WM right	-0.2	0.84	0.965	-0.0071 (-0.0371 to 0.0228)	-0.0133 (-0.0485 to 0.022)	0.0477 (0.0283 to 0.0672)	0.0586 (0.0329 to 0.0842)	0.0415 (0.0215 to 0.0614)	0.0439 (0.0184 to 0.0694)
75 - SUPERIOR OCCIPITAL WM right	-0.17	0.862	0.965	0.0099 (0.0051 to 0.0148)	0.0093 (0.0041 to 0.0146)	0.7917 (0.7759 to 0.8076)	0.7967 (0.7884 to 0.8051)	0.791 (0.7845 to 0.7975)	0.8013 (0.793 to 0.8096)
79 - SUPERIOR TEMPORAL WM right	-0.16	0.874	0.965	0.0127 (0.0039 to 0.0216)	0.0156 (0.0058 to 0.0254)	0.9021 (0.8202 to 0.984)	0.8743 (0.8651 to 0.8834)	0.8625 (0.8552 to 0.8698)	0.8727 (0.8633 to 0.882)
8 - ANGULAR WM left	-0.15	0.881	0.965	0.0169 (0.0127 to 0.021)	0.0165 (0.0119 to 0.0212)	0.8914 (0.6838 to 1.0989)	0.8017 (0.7932 to 0.8102)	0.7892 (0.7821 to 0.7962)	0.8062 (0.7968 to 0.8156)
15 - INFERIOR OCCIPITAL WM left	-0.14	0.886	0.965	0.0123 (0.0076 to 0.017)	0.0114 (0.0064 to 0.0163)	0.7433 (0.7349 to 0.7517)	0.7578 (0.7505 to 0.7651)	0.7507 (0.7449 to 0.7565)	0.7619 (0.7545 to 0.7693)
18 - SUPERIOR TEMPORAL WM left	-0.13	0.894	0.965	0.0064 (-0.0024 to 0.0153)	0.0097 (-0.0005 to 0.0199)	0.8581 (0.8484 to 0.8678)	0.8651 (0.8563 to 0.8738)	0.8633 (0.856 to 0.8706)	0.8694 (0.8597 to 0.8791)
80 - INFERIOR TEMPORAL WM right	0.08	0.936	0.965	0.0116 (0.0051 to 0.0181)	0.013 (0.0059 to 0.0201)	0.7979 (0.7898 to 0.8061)	0.8138 (0.806 to 0.8216)	0.804 (0.7977 to 0.8104)	0.8173 (0.8101 to 0.8245)
81 - MIDDLE TEMPORAL WM right	0.05	0.962	0.965	0.0105 (0.0045 to 0.0165)	0.0127 (0.0059 to 0.0194)	0.8196 (0.7959 to 0.8433)	0.8194 (0.8126 to 0.8262)	0.8101 (0.8046 to 0.8156)	0.8194 (0.8122 to 0.8266)
67 - INFERIOR FRONTAL WM right	-0.04	0.965	0.965	0.013 (0.0063 to 0.0197)	0.0106 (0.006 to 0.0153)	0.8241 (0.7816 to 0.8666)	0.8145 (0.8054 to 0.8235)	0.8042 (0.7974 to 0.811)	0.808 (0.8003 to 0.8157)

Abbreviations: FDR, false discovery rate; CI, confidence interval; WM, white matter; GM, gray matter; DWM, deep white matter; SWM, superficially-located white matter; DTI, diffusion tensor imaging; FA, fractional anisotropy; MD, mean diffusivity, ROI, region-of-interest

© 2023 Rashid T et al. *JAMA Network Open.*

eTable 10. Changes in Cerebral Blood Flow (CBF) Derived in Gray Matter (GM) Regions-of-Interest (ROIs) Between Intensive Treatment Group and Standard Treatment Group

Label – ROI Name	Effect Size	P-Value	P-Value (FDR)	Mean Change (Intensive Treatment Group) (95% CI)	Mean Change (Standard Treatment Group) (95% CI)	Mean at Baseline (Intensive Treatment Group) (95% CI)	Mean at Follow-up (Intensive Treatment Group) (95% CI)	Mean at Baseline (Standard Treatment Group) (95% CI)	Mean at Follow-up (Standard Treatment Group) (95% CI)
144 - Right MOG middle occipital gyrus	-2.9	0.004	0.478	8.84 (5.47 to 12.22)	1.14 (-3.72 to 6)	42.76 (40.64 to 44.89)	50.1 (46.91 to 53.29)	42.86 (39.81 to 45.9)	43.79 (40.45 to 47.13)
115 - Left Cun cuneus	-2.33	0.02	0.489	6.19 (3.83 to 8.56)	3.07 (0.42 to 5.72)	35.1 (33.48 to 36.73)	40.94 (38.53 to 43.35)	34.76 (33.04 to 36.48)	37.57 (35.25 to 39.89)
197 - Left SOG superior occipital gyrus	-2.29	0.023	0.489	6.52 (3.69 to 9.36)	0.71 (-3.97 to 5.39)	31.24 (29.18 to 33.29)	37.04 (34.36 to 39.72)	32.11 (29.08 to 35.15)	32.94 (30.26 to 35.63)
128 - Right IOG inferior occipital gyrus	-2.25	0.025	0.489	7.12 (4.11 to 10.13)	2.43 (-0.94 to 5.81)	37.92 (35.9 to 39.94)	43.96 (40.97 to 46.94)	36.52 (34.25 to 38.79)	38.45 (35.18 to 41.72)
114 - Right Cun cuneus	-2.25	0.025	0.489	6.03 (3.69 to 8.38)	2.89 (0.04 to 5.73)	36.61 (34.99 to 38.24)	42.34 (40 to 44.67)	36.29 (34.48 to 38.1)	38.6 (36.23 to 40.97)
129 - Left IOG inferior occipital gyrus	-2.16	0.032	0.489	8.02 (4.85 to 11.19)	2.64 (-1.53 to 6.82)	35.81 (33.76 to 37.87)	42.99 (40.05 to 45.93)	36.05 (33.39 to 38.72)	38.27 (35.45 to 41.08)
156 - Right OCP occipital pole	-2.14	0.033	0.489	9.46 (5.37 to 13.55)	2.39 (-2.94 to 7.73)	21.15 (18.97 to 23.34)	29.95 (26.55 to 33.36)	22.63 (19.29 to 25.97)	25.19 (21.79 to 28.59)
196 - Right SOG superior occipital gyrus	-2.11	0.035	0.489	5.47 (2.58 to 8.36)	0.06 (-4.44 to 4.56)	32.83 (30.85 to 34.81)	37.6 (34.83 to 40.37)	33.34 (30.39 to 36.3)	33.38 (30.41 to 36.35)
142 - Right MFG middle frontal gyrus	-2.04	0.042	0.489	1.27 (-0.95 to 3.49)	-1.57 (-3.93 to 0.79)	34.86 (33.27 to 36.45)	35.35 (33.24 to 37.47)	34.04 (32.47 to 35.6)	32.9 (30.68 to 35.12)
199 - Left SPL superior parietal lobule	-2.01	0.045	0.489	1.09 (-0.85 to 3.02)	-2.17 (-5.15 to 0.81)	31.42 (29.88 to 32.96)	32.37 (30.25 to 34.49)	30.72 (28.78 to 32.65)	28.91 (26.85 to 30.97)
183 - Left PrG precentral gyrus	-2	0.046	0.489	1.79 (-0.32 to 3.9)	-0.86 (-3.38 to 1.66)	37.55 (36.08 to 39.03)	39.08 (37 to 41.15)	37.43 (35.86 to 38.99)	36.94 (34.93 to 38.94)
194 - Right SMG supramarginal gyrus	-1.98	0.049	0.489	5.85 (3.11 to 8.6)	2.86 (-0.22 to 5.94)	39.95 (38.38 to 41.52)	44.8 (42.28 to 47.32)	38.48 (36.86 to 40.11)	41.45 (38.88 to 44.02)
182 - Right PrG precentral gyrus	-1.92	0.056	0.503	2.54 (0.36 to 4.72)	0.18 (-2.24 to 2.6)	37.72 (36.21 to 39.22)	39.5 (37.47 to 41.53)	36.49 (35.03 to 37.95)	37.02 (34.83 to 39.22)
191 - Left SFG superior frontal gyrus	-1.85	0.066	0.503	1.91 (0.16 to 3.66)	-0.11 (-2.01 to 1.78)	27.34 (26.06 to 28.61)	29.13 (27.45 to 30.82)	27.65 (26.39 to 28.9)	27.97 (26.26 to 29.67)
145 - Left MOG middle occipital gyrus	-1.81	0.071	0.503	8.49 (5.02 to 11.96)	2.58 (-3.16 to 8.32)	40.74 (38.51 to 42.98)	48.15 (45.12 to 51.18)	41.22 (37.64 to 44.8)	43.56 (40.52 to 46.61)
169 - Left PCu precuneus	-1.79	0.074	0.503	4.08 (1.64 to 6.53)	1.93 (-0.77 to 4.64)	42.3 (40.63 to 43.97)	46.1 (43.69 to 48.51)	40.91 (39.28 to 42.54)	42.59 (40.29 to 44.88)
143 - Left MFG middle frontal gyrus	-1.76	0.078	0.503	1.31 (-0.88 to 3.5)	-1.23 (-3.76 to 1.31)	33.89 (32.32 to 35.46)	35.08 (32.87 to 37.3)	33.87 (32.2 to 35.53)	33.22 (31.15 to 35.28)
107 - Left AnG angular gyrus	-1.72	0.086	0.503	7.41 (4.58 to 10.23)	3.81 (-0.48 to 8.1)	43.14 (41.39 to 44.88)	49.88 (47.26 to 52.51)	42.32 (39.84 to 44.8)	46.12 (43.68 to 48.56)

© 2023 Rashid T et al. *JAMA Network Open.*

176 - Right PoG postcentral gyrus	-1.72	0.087	0.503	1.89 (-0.21 to 3.99)	-0.24 (-2.56 to 2.08)	34.37 (32.95 to 35.79)	35.47 (33.51 to 37.44)	33.14 (31.76 to 34.52)	33.3 (31.31 to 35.29)
109 - Left Calc calcarine cortex	-1.69	0.091	0.503	8.35 (5.46 to 11.24)	6.21 (2.82 to 9.6)	44.85 (42.98 to 46.72)	52.97 (50.21 to 55.73)	44.34 (42.45 to 46.24)	50.32 (47.48 to 53.16)
204 - Right TrIFG triangular part of the inferior frontal gyrus	-1.68	0.094	0.503	3.83 (0.3 to 7.36)	0.76 (-2.69 to 4.21)	40.65 (38.85 to 42.45)	43.36 (40.26 to 46.46)	39.41 (37.39 to 41.42)	40 (37.03 to 42.96)
106 - Right AnG angular gyrus	-1.67	0.095	0.503	6.34 (3.4 to 9.27)	3.31 (-0.46 to 7.07)	45.46 (43.64 to 47.29)	50.58 (47.87 to 53.29)	43.73 (41.61 to 45.85)	46.72 (43.94 to 49.49)
118 - Right FO frontal operculum	-1.67	0.096	0.503	9.21 (5.54 to 12.88)	6.57 (3.18 to 9.96)	42.67 (41.16 to 44.19)	51.51 (48.12 to 54.89)	41.62 (40.13 to 43.1)	47.91 (44.69 to 51.13)
39 - Left Cerebellum Exterior	-1.63	0.104	0.506	8.86 (6.3 to 11.42)	7.01 (4.02 to 10)	29.69 (28.27 to 31.12)	38.33 (35.97 to 40.69)	30.3 (29.01 to 31.58)	37.44 (34.8 to 40.08)
162 - Right OpIFG opercular part of the inferior frontal gyrus	-1.6	0.11	0.506	6.14 (3.06 to 9.22)	4.03 (0.83 to 7.22)	43.21 (41.58 to 44.85)	48.75 (45.98 to 51.53)	42.17 (40.46 to 43.88)	45.87 (43.06 to 48.69)
190 - Right SFG superior frontal gyrus	-1.6	0.11	0.506	1.83 (0.06 to 3.6)	-0.08 (-2.08 to 1.92)	27.07 (25.8 to 28.34)	28.55 (26.86 to 30.23)	27.13 (25.78 to 28.47)	27.33 (25.56 to 29.11)
157 - Left OCP occipital pole	-1.52	0.13	0.575	9.18 (4.69 to 13.67)	3.5 (-2.14 to 9.14)	22.12 (19.77 to 24.48)	31.2 (27.51 to 34.89)	23.09 (19.54 to 26.65)	27.11 (23.93 to 30.28)
56 - Left Pallidum	-1.5	0.134	0.575	7.87 (5.44 to 10.29)	6.19 (3.67 to 8.7)	34.17 (33.07 to 35.28)	41.58 (39.34 to 43.81)	33.97 (32.89 to 35.06)	39.99 (37.74 to 42.25)
36 - Right Caudate	-1.43	0.155	0.64	2.99 (1.18 to 4.8)	1.43 (-0.25 to 3.1)	25.6 (24.46 to 26.74)	28.3 (26.58 to 30.03)	25.22 (24.19 to 26.25)	26.73 (25.07 to 28.4)
108 - Right Calc calcarine cortex	-1.39	0.166	0.654	8.19 (5.14 to 11.25)	6.55 (3.24 to 9.86)	45.9 (44.03 to 47.76)	53.55 (50.67 to 56.44)	45.29 (43.42 to 47.16)	51.32 (48.48 to 54.16)
198 - Right SPL superior parietal lobule	-1.38	0.169	0.654	-0.16 (-2.08 to 1.75)	-2.2 (-5.06 to 0.67)	31.04 (29.52 to 32.57)	30.57 (28.49 to 32.65)	30.03 (28.14 to 31.93)	28.25 (26.19 to 30.32)
168 - Right PCu precuneus	-1.36	0.175	0.657	3.88 (1.39 to 6.36)	2.42 (-0.26 to 5.1)	42.54 (40.91 to 44.18)	46.24 (43.83 to 48.64)	41.28 (39.68 to 42.88)	43.36 (41.11 to 45.61)
164 - Right OrIFG orbital part of the inferior frontal gyrus	-1.32	0.188	0.678	5.03 (1.31 to 8.74)	2.58 (-1.51 to 6.66)	40.1 (38.3 to 41.9)	44.62 (41.47 to 47.77)	39.59 (37.45 to 41.74)	42.64 (39.39 to 45.9)
177 - Left PoG postcentral gyrus	-1.28	0.203	0.678	1.26 (-0.82 to 3.34)	-0.69 (-3.66 to 2.28)	33.83 (32.45 to 35.22)	34.84 (32.75 to 36.93)	33.61 (31.79 to 35.43)	33.41 (31.51 to 35.32)
161 - Left OFuG occipital fusiform gyrus	-1.27	0.205	0.678	8.41 (5.5 to 11.32)	6.6 (3.48 to 9.73)	33.58 (31.87 to 35.29)	41.7 (38.94 to 44.46)	33.73 (32.03 to 35.42)	40.19 (37.4 to 42.98)
58 - Left Putamen	-1.25	0.211	0.678	7.51 (4.9 to 10.11)	5.79 (3.08 to 8.5)	38.94 (37.7 to 40.18)	45.67 (43.26 to 48.09)	38.46 (37.33 to 39.59)	44.02 (41.56 to 46.48)
175 - Left PO parietal operculum	-1.25	0.212	0.678	8.88 (5.27 to 12.49)	7.15 (3.26 to 11.04)	47.55 (46.05 to 49.06)	56.5 (53.13 to 59.87)	46.66 (45.2 to 48.12)	53.89 (50.5 to 57.29)
112 - Right CO central operculum	-1.24	0.215	0.678	9.21 (5.67 to 12.75)	7.63 (3.82 to 11.43)	43.53 (42.08 to 44.98)	52.4 (49.09 to 55.71)	42.27 (40.85 to 43.69)	49.57 (46.08 to 53.05)
195 - Left SMG supramarginal gyrus	-1.19	0.236	0.722	5.59 (2.92 to 8.27)	3.43 (-0.5 to 7.35)	39.88 (38.32 to 41.43)	45.28 (42.69 to 47.87)	39.15 (36.97 to 41.34)	42.76 (40.4 to 45.13)
150 - Right MPrG precentral gyrus medial segment	-1.17	0.244	0.722	4.56 (2.12 to 6.99)	3.18 (0.52 to 5.84)	40.48 (38.87 to 42.08)	44.43 (42.25 to 46.61)	39.08 (37.59 to 40.56)	42.01 (39.68 to 44.34)

© 2023 Rashid T et al. *JAMA Network Open.*

151 - Left MPrG precentral gyrus medial segment	-1.16	0.247	0.722	3.6 (1.26 to 5.95)	2.3 (-0.33 to 4.92)	40.09 (38.49 to 41.7)	43.25 (41.14 to 45.37)	39.02 (37.53 to 40.5)	41.18 (38.93 to 43.43)
148 - Right MPoG postcentral gyrus medial segment	-1.13	0.261	0.729	0.83 (-1.32 to 2.98)	-0.66 (-3.29 to 1.96)	32.4 (30.82 to 33.98)	32.94 (30.94 to 34.94)	30.9 (29.19 to 32.62)	30.75 (28.76 to 32.74)
192 - Right SMC supplementary motor cortex	-1.12	0.261	0.729	5.01 (2.44 to 7.57)	3.67 (1.16 to 6.18)	38.76 (37.21 to 40.3)	43.23 (40.96 to 45.5)	37.5 (36.08 to 38.91)	40.97 (38.7 to 43.24)
135 - Left LiG lingual gyrus	-1.11	0.267	0.729	9.31 (6.38 to 12.23)	8.31 (5.21 to 11.41)	39.36 (37.78 to 40.93)	48.25 (45.57 to 50.93)	38.74 (37.25 to 40.24)	46.51 (43.94 to 49.07)
174 - Right PO parietal operculum	-1.07	0.287	0.766	7.9 (4.49 to 11.31)	6.81 (3.37 to 10.26)	46.31 (44.78 to 47.83)	54.03 (50.89 to 57.18)	44.67 (43.2 to 46.14)	51.41 (48.31 to 54.51)
57 - Right Putamen	-0.98	0.325	0.819	7.33 (4.74 to 9.92)	6.08 (3.56 to 8.61)	38.79 (37.52 to 40.06)	45.58 (43.15 to 48)	38.24 (37.11 to 39.37)	44.26 (41.94 to 46.58)
160 - Right OFuG occipital fusiform gyrus	-0.98	0.33	0.819	8.1 (5.45 to 10.74)	7.07 (4.05 to 10.08)	35.38 (33.7 to 37.07)	42.87 (40.33 to 45.41)	34.45 (32.76 to 36.15)	41.37 (38.64 to 44.11)
113 - Left CO central operculum	-0.97	0.335	0.819	8.89 (5.44 to 12.34)	7.65 (3.7 to 11.6)	43.04 (41.66 to 44.42)	51.76 (48.51 to 55)	42.11 (40.78 to 43.43)	49.68 (46.01 to 53.34)
154 - Right MTG middle temporal gyrus	-0.96	0.338	0.819	4.81 (2.02 to 7.61)	3.8 (0.82 to 6.78)	44.48 (42.78 to 46.18)	48.14 (45.62 to 50.67)	43.46 (41.83 to 45.08)	46.53 (43.84 to 49.23)
134 - Right LiG lingual gyrus	-0.93	0.352	0.819	8.62 (5.73 to 11.51)	8.02 (5.18 to 10.87)	38.92 (37.37 to 40.48)	46.89 (44.33 to 49.44)	38.17 (36.71 to 39.62)	45.52 (43.08 to 47.96)
35 - Brain Stem	-0.93	0.352	0.819	7.23 (4.82 to 9.64)	6.84 (4.27 to 9.4)	27.95 (26.75 to 29.14)	34.61 (32.5 to 36.73)	27.11 (25.93 to 28.3)	33.61 (31.58 to 35.64)
138 - Right MCgG middle cingulate gyrus	-0.93	0.355	0.819	9.49 (5.61 to 13.37)	8.17 (4.22 to 12.11)	48.55 (46.93 to 50.18)	57.47 (53.85 to 61.09)	47.14 (45.63 to 48.65)	55.09 (51.5 to 58.68)
153 - Left MSFG superior frontal gyrus medial segment	-0.89	0.372	0.842	4.28 (2.01 to 6.55)	3.38 (1.04 to 5.71)	33.58 (32.22 to 34.93)	37.48 (35.27 to 39.69)	32.71 (31.41 to 34.01)	36.05 (33.74 to 38.35)
101 - Left ACgG anterior cingulate gyrus	-0.85	0.394	0.876	8.44 (4.82 to 12.06)	7.28 (3.77 to 10.79)	41.15 (39.76 to 42.54)	49.05 (45.6 to 52.49)	40.55 (39.12 to 41.97)	47.8 (44.49 to 51.11)
119 - Left FO frontal operculum	-0.81	0.417	0.908	7.93 (4.53 to 11.33)	6.79 (3.24 to 10.33)	41.75 (40.33 to 43.16)	49.38 (46.18 to 52.57)	40.8 (39.4 to 42.2)	47.39 (44.09 to 50.69)
149 - Left MPoG postcentral gyrus medial segment	-0.77	0.439	0.908	0.74 (-1.29 to 2.77)	-0.18 (-2.57 to 2.21)	31.45 (29.91 to 32.99)	31.63 (29.75 to 33.5)	30.79 (29.17 to 32.41)	30.94 (28.95 to 32.93)
193 - Left SMC supplementary motor cortex	-0.77	0.44	0.908	4.89 (2.28 to 7.5)	4.05 (1.54 to 6.57)	39.04 (37.56 to 40.52)	43.58 (41.23 to 45.93)	38.15 (36.8 to 39.49)	41.82 (39.52 to 44.11)
152 - Right MSFG superior frontal gyrus medial segment	-0.75	0.456	0.908	4.86 (2.61 to 7.11)	3.97 (1.52 to 6.42)	33.72 (32.35 to 35.08)	37.94 (35.79 to 40.08)	33.33 (32.05 to 34.62)	37.36 (35.07 to 39.66)
30 - Left Accumbens Area	0.74	0.463	0.908	2.97 (0.17 to 5.78)	4.84 (0.68 to 9.01)	34.94 (33.34 to 36.54)	37.85 (35.43 to 40.27)	33.87 (31.27 to 36.47)	37.97 (35.84 to 40.1)
173 - Left PIns posterior insula	-0.73	0.465	0.908	11.74 (6.79 to 16.7)	10.51 (5.63 to 15.39)	41.12 (39.78 to 42.46)	52.7 (47.98 to 57.43)	40.27 (39.02 to 41.52)	50.57 (46.02 to 55.13)
103 - Left AIns anterior insula	-0.71	0.479	0.908	10.01 (5.9 to 14.12)	8.92 (4.69 to 13.15)	41.27 (39.95 to 42.59)	50.91 (46.96 to 54.85)	40.22 (38.95 to 41.48)	48.88 (44.91 to 52.85)
76 - Right Basal Forebrain	0.69	0.489	0.908	8.7 (4.46 to 12.95)	10.93 (5.79 to 16.07)	33.73 (32.24 to 35.21)	42.77 (38.84 to 46.7)	32.62 (30.5 to 34.74)	43.5 (39.09 to 47.92)

© 2023 Rashid T et al. *JAMA Network Open.*

163 - Left OpIFG opercular part of the inferior frontal gyrus	-0.67	0.502	0.908	5.56 (2.62 to 8.5)	4.89 (1.84 to 7.95)	41.51 (39.95 to 43.07)	46.97 (44.11 to 49.84)	41.39 (39.93 to 42.85)	46.1 (43.28 to 48.92)
206 - Right TTG transverse temporal gyrus	-0.66	0.509	0.908	13.86 (8.64 to 19.09)	12.89 (7.05 to 18.74)	53.66 (51.93 to 55.39)	67.24 (62.4 to 72.08)	52.75 (51.09 to 54.41)	65.16 (59.73 to 70.59)
55 - Right Pallidum	-0.66	0.51	0.908	7.67 (5.28 to 10.06)	7.07 (4.63 to 9.52)	33.62 (32.52 to 34.72)	40.89 (38.7 to 43.08)	33.39 (32.3 to 34.48)	40.37 (38.2 to 42.55)
133 - Left ITG inferior temporal gyrus	0.6	0.547	0.908	0.37 (-2.32 to 3.07)	1.72 (-0.76 to 4.21)	27.41 (25.76 to 29.06)	28.26 (25.91 to 30.61)	26.84 (25.16 to 28.52)	28.51 (26.34 to 30.68)
203 - Left TMP temporal pole	0.6	0.551	0.908	0.59 (-1.79 to 2.97)	2.07 (-0.58 to 4.72)	24.76 (23.6 to 25.91)	25.45 (23.42 to 27.48)	24.33 (22.98 to 25.67)	26.95 (24.91 to 28.99)
139 - Left MCgG middle cingulate gyrus	-0.58	0.559	0.908	8.9 (5.05 to 12.75)	8.39 (4.45 to 12.32)	47.93 (46.38 to 49.48)	56.52 (52.95 to 60.1)	46.59 (45.14 to 48.04)	54.62 (51.07 to 58.17)
105 - Left AOrG anterior orbital gyrus	-0.58	0.56	0.908	-1.37 (-5.15 to 2.41)	-2.41 (-6.79 to 1.97)	34.53 (32.29 to 36.78)	33.56 (30.36 to 36.77)	35.2 (32.53 to 37.88)	34.59 (31.76 to 37.41)
104 - Right AOrG anterior orbital gyrus	-0.56	0.577	0.908	0.05 (-3.93 to 4.03)	-1.06 (-5.39 to 3.28)	34.73 (32.56 to 36.91)	34.83 (31.51 to 38.16)	34.87 (32.3 to 37.44)	35.56 (32.64 to 38.48)
132 - Right ITG inferior temporal gyrus	-0.56	0.579	0.908	2.13 (-0.41 to 4.68)	1.36 (-1.72 to 4.43)	28.25 (26.55 to 29.94)	29.81 (27.56 to 32.06)	27.97 (26.1 to 29.85)	29.25 (26.77 to 31.74)
205 - Left TrIFG triangular part of the inferior frontal gyrus	-0.55	0.58	0.908	2.84 (-0.26 to 5.93)	2.5 (-0.53 to 5.53)	37.73 (35.94 to 39.52)	40.97 (38.03 to 43.91)	37.69 (36.09 to 39.29)	40.36 (37.57 to 43.15)
137 - Left LOrG lateral orbital gyrus	-0.54	0.587	0.908	-0.75 (-4.23 to 2.73)	-1.46 (-5.91 to 2.98)	33.32 (31.16 to 35.48)	33 (30.05 to 35.96)	33.98 (31.38 to 36.57)	34.01 (31.06 to 36.95)
136 - Right LOrG lateral orbital gyrus	-0.54	0.589	0.908	0.38 (-3.48 to 4.23)	-0.46 (-5.19 to 4.27)	33.67 (31.64 to 35.69)	33.87 (30.59 to 37.16)	33.96 (31.25 to 36.68)	35.07 (32.18 to 37.97)
60 - Left Thalamus Proper	-0.53	0.596	0.908	4.4 (1.97 to 6.83)	4.15 (1.75 to 6.56)	36.88 (35.42 to 38.35)	40.89 (38.67 to 43.11)	35.64 (34.19 to 37.09)	39.63 (37.54 to 41.72)
31 - Right Amygdala	-0.52	0.6	0.908	6.99 (3.84 to 10.14)	6.52 (3.36 to 9.68)	31.81 (30.62 to 33.01)	38.83 (35.8 to 41.86)	31.57 (30.34 to 32.8)	38.01 (35.12 to 40.9)
172 - Right PIns posterior insula	-0.51	0.609	0.908	10.72 (6.24 to 15.2)	10.43 (5.77 to 15.08)	40.9 (39.55 to 42.26)	51.51 (47.22 to 55.8)	39.97 (38.7 to 41.24)	50.14 (45.78 to 54.51)
200 - Right STG superior temporal gyrus	-0.51	0.612	0.908	7.14 (4.02 to 10.25)	7.12 (3.69 to 10.55)	46.67 (45.09 to 48.25)	52.94 (50.19 to 55.68)	45.58 (43.96 to 47.21)	51.81 (48.86 to 54.76)
73 - Cerebellar Vermal Lobules VIII-X	0.51	0.613	0.908	8.4 (4.14 to 12.66)	10.49 (5.26 to 15.72)	35.49 (33.5 to 37.48)	42.61 (38.87 to 46.35)	34.58 (32.44 to 36.71)	46.53 (41.92 to 51.14)
181 - Left PP planum polare	-0.5	0.615	0.908	11.39 (6.41 to 16.38)	10.88 (6.3 to 15.45)	39.42 (38.03 to 40.81)	50.83 (46.13 to 55.52)	39.26 (37.9 to 40.62)	49.69 (45.42 to 53.95)
102 - Right AIns anterior insula	-0.5	0.615	0.908	10.16 (6.08 to 14.24)	9.75 (5.5 to 14.01)	41.86 (40.5 to 43.21)	51.61 (47.68 to 55.55)	40.81 (39.52 to 42.1)	50.55 (46.46 to 54.64)
184 - Right PT planum temporale	-0.5	0.62	0.908	10.85 (6.44 to 15.25)	10.79 (6.1 to 15.48)	53.81 (52 to 55.61)	64.02 (60.03 to 68.01)	52.08 (50.3 to 53.86)	61.88 (57.66 to 66.1)
100 - Right ACgG anterior cingulate gyrus	-0.48	0.635	0.918	8.56 (4.78 to 12.34)	7.76 (3.84 to 11.67)	41.39 (39.92 to 42.87)	49.25 (45.69 to 52.8)	40.51 (39.07 to 41.95)	48.32 (44.63 to 52.01)
179 - Left POrG posterior orbital gyrus	-0.4	0.69	0.967	3.04 (0.1 to 5.98)	2.82 (-1.16 to 6.8)	35.26 (33.64 to 36.88)	38.24 (35.66 to 40.83)	35.17 (32.9 to 37.44)	38.25 (35.77 to 40.73)

© 2023 Rashid T et al. *JAMA Network Open.*

178 - Right POrG posterior orbital gyrus	0.4	0.692	0.967	2.89 (-0.01 to 5.79)	4.4 (0.52 to 8.29)	35.05 (33.4 to 36.7)	37.87 (35.32 to 40.41)	34.52 (32.23 to 36.82)	39.08 (36.57 to 41.6)
171 - Left PHG parahippocampal gyrus	0.38	0.703	0.967	7.2 (4.24 to 10.16)	8.84 (5.55 to 12.12)	31.36 (30.12 to 32.6)	38.27 (35.64 to 40.9)	30.13 (28.61 to 31.66)	38.78 (36.04 to 41.52)
121 - Left FRP frontal pole	0.35	0.728	0.967	-2.26 (-4.28 to -0.25)	-1.38 (-3.99 to 1.24)	16.41 (15.03 to 17.78)	14.31 (12.64 to 15.97)	16.48 (14.87 to 18.09)	15.8 (14.01 to 17.6)
23 - Right Accumbens Area	0.34	0.731	0.967	3.73 (0.8 to 6.66)	4.56 (1.11 to 8.01)	33.87 (32.35 to 35.39)	37.23 (34.5 to 39.96)	32.81 (30.72 to 34.89)	36.97 (34.67 to 39.27)
59 - Right Thalamus Proper	-0.33	0.742	0.967	4.21 (1.76 to 6.67)	4.24 (1.83 to 6.66)	37.7 (36.18 to 39.22)	41.44 (39.33 to 43.55)	36.41 (34.91 to 37.9)	40.26 (38.14 to 42.39)
186 - Right SCA subcallosal area	0.33	0.742	0.967	3.11 (-0.53 to 6.75)	3.77 (-0.78 to 8.32)	30.82 (28.97 to 32.67)	34.15 (31.01 to 37.28)	29.71 (27.09 to 32.32)	33.52 (30.78 to 36.27)
37 - Left Caudate	0.32	0.75	0.967	1.94 (0.09 to 3.79)	2.51 (0.88 to 4.14)	26.17 (25.04 to 27.3)	28.11 (26.43 to 29.8)	25.67 (24.59 to 26.74)	27.88 (26.3 to 29.45)
38 - Right Cerebellum Exterior	-0.31	0.758	0.967	7.61 (5.14 to 10.08)	7.74 (4.73 to 10.74)	30.56 (29.21 to 31.92)	37.93 (35.65 to 40.21)	30.19 (28.84 to 31.53)	37.79 (35.08 to 40.49)
202 - Right TMP temporal pole	0.29	0.769	0.967	1.47 (-0.8 to 3.74)	2.51 (-0.26 to 5.28)	25.67 (24.48 to 26.87)	27.05 (25.05 to 29.04)	24.99 (23.44 to 26.53)	27.86 (25.81 to 29.91)
122 - Right FuG fusiform gyrus	0.27	0.785	0.967	5.7 (3.15 to 8.25)	6.91 (4.05 to 9.77)	33.38 (31.98 to 34.78)	38.84 (36.69 to 40.99)	32.6 (31.14 to 34.06)	39.15 (36.72 to 41.59)
147 - Left MOrG medial orbital gyrus	0.27	0.789	0.967	-0.22 (-4.16 to 3.72)	0.86 (-4.16 to 5.88)	30.08 (27.84 to 32.31)	30.43 (27.09 to 33.76)	30.19 (27.15 to 33.22)	32.14 (29.37 to 34.91)
170 - Right PHG parahippocampal gyrus	0.26	0.798	0.967	7.59 (4.61 to 10.57)	9.07 (5.71 to 12.43)	30.26 (28.93 to 31.59)	37.73 (35.04 to 40.43)	30.01 (28.61 to 31.4)	38.93 (35.95 to 41.9)
180 - Right PP planum polare	-0.25	0.799	0.967	11.43 (6.78 to 16.09)	11.49 (6.31 to 16.67)	40.44 (38.94 to 41.94)	51.6 (47.2 to 55.99)	39.87 (38.41 to 41.33)	50.87 (45.94 to 55.8)
75 - Left Basal Forebrain	0.25	0.8	0.967	7.69 (3.79 to 11.59)	8.96 (4.23 to 13.69)	33.61 (32.2 to 35.02)	41.22 (37.62 to 44.82)	32.07 (29.79 to 34.35)	41.02 (37.53 to 44.5)
72 - Cerebellar Vermal Lobules VI-VII	-0.25	0.805	0.967	4.85 (2.5 to 7.2)	4.94 (1.98 to 7.9)	27.21 (25.65 to 28.76)	31.58 (29.23 to 33.93)	26.71 (25.1 to 28.32)	31.33 (28.69 to 33.97)
165 - Left OrIFG orbital part of the inferior frontal gyrus	0.23	0.815	0.967	3.19 (0.22 to 6.16)	4.26 (0.86 to 7.67)	37.67 (36.03 to 39.31)	41.33 (38.71 to 43.94)	37.18 (35.5 to 38.85)	41.84 (38.9 to 44.78)
48 - Left Hippocampus	-0.23	0.818	0.967	6.86 (4.12 to 9.61)	7.29 (4.27 to 10.31)	38.28 (36.97 to 39.59)	44.77 (42.32 to 47.21)	37.34 (36.03 to 38.65)	44.32 (41.75 to 46.88)
32 - Left Amygdala	-0.22	0.828	0.967	7.32 (4.02 to 10.63)	7.68 (3.56 to 11.81)	32.25 (31.03 to 33.46)	39.17 (36.14 to 42.2)	31.41 (30.09 to 32.74)	38.83 (35.12 to 42.53)
125 - Left GRe gyrus rectus	0.21	0.83	0.967	0.7 (-3.56 to 4.96)	1.76 (-3.27 to 6.79)	30.64 (28.26 to 33.02)	31.65 (28.18 to 35.13)	31.35 (28.47 to 34.22)	35.04 (31.76 to 38.33)
123 - Left FuG fusiform gyrus	0.19	0.852	0.981	5.22 (2.61 to 7.82)	6.4 (3.61 to 9.18)	33.37 (31.94 to 34.79)	38.58 (36.29 to 40.87)	32.26 (30.82 to 33.7)	38.61 (36.3 to 40.91)
146 - Right MOrG medial orbital gyrus	0.16	0.872	0.981	0.25 (-3.81 to 4.31)	0.9 (-4.66 to 6.45)	30.28 (28.03 to 32.53)	30.86 (27.48 to 34.24)	29.89 (26.63 to 33.15)	31.62 (28.69 to 34.55)
124 - Right GRe gyrus rectus	0.16	0.875	0.981	0.62 (-3.92 to 5.17)	1.23 (-4.24 to 6.7)	30.64 (28.15 to 33.13)	31.43 (27.64 to 35.21)	31.59 (28.5 to 34.68)	34.4 (31.17 to 37.62)

© 2023 Rashid T et al. *JAMA Network Open.*

117 - Left Ent entorhinal area	0.15	0.883	0.981	2.13 (-0.79 to 5.04)	3.08 (-0.47 to 6.63)	25.11 (23.88 to 26.35)	27.27 (24.6 to 29.94)	24.11 (22.54 to 25.68)	27.34 (24.24 to 30.43)
155 - Left MTG middle temporal gyrus	0.15	0.884	0.981	3.85 (0.86 to 6.84)	4.52 (1.54 to 7.49)	42.07 (40.45 to 43.7)	45.88 (43.2 to 48.57)	41.86 (40.21 to 43.52)	45.98 (43.61 to 48.35)
120 - Right FRP frontal pole	-0.14	0.891	0.981	-0.94 (-2.92 to 1.03)	-0.85 (-3.46 to 1.77)	15.99 (14.68 to 17.29)	15.25 (13.47 to 17.02)	16.43 (14.88 to 17.98)	16.64 (14.68 to 18.6)
140 - Right MFC medial frontal cortex	0.11	0.913	0.987	3.86 (-0.18 to 7.91)	4.4 (0.34 to 8.46)	41.65 (39.66 to 43.63)	45.34 (41.86 to 48.81)	41.86 (39.68 to 44.03)	47.27 (44.1 to 50.44)
166 - Right PCgG posterior cingulate gyrus	-0.11	0.916	0.987	4.73 (1.76 to 7.7)	5.21 (1.98 to 8.44)	51.27 (49.53 to 53.01)	55.68 (53 to 58.37)	49.29 (47.62 to 50.96)	54.22 (51.51 to 56.94)
116 - Right Ent entorhinal area	0.1	0.922	0.987	3.92 (0.85 to 6.98)	4.77 (1.85 to 7.69)	25.09 (23.81 to 26.36)	29.54 (26.59 to 32.49)	24.11 (22.76 to 25.46)	29.28 (26.64 to 31.93)
207 - Left TTG transverse temporal gyrus	-0.08	0.939	0.992	12.75 (7.55 to 17.95)	13.5 (7.22 to 19.77)	54.96 (53.32 to 56.6)	67.79 (62.96 to 72.62)	53.97 (52.32 to 55.62)	67.08 (61.31 to 72.85)
167 - Left PCgG posterior cingulate gyrus	-0.07	0.944	0.992	4.52 (1.56 to 7.48)	5.13 (1.87 to 8.39)	50.62 (48.87 to 52.38)	54.94 (52.2 to 57.67)	48.79 (47.13 to 50.44)	53.75 (51.02 to 56.48)
185 - Left PT planum temporale	-0.06	0.951	0.992	9.71 (5.66 to 13.76)	10.44 (5.8 to 15.09)	52.09 (50.43 to 53.75)	61.82 (58.17 to 65.46)	51.86 (50.22 to 53.51)	61.76 (57.8 to 65.73)
187 - Left SCA subcallosal area	-0.04	0.969	0.997	3.39 (-0.39 to 7.16)	3.14 (-0.79 to 7.07)	30.99 (29.19 to 32.79)	34.03 (30.75 to 37.3)	31.29 (29.03 to 33.56)	34.56 (31.75 to 37.37)
201 - Left STG superior temporal gyrus	0.03	0.972	0.997	6.37 (3.23 to 9.51)	7.05 (3.66 to 10.44)	44.25 (42.75 to 45.76)	50.59 (47.74 to 53.45)	43.92 (42.37 to 45.47)	50.8 (48.12 to 53.48)
71 - Cerebellar Vermal Lobules I-V	-0.01	0.989	0.999	5.69 (3.44 to 7.95)	6.3 (3.95 to 8.65)	31.26 (29.9 to 32.63)	36.76 (34.61 to 38.9)	30.24 (28.81 to 31.67)	35.68 (33.77 to 37.6)
141 - Left MFC medial frontal cortex	0	0.997	0.999	3.51 (-0.27 to 7.29)	3.99 (-0.03 to 8.01)	41.21 (39.31 to 43.11)	44.61 (41.37 to 47.86)	41.3 (39.05 to 43.55)	46.12 (43.16 to 49.07)
47 - Right Hippocampus	0	0.999	0.999	6.31 (3.72 to 8.91)	7.2 (4.26 to 10.14)	37.62 (36.32 to 38.92)	43.63 (41.36 to 45.9)	37.46 (36.18 to 38.75)	44.33 (41.7 to 46.96)

Abbreviations: FDR, false discovery rate; CI, confidence interval; WM, white matter; GM, gray matter; DWM, deep white matter; SWM, superficially-located white matter; DTI, diffusion tensor imaging; FA, fractional anisotropy; MD, mean diffusivity, ROI, region-of-interest

© 2023 Rashid T et al. *JAMA Network Open.*

eMethods 2. Multiple Imputation

We conducted sensitivity analysis using multiple imputation (MI) to assess the influence of missing data from participants who did not complete follow-up MRI for white matter lesions. This analysis entails the common missing-at-random assumption conditional on the variables used in the MI model[20]. The base set of predictors were all baseline variables that had no missing values. These were age, sex, race/ethnicity (Black, Hispanic, White and Other), education (Less than high school education, High school graduate, Post high school and College graduate), History of cardiovascular disease (CVD), systolic and diastolic BP, whether the participant had measured orthostatic hypotension, smoking status (Former smoker, Current smoker, Never smoked) and alcohol consumption (Heavy-drinker, Moderate-drinker, Light-drinker and Non-drinker). These base set of variables were used to impute missing data for other baseline variables such as Body Mass Index (BMI), HDL cholesterol, Serum bicarbonate (CO2), estimated glomerular filtration rate (eGFR), Log Urine Albumin to Creatinine Ratio (log UACR), MoCA score, Logical Memory form II (LM), Digital Symbol Coding score (DCS), VR-12 Physical Component Summary Score (VR-12 PCS), VR-12 Mental Component Summary (VR-12 MCS) and PHQ-9 score ≥ 10 (Yes or No) based on the conditional specification shown in Supplementary Table 11.

Using the imputed dataset from the step above, we then imputed the missing follow-up WML values ofr deep white matter ROIs separately by treatment group as a function of: age, sex, race/ethnicity, education, smoking status, history of CVD, SBP, DBP, BMI, HDL cholesterol, serum bicarbonate, eGFR, log-transformed urine albumin-to-creatinine ratio, scores from MoCA, LM, DSC, VR12 components and the baseline value of the ROI of interest, intracranial volume and days since randomization (set to 1452 days). Note that we did not impute follow-up WML outcomes for participants who died prior to the timing of the follow-up scan. We then fit linear mixed models to each imputed dataset, with participant and MRI facility as random effects. The imputation process was implemented with the Multivariate Imputation by Chained Equations (MICE) package in R (R version 3.6.3).

© 2023 Rashid T et al. *JAMA Network Open.*

eTable 11. Condition Model for Multiple Imputation

Auxiliary Variable	Imputation Model	Predictors
Body Mass Index (BMI)	Linear	Base Set
HDL Cholesterol (HDL)	Linear	Base Set + BMI
Serum Bicarbonate (CO2)	Linear	Base Set + BMI + HDL
eGFR	Linear	Base Set + BMI + HDL + CO2
Log Urine Albumin to Creatinine Ratio (log UACR)	Linear	Base Set + BMI + HDL + CO2 + eGFR
MoCA Score	PMM	Base Set + BMI + HDL + CO2 + eGFR + log UACR
Logical Memory (LM) form II	PMM	Base Set + BMI + HDL + CO2 + eGFR + log UACR + MoCA
Digital Symbol Coding Score (DCS)	PMM	Base Set + BMI + HDL + CO2 + eGFR + log UACR + MoCA + LM
VR-12 Physical Component Summary Score (VR-12 PCS)	Linear	Base Set + BMI + HDL + CO2 + eGFR + log UACR + MoCA + LM + DSC
VR-12 Mental Component Summary Score (VR-12 MCS)	Linear	Base Set + BMI + HDL + CO2 + eGFR + log UACR + MoCA + LM + DSC + VR-12 PCS
PHQ-9 ≥ 10	Linear	Base Set + BMI + HDL + CO2 + eGFR + log UACR + MoCA + LM + DSC + VR-12 PCS + VR-12 MCS

© 2023 Rashid T et al. *JAMA Network Open.*

eTable 12. Changes in White Matter Lesions (WML) Derived in Deep White Matter (DWM) Regions-of-Interest (ROIs) Between Intensive Treatment Group and Standard Treatment Group After Multiple Imputation

Label – ROI Name	Effect Size	P-Value	P-Value (FDR)	Mean Change (Intensive Treatment Group) (95% CI)	Mean Change (Standard Treatment Group) (95% CI)	Mean at Baseline (Intensive Treatment Group) (95% CI)	Mean at Follow-up (Intensive Treatment Group) (95% CI)	Mean at Baseline (Standard Treatment Group) (95% CI)	Mean at Follow-up (Standard Treatment Group) (95% CI)
94 - Posterior limb of internal capsule right	6.88	< 0.001	< 0.001	0.11 (-0.01 to 0.24)	0.72 (0.59 to 0.85)	5.14 (2.02 to 8.27)	6 (2.58 to 9.42)	2.22 (0.4 to 4.04)	4.24 (2.26 to 6.22)
56 - Tapatum left	6.84	< 0.001	< 0.001	1.21 (0.89 to 1.54)	2.71 (2.41 to 3)	72.07 (55.49 to 88.65)	84.15 (67 to 101.3)	72.31 (53.57 to 91.05)	100.99 (80.01 to 121.97)
113 - Splenium of corpus callosum right	6.68	< 0.001	< 0.001	1.99 (1.6 to 2.38)	3.67 (3.34 to 4)	174.39 (128.92 to 219.87)	219.65 (168.75 to 270.56)	135.31 (97.3 to 173.33)	221.72 (174.76 to 268.68)
43 - Superior fronto-occipital fasciculus left	6.6	< 0.001	< 0.001	0.65 (0.42 to 0.89)	1.73 (1.5 to 1.96)	11.15 (8.28 to 14.02)	14.52 (11.3 to 17.74)	10.94 (7.88 to 14)	20.69 (16.06 to 25.33)
38 - Posterior corona radiata left	6.56	< 0.001	< 0.001	1.64 (1.31 to 1.97)	3.11 (2.8 to 3.42)	115.86 (83.59 to 148.12)	141.58 (106.7 to 176.47)	93.34 (64.77 to 121.9)	149.38 (114.67 to 184.1)
36 - Anterior corona radiata left	5.72	< 0.001	< 0.001	1.8 (1.38 to 2.22)	3.37 (2.99 to 3.76)	134.23 (109.06 to 159.41)	165.9 (136.57 to 195.24)	119.68 (95.27 to 144.09)	203.68 (165.8 to 241.55)
115 - Tapatum right	5.46	< 0.001	< 0.001	1 (0.69 to 1.3)	2.16 (1.85 to 2.47)	45.65 (35.18 to 56.11)	55.19 (43.8 to 66.58)	45.47 (34.63 to 56.31)	64.75 (52.2 to 77.29)
112 - Body of corpus callosum right	5.35	< 0.001	< 0.001	1.33 (0.97 to 1.68)	2.59 (2.24 to 2.93)	80.49 (60.18 to 100.8)	100.74 (76.54 to 124.94)	63.85 (46.11 to 81.6)	108.29 (82.24 to 134.34)
53 - Splenium of corpus callosum left	5.22	< 0.001	< 0.001	2.27 (1.9 to 2.65)	3.59 (3.25 to 3.93)	183.88 (138.14 to 229.62)	231.68 (180.8 to 282.56)	156.51 (113.69 to 199.34)	245.64 (194.71 to 296.56)
98 - Posterior corona radiata right	5.15	< 0.001	< 0.001	2.07 (1.74 to 2.4)	3.21 (2.89 to 3.53)	136.98 (97.18 to 176.77)	178.07 (132.76 to 223.38)	111.44 (77.21 to 145.67)	184.69 (144.15 to 225.23)
52 - Body of corpus callosum left	4.96	< 0.001	< 0.001	1.41 (1.09 to 1.74)	2.46 (2.15 to 2.77)	62.68 (43.01 to 82.35)	86.88 (62.22 to 111.54)	49.36 (32.43 to 66.3)	86.96 (62.39 to 111.53)
97 - Superior corona radiata right	4.94	< 0.001	< 0.001	2.19 (1.82 to 2.56)	3.33 (2.99 to 3.68)	188.54 (126.4 to 250.67)	256.06 (175.19 to 336.93)	124.56 (75.78 to 173.33)	231.07 (163.81 to 298.32)
35 - Posterior thalamic radiation left	4.67	< 0.001	< 0.001	2.33 (1.89 to 2.77)	3.73 (3.31 to 4.15)	305.91 (242.87 to 368.95)	361.42 (292.11 to 430.73)	305.95 (240.42 to 371.49)	420.34 (345.89 to 494.79)
39 - Cingulum (cingulate gyrus) left	4.6	< 0.001	< 0.001	0.06 (-0.01 to 0.14)	0.35 (0.23 to 0.48)	0.72 (0.36 to 1.08)	0.79 (0.41 to 1.17)	1.31 (0.54 to 2.09)	2.53 (1.06 to 4.01)
37 - Superior corona radiata left	4.42	< 0.001	< 0.001	2.23 (1.86 to 2.6)	3.25 (2.91 to 3.6)	162.93 (110.3 to 215.57)	226.64 (162.93 to 290.34)	123.29 (77.08 to 169.49)	228.77 (164.97 to 292.57)
33 - Anterior limb of internal capsule left	4.19	< 0.001	< 0.001	0.96 (0.75 to 1.18)	1.6 (1.35 to 1.85)	17.53 (9.28 to 25.79)	23.18 (14.33 to 32.03)	18.03 (9.46 to 26.6)	31.62 (20.79 to 42.46)
111 - Genu of corpus callosum right	4.09	< 0.001	< 0.001	1.28 (0.94 to 1.63)	2.21 (1.86 to 2.56)	48.04 (39.65 to 56.42)	61.72 (50.93 to 72.51)	46.3 (36.77 to 55.82)	69.42 (57.09 to 81.74)
102 - Superior longitudinal fasciculus right	4.09	< 0.001	< 0.001	1.29 (1.03 to 1.56)	2.04 (1.77 to 2.31)	33.06 (20.08 to 46.04)	47.14 (32.01 to 62.28)	27.55 (16.28 to 38.82)	45.24 (31.55 to 58.92)

© 2023 Rashid T et al. *JAMA Network Open.*

95 - Posterior thalamic radiation right	3.81	< 0.001	< 0.001	2.3 (1.85 to 2.74)	3.46 (3.02 to 3.89)	267.03 (215.59 to 318.48)	319.12 (261.91 to 376.33)	256.32 (207.51 to 305.12)	365.07 (305.48 to 424.66)
99 - Cingulum (cingulate gyrus) right	-3.63	< 0.001	0.001	0.3 (0.16 to 0.44)	-0.01 (-0.09 to 0.07)	1.66 (0.23 to 3.09)	1.98 (0.78 to 3.18)	0.68 (0.26 to 1.09)	0.61 (0.27 to 0.94)
42 - Superior longitudinal fasciculus left	3.44	0.001	0.001	1.47 (1.21 to 1.74)	2.08 (1.81 to 2.36)	35.84 (22.83 to 48.86)	49.92 (34.56 to 65.28)	34.26 (19.03 to 49.49)	55.69 (36.26 to 75.12)
100 - Cingulum (hippocampus) right	2.77	0.006	0.011	-0.04 (-0.07 to 0)	0.02 (0 to 0.03)	0.1 (-0.02 to 0.22)	0 (-0.02 to 0.02)	0 (0 to 0)	0.02 (0 to 0.04)
41 - Fornix(cres) Stria terminalisleft	2.49	0.013	0.023	-0.04 (-0.09 to 0.01)	0.04 (0 to 0.07)	0.23 (0.08 to 0.38)	0.15 (0.03 to 0.26)	0.1 (-0.08 to 0.28)	0.14 (0.01 to 0.28)
106 - External capsule right	2.43	0.016	0.026	0.69 (0.48 to 0.9)	1 (0.79 to 1.21)	11.86 (6.36 to 17.36)	14.39 (9.21 to 19.56)	9.45 (5.03 to 13.87)	13.63 (8.81 to 18.45)
96 - Anterior corona radiata right	2.37	0.018	0.029	2.17 (1.85 to 2.48)	2.65 (2.3 to 3)	58.59 (39.16 to 78.01)	89.67 (66.84 to 112.51)	51 (34.68 to 67.31)	113.96 (80 to 147.91)
51 - Genu of corpus callosum left	2.15	0.032	0.049	1.24 (0.99 to 1.5)	1.56 (1.3 to 1.82)	12.94 (9.14 to 16.74)	20.96 (15.51 to 26.42)	12.16 (7.81 to 16.52)	20.84 (14.88 to 26.8)
46 - External capsule left	2.01	0.045	0.067	0.51 (0.25 to 0.76)	0.88 (0.6 to 1.16)	9.89 (5.49 to 14.28)	11.72 (7.7 to 15.75)	10.82 (6.04 to 15.6)	15.04 (11.06 to 19.03)
40 - Cingulum (hippocampus) left	1.99	0.047	0.067	-0.02 (-0.04 to 0.01)	0.01 (0 to 0.03)	0.21 (0 to 0.42)	0.18 (-0.02 to 0.38)	0.02 (-0.02 to 0.06)	0.03 (0 to 0.06)
103 - Superior fronto-occipital fasciculus right	1.89	0.059	0.082	0.59 (0.44 to 0.73)	0.74 (0.6 to 0.89)	2.71 (1.35 to 4.07)	4.67 (2.79 to 6.56)	1.99 (0.96 to 3.02)	3.98 (2.46 to 5.51)
101 - Fornix(cres) Stria terminalis right	-1.69	0.091	0.122	0.12 (0.06 to 0.18)	0.04 (-0.01 to 0.1)	0.14 (0.04 to 0.23)	0.45 (0.09 to 0.81)	0.12 (0.05 to 0.19)	0.23 (0.06 to 0.39)
93 - Anterior limb of internal capsule right	1.67	0.095	0.122	0.87 (0.69 to 1.04)	1.06 (0.85 to 1.27)	10.46 (4.61 to 16.32)	15.25 (8.33 to 22.16)	6.55 (2.24 to 10.85)	12.84 (7.66 to 18.02)
45 - Sagittal stratum left	-1.58	0.114	0.143	0.28 (0.08 to 0.48)	0.09 (-0.06 to 0.24)	7.91 (2.33 to 13.49)	10.85 (3.45 to 18.25)	5.87 (2.91 to 8.84)	6.39 (3.26 to 9.52)
105 - Sagittal stratum right	1.48	0.139	0.169	0.22 (0.05 to 0.39)	0.39 (0.25 to 0.54)	7.56 (1.01 to 14.11)	10.03 (1.16 to 18.9)	5.27 (2.57 to 7.96)	6.46 (3.59 to 9.33)
50 - Fornix (column and body) left	1.2	0.229	0.269	-0.01 (-0.04 to 0.01)	0 (-0.01 to 0.01)	0.04 (-0.04 to 0.12)	0 (-0.01 to 0.01)	0.01 (-0.01 to 0.02)	0.01 (0 to 0.02)
104 - Inferior fronto-occipital fasciculus right	-0.81	0.419	0.479	0.23 (0.11 to 0.35)	0.13 (-0.01 to 0.26)	2.91 (1.27 to 4.55)	3.33 (1.69 to 4.97)	2.98 (1.65 to 4.3)	3.09 (1.87 to 4.31)
44 - Inferior fronto-occipital fasciculus left	-0.77	0.442	0.491	0.41 (0.29 to 0.54)	0.32 (0.21 to 0.44)	2.75 (1.43 to 4.06)	3.89 (2.33 to 5.45)	2.07 (1.04 to 3.09)	2.7 (1.62 to 3.79)
54 - Retrolenticular part of internal capsule left	0.63	0.53	0.573	0.18 (0.06 to 0.3)	0.23 (0.13 to 0.32)	3.36 (0.79 to 5.94)	4.4 (0.6 to 8.2)	2.33 (0.57 to 4.1)	2.83 (1 to 4.66)
110 - Fornix (column and body) right	0.3	0.767	0.807	-0.04 (-0.09 to 0.01)	-0.03 (-0.08 to 0.02)	0.22 (0.02 to 0.43)	0.12 (0.04 to 0.2)	0.25 (0.06 to 0.44)	0.17 (0.02 to 0.33)
114 - Retrolenticular part of internal capsule right	-0.23	0.818	0.827	0.43 (0.2 to 0.66)	0.39 (0.24 to 0.54)	6.32 (1.43 to 11.2)	8.36 (2.96 to 13.77)	3.74 (1.77 to 5.7)	4.98 (2.69 to 7.26)
34 - Posterior limb of internal capsule left	0.22	0.827	0.827	0.72 (0.55 to 0.89)	0.71 (0.54 to 0.88)	4.05 (2.11 to 5.99)	7.88 (4.97 to 10.79)	3.61 (1.85 to 5.38)	6.43 (4.18 to 8.67)

© 2023 Rashid T et al. *JAMA Network Open.*

Abbreviations: FDR, false discovery rate; CI, confidence interval; WM, white matter; GM, gray matter; DWM, deep white matter; SWM, superficially-located white matter; DTI, diffusion tensor imaging; FA, fractional anisotropy; MD, mean diffusivity, ROI, region-of-interest

© 2023 Rashid T et al. *JAMA Network Open.*

eReferences.

1. Group SR. A randomized trial of intensive versus standard blood-pressure control. *New England Journal of Medicine*. 2015;373:2103-2116.
2. Ambrosius WT, Sink KM, Foy CG, Berlowitz DR, Cheung AK, Cushman WC, Fine LJ, Goff Jr DC, Johnson KC and Killeen AA. The design and rationale of a multicenter clinical trial comparing two strategies for control of systolic blood pressure: the Systolic Blood Pressure Intervention Trial (SPRINT). *Clinical Trials*. 2014;11:532-546.
3. Wang Z, Aguirre GK, Rao H, Wang J, Fernández-Seara MA, Childress AR and Detre JA. Empirical optimization of ASL data analysis using an ASL data processing toolbox: ASLtbx. *Magnetic resonance imaging*. 2008;26:261-269.
4. Alsop DC, Detre JA, Golay X, Günther M, Hendrikse J, Hernandez-Garcia L, Lu H, MacIntosh BJ, Parkes LM, Smits M, van Osch MJP, Wang DJJ, Wong EC and Zaharchuk G. Recommended implementation of arterial spin-labeled perfusion MRI for clinical applications: A consensus of the ISMRM perfusion study group and the European consortium for ASL in dementia. *Magnetic resonance in medicine*. 2015;73:102-116.
5. Dolui S, Wang Z, Shinohara RT, Wolk DA, Detre JA and Initiative AsDN. Structural Correlation-based Outlier Rejection (SCORE) algorithm for arterial spin labeling time series. *Journal of Magnetic Resonance Imaging*. 2017;45:1786-1797.
6. Dolui S, Wolk DA and Detre JA. SCRUB: a structural correlation and empirical robust bayesian method for ASL data. *Proceedings of the International Society of Magnetic Resonance in Medicine*. 2016.
7. Dolui S, Wolf R, Nabavizadeh SA, Wolk DA and Detre JA. Automated quality evaluation index for 2D ASL CBF maps. *ISMRM 25th Annual Meeting and Exhibition; Apr 22-27, 2017; Hawaii, USA*. 2017.
8. Doshi J, Erus G, Ou Y, Resnick SM, Gur RC, Gur RE, Satterthwaite TD, Furth S, Davatzikos C and Initiative AsN. MUSE: MUlti-atlas region Segmentation utilizing Ensembles of registration algorithms and parameters, and locally optimal atlas selection. *Neuroimage*. 2016;127:186-195.
9. Tustison NJ, Avants BB, Cook PA, Zheng Y, Egan A, Yushkevich PA and Gee JC. N4ITK: improved N3 bias correction. *IEEE transactions on medical imaging*. 2010;29:1310.
10. Doshi J, Erus G, Habes M and Davatzikos C. DeepMRSeg: A convolutional deep neural network for anatomy and abnormality segmentation on MR images. *arXiv preprint arXiv:190702110*. 2019.
11. Ronneberger O, Fischer P and Brox T. U-net: Convolutional networks for biomedical image segmentation. *International Conference on Medical image computing and computer-assisted intervention*. 2015:234-241.
12. Oishi K, Faria A, Jiang H, Li X, Akhter K, Zhang J, Hsu JT, Miller MI, van Zijl PC and Albert M. Atlas-based whole brain white matter analysis using large deformation diffeomorphic metric mapping: application to normal elderly and Alzheimer's disease participants. *Neuroimage*. 2009;46:486-499.
13. Avants BB, Tustison N and Song G. Advanced normalization tools (ANTS). *Insight j*. 2009;2:1-35.
14. Maillard P, Fletcher E, Singh B, Martinez O, Johnson DK, Olichney JM, Farias ST and DeCarli C. Cerebral white matter free water: A sensitive biomarker of cognition and function. *Neurology*. 2019;92:e2221-e2231.
15. Smith SM, Jenkinson M, Woolrich MW, Beckmann CF, Behrens TE, Johansen-Berg H, Bannister PR, De Luca M, Drobnjak I and Flitney DE. Advances in functional and structural MR image analysis and implementation as FSL. *Neuroimage*. 2004;23:S208-S219.
16. Jenkinson M, Beckmann CF, Behrens TE, Woolrich MW and Smith SM. Fsl. *Neuroimage*. 2012;62:782-790.
17. Smith SM. Fast robust automated brain extraction. *Human brain mapping*. 2002;17:143-155.

© 2023 Rashid T et al. *JAMA Network Open.*

18. Garyfallidis E, Brett M, Amirbekian B, Rokem A, Van Der Walt S, Descoteaux M and Nimmo-Smith I. Dipy, a library for the analysis of diffusion MRI data. *Frontiers in neuroinformatics*. 2014;8:8.
19. Nasrallah IM, Pajewski NM, Auchus AP, Chelune G, Cheung AK, Cleveland ML, Coker LH, Crowe MG, Cushman WC and Cutler JA. Association of intensive vs standard blood pressure control with cerebral white matter lesions. *Jama*. 2019;322:524-534.
20. Little RJ and Rubin DB. *Statistical analysis with missing data*: John Wiley & Sons; 2019.

© 2023 Rashid T et al. *JAMA Network Open.*
