# Supplementary material for: Association of Intensive vs Standard Blood Pressure Control With Regional Changes in Cerebral Small Vessel Disease Biomarkers: Post Hoc Secondary Analysis of the SPRINT MIND Randomized Clinical Trial
Source: JAMA Netw Open. 2023 Mar 1;6(3):e231055. doi: 10.1001/jamanetworkopen.2023.1055 (PMC9978954; doi:10.1001/jamanetworkopen.2023.1055)
Supplement: Supplement 3. — Data Sharing Statement [file jamanetwopen-e231055-s003.pdf]

Data Sharing Statement

Rashid. Association of Intensive vs Standard Blood Pressure Control With Regional Changes in Cerebral Small Vessel Disease Biomarkers. *JAMA Netw Open*. Published March 01, 2023. doi:10.1001/jamanetworkopen.2023.1055

Data
Data available: No

Additional Information
Explanation for why data not available: This is a secondary analysis of data from the SPRINT randomized trial. Summary magnetic resonance imaging (MRI) outcomes from the trial are available at https://biolincc.nhlbi.nih.gov/studies/sprint/. Due to complexities with de-identification, raw MRI images are not contained in this repository, nor are the regional measures derived as part of this study.
